# Which interventions for alcohol use should be included in a universal healthcare benefit package? An umbrella review of targeted interventions to address harmful drinking and dependence

**DOI:** 10.1186/s12889-023-15152-6

**Published:** 2023-02-23

**Authors:** Siobhan Botwright, Jiratorn Sutawong, Pritaporn Kingkaew, Thunyarat Anothaisintawee, Saudamini Vishwanath Dabak, Chotika Suwanpanich, Nattiwat Promchit, Roongnapa Kampang, Wanrudee Isaranuwatchai

**Affiliations:** 1 Health Intervention and Technology Assessment Program, Department of Health, Ministry of Public Health, Tiwanon Rd, 6Th Floor, 6Th Building, Muang, 11000 Nonthaburi Thailand; 2 Institute of Health Policy, Management and Evaluation, University of Toronto, Toronto, Canada

**Keywords:** Alcohol use disorder, Umbrella review, Interventions, Harmful drinking, Abstinence, Universal health coverage

## Abstract

**Background:**

This study aimed to identify targeted interventions for the prevention and treatment of harmful alcohol use. Umbrella review methodology was used to summarise the effectiveness across a broad range of interventions, in order to identify which interventions should be considered for inclusion within universal health coverage schemes in low- and middle-income countries.

**Methods and findings:**

We included systematic reviews with meta-analysis of randomised controlled trials (RCTs) on targeted interventions addressing alcohol use in harmful drinkers or individuals with alcohol use disorder. We only included outcomes related to alcohol consumption, heavy drinking, binge drinking, abstinence, or alcohol-attributable accident, injury, morbidity or mortality. PubMed, Embase, PsycINFO, Cochrane Database of Systematic Reviews, and the International HTA Database were searched from inception to 3 September 2021. Risk of bias of reviews was assessed using the AMSTAR2 tool. After reviewing the abstracts of 9,167 articles, results were summarised narratively and certainty in the body of evidence for each intervention was assessed using GRADE. In total, 86 studies met the inclusion criteria, of which the majority reported outcomes for brief intervention (30 studies) or pharmacological interventions (29 studies). Overall, methodological quality of included studies was low.

**Conclusions:**

For harmful drinking, brief interventions, cognitive behavioural therapy, and motivational interviewing showed a small effect, whereas mentoring in adolescents and children may have a significant long-term effect. For alcohol use disorder, social network approaches and acamprosate showed evidence of a significant and durable effect. More evidence is required on the effectiveness of gamma-hydroxybutyric acid (GHB), nalmefene, and quetiapine, as well as optimal combinations of pharmacological and psychosocial interventions. As an umbrella review, we were unable to identify the extent to which variation between studies stemmed from differences in intervention delivery or variation between country contexts. Further research is required on applicability of findings across settings and best practice for implementation.

Funded by the Thai Health Promotion Foundation, grant number 61–00-1812.

**Supplementary Information:**

The online version contains supplementary material available at 10.1186/s12889-023-15152-6.

## Introduction

Alcohol use is the leading risk factor for premature death and disability among adults aged 15–49 years globally, contributing to 2.8 million deaths in 2016 [1]. While targeted policies and interventions over the past decade have successfully reduced population-level alcohol consumption in high-income countries, alcohol use continues to rise in low-income and middle-income countries (LMICs) [1, 2], contributing to a 5% increase in the number of disability-adjusted life years (DALYs) attributable to alcohol use globally between 2007 and 2017 [3]. Risk of all-cause mortality and cancers has been shown to increase proportionately with alcohol consumption. Hence, without effective measures to curb its use, alcohol is likely to place an increasing burden on LMIC health systems [1].

Harmful alcohol use refers to people experiencing detrimental health and social consequences because of their drinking, which extends to the people around them and society as a whole [4], whereas alcohol use disorders represent a sub-set of harmful drinkers, who experience chronic relapsing brain disorder with an impaired ability to stop or control alcohol use despite adverse social, occupational, or health consequences [5]. At the population level, there is a considerable body of evidence to suggest that alcohol taxation and pricing control are the most effective interventions to decrease alcohol consumption and heavy drinking, and this finding appears to be consistent across geographies and socio-economic groups [6–8]. Such policies are long-term measures to prevent initiation of drinking and reduce alcohol consumption, whereas short-term measures seek to reduce adverse health and social consequences among current drinkers [6]. The effectiveness of many short-term measures is likely to be context-specific, especially when applied to LMICs with varying health systems and cultural differences [9, 10]. This can present challenges for policymakers in interpreting the often conflicting body of evidence to identify which interventions to fund in their setting.

For countries with universal health coverage (UHC) schemes, policymakers must decide on the package of interventions to provide for harmful alcohol use. In many high-income countries, health technology assessment agencies employ horizon scanning to identify new technologies and treatments to provide to the population [11]. However, for LMICs with nascent UHC schemes or looking to improve the package of services provided under UHC, there may be a need to scope all available intervention options that are available and shortlist those that merit further evaluation for inclusion under UHC. Umbrella reviews, or overviews of systematic reviews, have a broader scope than individual systematic reviews or meta-analyses, and can therefore examine a broad range of interventions aligned with choices facing policymakers [12]. Moreover, umbrella reviews require fewer resources to conduct, compared with undertaking a series of systematic reviews for a clinical area, which makes umbrella reviews more feasible for informing policy choices in resource-constrained settings. In response to a request from the Thai Health Promotion Foundation to review effective interventions to address harmful alcohol use, we therefore employed an umbrella review methodology to scope interventions that may warrant further assessment for inclusion or removal from the Thai universal health coverage scheme.

The aim of this review was to identify targeted interventions for the prevention and treatment of harmful alcohol use, and to summarise the evidence of their effectiveness. We defined targeted interventions as those that were based on prior risk screening [13]. We had the following two research questions:

1. Which interventions are effective at reducing alcohol use among individuals identified as having, or being at risk of, harmful alcohol use?
2. Which interventions are effective for the treatment of alcohol use disorder?

Although the main purpose of this study was to inform policy in Thailand, we expect that our review will prove generalisable to other settings, especially as we explore umbrella review methodology as a pragmatic approach by which to shortlist interventions for further assessment during the benefit package selection process in LMICs.

## Methods

We conducted an umbrella review, or review of systematic reviews. The protocol for this study was developed a priori, registered in the International Prospective Register of Systematic Reviews (PROSPERO) (CRD42017083412) and published as a pre-print [14]. The study was developed following the Cochrane Handbook for Systematic Reviews of Interventions [15] and adheres to the Preferred Reporting Items for Overviews of Reviews (PRIOR) guideline (Supplement 1) [16] and PRISMA-S checklist for reporting literature searches (Supplement 2) [17].

### Eligibility criteria and search strategy

We searched MEDLINE via PubMed, Embase, PsycINFO, Cochrane Database of Systematic Reviews, and the International HTA Database from inception to 3 September 2021. Search strategies for each database are provided in Supplement 3. No language restrictions were applied and no published search filters were used. We further searched the reference lists of identified studies.

We included articles that met the following criteria: (1) participants were identified as risky drinkers, harmful/hazardous drinkers, or diagnosed with alcohol use disorder or dependency; (2) interventions were targeted interventions (i.e. based on prior risk screening [13]) and primarily intended to address alcohol use; (3) outcomes were related to alcohol consumption, heavy drinking, binge drinking, abstinence, or alcohol-attributable accident, injury, morbidity or mortality; (4) studies were systematic reviews with meta-analysis of randomised controlled trials (RCTs), for which the results from alcohol RCTs were presented separately and not combined with other interventions or study types. We chose to only include meta-analyses to facilitate comparison of quantitative data across reviews (especially given the very broad scope of the study), and we restricted inclusion to RCTs to reduce risk of bias [18]. We excluded any articles that were universal preventive interventions, which are provided to everyone regardless of individual risk [13], and meta-analyses that either did not meet the criteria for a systematic review [19] or for which only one RCT was included in the analysis. We accepted any comparator or setting.

### Selection and data extraction

Reviews retrieved from the database search were imported into Covidence (systematic review management software). After removal of duplicates, articles were screened independently and in duplicate according to the eligibility criteria, first by title/abstract and then by full text. Eligibility criteria were piloted by all authors and refined before screening. Any conflicts were resolved by a third reviewer.

A data extraction form was developed in Excel and piloted by all authors before starting data extraction. One-third of papers were extracted independently in duplicate. Since there was good concordance among the articles extracted in duplicate, the remaining articles were extracted by a single reviewer and validated by a second reviewer. The data extraction form included citation details, purpose, eligibility criteria, meta-analysis methods, summary of meta-analysis results (including effect size, heterogeneity, publication bias, risk of bias), and main conclusions. Missing or unclear data were left blank.

### Quality assessment

Methodological quality of included reviews was assessed using the A MeaSurement Tool to Assess systematic Reviews (AMSTAR) 2, which comprises 16 domains and provides a rating from ‘High’ to ‘Critically low’ [20]. For each article, the domains of the AMSTAR 2 tool were assessed independently by two reviewers, with conflict resolution by a third reviewer. Before carrying out the quality assessment, authors came to a consensus on the AMSTAR 2 domains that were considered critical/non-critical, in order to enhance consistency in the final ratings across reviewers. Domains that were considered critical are highlighted in bold in Fig. 2 and comprise the following: protocol established a priori, comprehensive search strategy, excluded studies listed and justified, satisfactory technique to assess risk of bias (RoB), appropriate methods for meta-analysis, RoB of individual studies discussed in results, publication bias investigated and discussed.

### Synthesis of results

Outcomes from each meta-analysis were grouped by type of intervention (Table 1), using an adapted version of the classification from the International Standards for the Treatment of Drug Use Disorders [21], and further stratified by population, comparator, and outcome for narrative analysis. Risk of bias from primary studies was reported without re-assessment by study authors. Similarly, we reported discrepant data without seeking to manage discrepant results and reported publication bias where assessed in the meta-analysis, but did not undertake additional analysis. Forest plots were not used to summarise results because of the heterogeneity in effect sizes, populations, methods to deliver interventions, and outcomes across studies. Overlap between studies was not assessed using a citation matrix because the primary purpose of this review was intended to identify interventions for which there is evidence of an effect, and not to assess relative effectiveness of interventions. Certainty in the body of evidence for each intervention category was assessed using GRADE criteria modified for an umbrella review [22, 23].

**Table 1:** Classification of interventions within the scope of this review, adapted from the World Health Organization (WHO)/United Nations Office on Drugs and Crime (UNODC) International Standards for the Treatment of Drug Use Disorders [21]

	**Category**	**Sub-category**	**Definition**
1	Screening, brief intervention and referral	1.1 Screening	A brief process to identify indicators for the presence of alcohol use disorder
		1.2 Brief intervention	A structured therapy of short duration (typically 5–30 min) with the aim of helping an individual cease or reduce their alcohol consumption
		1.3 Referral to treatment	Interventions to speed up or reduce drop-out during referral to treatment, in individuals assessed to have clinically significant harmful alcohol use
2	Psychosocial interventions	2.1 Cognitive behavioural therapy	Patients are introduced to new coping skills and cognitive strategies to replace maladaptive behavioural and thinking patterns
		2.2 Contingency management	Patients are given concrete rewards to reinforce positive behaviours, such as abstinence, treatment attendance, or compliance with medication
		2.3 Community reinforcement approach	Patients seek to modify the way in which they interact with their community in order to gain positive reinforcement, for example through family interactions, healthy social activities, or employment
		2.4 Motivational interviewing/ enhancement	Patients increase their motivation to change a behaviour, through collaborative sessions with a clinician that recognise autonomy of the patient
		2.5 Family-oriented treatment approach	A collection of methods that utilise family relationships to positively influence the behaviour of an individual with alcohol use disorder. Families and caregivers may participate in and support the treatment process
		2.6 Mutual help group	Patients participate in groups that provide information, structured activities and peer support in a non-judgemental environment
3	Pharmacological interventions	3.1 Anticonvulsants	e.g. carbamazepine, gabapentin, topiramate
		3.2 Antidepressants	e.g. sertraline, citalopram
		3.3 Antipsychotics	e.g. tiapride, cyametazine
		3.4 Aversive agents	e.g. disulfiram
		3.5 Baclofen	
		3.6 Benzodiazepines	e.g. diazepam, oxazepam, chlordiazepoxide
		3.7 Acamprosate	Glutamate antagonist
		3.8 Opioid antagonist	e.g. naltrexone, nalmefene
		3.9 Other	e.g. GHB (gamma hydroxybutyric acid), nitrous oxide
4	Treatment package	4.1 Psychosocial	More than one psychosocial intervention administered over the same treatment period
		4.2 Pharmacological	More than one pharmacological intervention administered over the same treatment period
		4.3 Psychosocial and pharmacological	A combination of psychosocial and pharmacological interventions administered over the same treatment period
5	Miscellaneous	e.g. yoga, acupuncture, physical activity	

### Validation of results

To identify whether the umbrella review approach provided a comprehensive overview of the effectiveness of alcohol interventions, we compared our results with the National Institute for Health and Care Excellence (NICE) clinical guideline for harmful drinking and alcohol dependence in the United Kingdom [24]. We chose to use a clinical guideline for comparison in order to cover the full scope of our umbrella review, whilst recognising that clinical practice guideline development follows a different process to our review, most notably by including extensive stakeholder consultation with clinicians, patients, the public, and other groups. NICE was selected as an agency recognised for its methodological rigour. We additionally compared results against guidelines from Australia, Canada, Germany, and the USA (other countries with publicly available guidelines accompanied by evidence and evidence ratings for each recommendation) to verify whether the NICE guideline is similar to other countries [25–29].

## Results

The following sections present the characteristics of included studies and assessment of methodological quality, followed by findings for each type of intervention and a comparison of findings with clinical practice guidelines.

### Characteristics of included studies

**Fig. 1:**
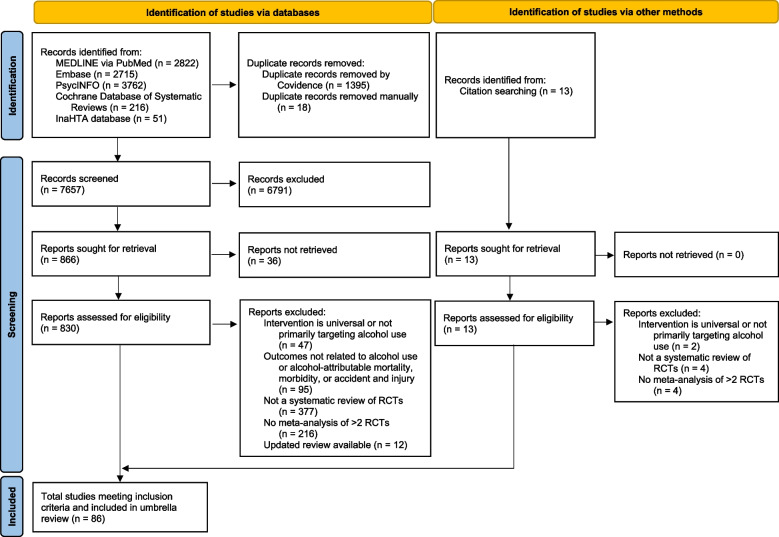
PRISMA diagram of study selection process

The initial search yielded 9,167 articles, of which 1,413 were duplicates. Following title and abstract screening, 829 studies underwent full text review, yielding 86 studies for the review (Fig. 1). Most of the studies excluded during title/abstract screening did not report on an intervention primarily aimed at reducing or preventing alcohol use (Supplement 7). The characteristics of the included studies are detailed in Table 2 (with full details available in Supplement 4) and studies excluded during full text screening are listed in Supplement 5.

**Table 2:** Characteristics of included studies

**Author, year**	**Study type**	**Population** (alcohol consumption; specific group)	**Intervention**	**Setting**	**Funding source**	**Conflict of interest to declare**
Screening, brief intervention, and/or referral to treatment						
Beich, 2003 [30]	DMA	Excessive alcohol use	Screening, brief intervention	General practice settings	Government	No
Ballesteros, 2004 [31]	DMA	Hazardous drinkers	Brief interventions as applied in primary care settings	primary care	NR	NR
Bendtsen, 2021 [32]	DMA	Risky drinker (harmful and hazardous); any population	Text messaging	Any	NR	NR
Bertholet, 2005 [33]	DMA	Risky drinker; individuals attending primary care facilities but not seeking help for alcohol-related problems	Brief intervention	Primary care facilities	University	NR
Carney, 2016 [34]	DMA	Used alcohol or other drugs, did not meet criteria for dependence but had faced negative behavioural consequences due to substance use; adolescents under the age of 19 attending high school, secondary school, or further education training college	Brief school-based intervention	High schools or further education training colleges	Research council, not-for-profit foundation	No
Dedert, 2014 [35]	DMA	Alcohol misuse or AUD; adults aged 18 years or over (excluding pregnant women)	e-interventions	Outpatients in any setting or patients enrolled through self-assessment	Government	No
Doherty, 2017 [36]	DMA	Hazardous or harmful alcohol use; adult military and veteran	Brief intervention	Web delivered out-patient setting	NR	No
Donoghue, 2014 [37]	DMA	Consuming alcohol to a hazardous level; non-treatment seeking	Electronic screening and brief intervention [eSBI]	Health care settings, including primary care and the emergency department	Government	No
Elzerbi, 2015 [38]	DMA	Non-treatment-seeking and hazardous or harmful drinking (average consumption 20–40 g and > 40 g of alcohol per day for women and 40–60 g and > 60 g per day for men); aged 18–64 years	Brief intervention	Primary health care or emergency department	NR	No
Elzerbi, 2017 [39]	DMA	Hazardous or harmful drinking	Brief intervention	Emergency department settings	None	No
Fachini, 2012 [40]	DMA	College students engaged in heavy episodic drinking	Brief intervention [BASICS programme]	Public universities	NR	No
Foxcroft, 2015 [41]	DMA	NR; university and college students	Social norms interventions	Colleges or universities	University, government	Yes
Gilligan, 2019 [42]	DMA	NR; school-aged children (< = 18 years)	Family-based prevention programmes	Communities and schools	None	Yes
Hennessy, 2019 [43]	NMA	NR; college students (≤ 30 years)	Various type of brief interventions	Colleges or universities	Government	Yes
Jonas, 2012 [44]	DMA	Adults with risky drinking; adolescents with alcohol misuse identified by screening in primary care settings	Screening followed by behavioural counselling, with or without referral	Primary care settings	Government	NR (Disclosure forms not available)
Kaner, 2017 [45]	DMA	People living in the community whose alcohol consumption had been screened as hazardous or harmful	Digital brief intervention	Community	University	Yes
Kaner, 2018 [46]	DMA	People with hazardous or harmful alcohol consumption as identified by a screening tool	Brief intervention	Emergency care or other primary care settings	University	No
Kohler, 2015 [47]	DMA	Existing alcohol use problems	Motivational interviewing (MI), delivered in a brief intervention during an emergency care contact	Emergency departments	NR	No
MacArthur, 2018 [48]	DMA	Multiple risk behaviours; aged up to 18 years and/or their parents, guardians, carers, peers, and/or school members	Targeted multiple risk behaviour interventions (brief interventions)	School-based interventions, (home, kindergarten, primary school, secondary school, clinic, community)	Government	Yes
McQueen, 2011 [49]	DMA	Heavy alcohol users	Brief interventions for heavy alcohol users	General hospital setting	Government, research network	No
Mujcic, 2020 [50]	DMA	Drank alcohol in the past week; cancer survivors	Distance-based alcohol moderation	Distance-based	Not-for-profit society	Yes
O’Connor, 2018 [51]	DMA	Non dependence alcohol user; aged 12 years or older	Screening and behavioural counselling	Primary care	NR	No
Prestwich, 2016 [52]	DMA	All drinking behaviour	Brief intervention (face to face and computer delivered)	Educational settings, medical and community settings	Government	No
Saxton, 2021 [53]	DMA	Hazardous alcohol use; 16 years and older	Personalised normative feedback interventions	Delivered to individuals, not in-person	Government	Yes
Riper, 2009 [54]	DMA	Problem drinkers	Brief, single-session personalized-feedback interventions without therapeutic guidance	NR	NR	Yes
Smedslund, 2017 [55]	DMA	High or risky consumers of alcohol; young people between 15 and 25	Brief intervention	NR	University	No
Steele, 2020 [56]	DMA, NMA	Alcohol use disorder or problematic alcohol use; adolescents (12 to 20 years)	Brief behavioural interventions	Primary care	Government	No
Sullivan, 2011 [57]	DMA	Unhealthy alcohol drinkers	Brief counselling interventions (provided by non-physicians)	Primary care setting	Research Institute	No
Wilk, 1997 [58]	DMA	Alcohol abuse, dependence or heavy drinking; aged 19 to 65 years and older	Brief intervention	Primary care and hospital	NR	No
Yuvaraj, 2019 [59]	DMA	Current alcohol drinkers; adults aged more than 18 years, in employment	Screening and brief intervention	Workplace intervention	NR	No
Psychosocial interventions						
Foxcroft, 2016 [60]	DMA	Identified as higher risk; young people aged up to 25 years old	Motivational interviewing	NR	University	Yes
Ghosh, 2021 [61]	DMA	Non-dependent, hazardous alcohol use	Brief intervention/ Motivational interview	LMIC	NR	No
Henssler, 2021 [62]	DMA	Alcohol dependence or alcohol abuse/harmful use; adult	Non-abstinent treatment strategies	Community-based, out-patient, in-patient	Government	No
Hunter, 2019 [63]	DMA	Alcohol dependent; all age groups	Social network interventions	Community-based	Government	No
Klimas, 2018 [64]	DMA	Problem alcohol use; people who use illicit drugs aged over 18 years, attending a range of services (community, inpatient or residential, including opioid agonist treatment)	Psychosocial interventions	NR	Government	No
Lundahl, 2013 [65]	DMA	NR; patients consulting for general medical conditions	Motivational interviewing	Medical care setting such as hospital, physician clinic, emergency department, medically-guided weight loss or diabetes centre, dentist office, or physical therapy office	NR	No
Malaguti, 2020 [66]	DMA	General population (no restrictions)	Forming implementation intentions	NR	None	NR
Mellentin, 2017 [67]	DMA	Adult participants (≥ 18) diagnosed with sub-clinical or clinical AUD	Cue Exposure Therapy; OR Cue Exposure Therapy and Coping Skills Training	NR	Foundation and university	No
Sayegh, 2017 [68]	DMA	NR	Contingency management and motivational interviewing	NR	NR	No
Thomas, 2013 [69]	DMA	Alcohol or drug use; children or adolescent	Mentoring interventions	NR	Government	No
Pharmacological interventions						
Agabio, 2018 [70]	DMA	Alcohol dependence; patients with depression	Antidepressants	Outpatient or inpatient setting	University	No
Bschor, 2018 [71]	DMA	Alcohol dependence, abuse or use disorder	Baclofen	NR	None	No
Carmen, 2004 [72]	DMA	Alcohol dependence	Naltrexone and acamprosate	Ambulatory setting and support groups	NR	No
Cheng, 2020b [73]	DMA	Alcohol dependence or AUD; NR	Gabapentin	NR	NR	Yes
Donoghue, 2017 [74]	DMA	Alcohol dependence, harmful alcohol use, or alcohol abuse; adults (aged ≥ 18 years)	Acamprosate and naltrexone in the treatment of alcohol dependence	In-patient/out-patient	University, government	No
Ipser, 2015 [75]	DMA	Alcohol use disorder	Pharmacological interventions to treat addiction	Outpatient or inpatient setting	Government	No
Li, 2020 [76]	DMA, NMA	AUD; adults with co-morbid depression or depressive symptoms	Pharmacological treatments	NR	Government	No
Jonas, 2014 [77]	DMA	Adults with AUDs	Medications for treating AUD	Outpatient setting	Government	No
Jorgensen, 2011 [78]	DMA	Diagnosis with AUD	Disulfiram	Inpatient and outpatient	NR	No
Kishi, 2013 [79]	DMA	Alcohol dependence	Antipsychotics	NR	No	No
Kranzler, 2019 [80]	DMA	Adults with alcohol dependence or AUD (aged > = 18 years)	Gabapentin	NR	Research and clinical network	Yes
Leone, 2010 [81]	DMA	Alcohol dependent patients receiving therapy to prevent or to treat alcohol withdrawal symptom (AWS)	GHB	Outpatient or inpatient settings	Research centre	No
Lesouef, 2014 [82]	DMA	Alcohol-dependent patients	Baclofen	NR	NR	No
Mann, 2004 [83]	DMA	Alcohol dependence	Acamprosate	NR	NR	No
Mason, 2012 [84]	DMA	Alcohol dependence	Acamprosate treatment of alcohol dependence	NR	NR	Yes
Minozzi, 2018 [85]	DMA	Alcohol use disorder according to DSM-III; adults (aged ≥ 18 years)	Baclofen	Outpatient setting	Government	Yes
Murphy, 2021 [86]	DMA	Alcohol use disorder; NR	Extended-release naltrexone (XR-naltrexone)	Alcohol clinic	None	Yes
Oon-Arom, 2019 [87]	DMA	Patients people with problematic alcohol use	Pharmacological interventions to treat addiction	In-or out-patient setting in any country	University	Yes
Palpacuer, 2015 [88]	DMA	Non-abstinent alcohol dependence; 18 years and over	Nalmefene	NR	University	Yes
Palpacuer, 2018 [89]	DMA, NMA	Alcohol dependence or AUD; non-abstinent patients	Pharmacological interventions to treat addiction	NR	Hospital	No
Pani, 2014 [90]	DMA	Alcohol dependence diagnosed	Anticonvulsants	Not specified	NR	No
Rose, 2018 [91]	DMA	Alcohol use disorders with heavy drinking, craving	Baclofen	NR	NR	No
Rösner, 2010a [92]	DMA	Alcohol dependence; adults aged 18 years and over	Acamprosate	NR	Government	NR
Rösner, 2010b [93]	DMA	Alcohol dependence	Opioid antagonists	NR	Government	NR
Skinner, 2014 [94]	DMA	Diagnosed with alcohol abuse or dependence; adolescent and adult	Disulfiram	NR	None	Yes
Snyder, 2008 [95]	DMA	Adults with alcohol dependence	Acamprosate and naltrexone	Ambulatory setting	NR	NR
Stokes, 2020 [96]	DMA	Substance abuse, dependence, or use disorder; diagnosis of bipolar or major depressive disorder, 18 years and older	Pharmacological treatments	NR	None	No
Streeton, 2001 [97]	DMA	Patients with alcohol dependence or abuse (aged > = 18 years)	Naltrexone	Inpatient and outpatient settings	NR	NR
Vanderkam, 2020 [98]	DMA	AUD; adult	Alpha-blocker	NR	No	No
Miscellaneous interventions						
Apodaca, 2003 [99]	DMA	Problem drinker	Bibliotherapy	Health professional	Government	NR
Thompson, 2020 [100]	DMA	AUD	Physical activity	NR	Government	Yes
Turnbull, 2012 [101]	DMA	Pregnant women with alcohol problems	Home visit during pregnancy	Home-based	NR	No
Multiple interventions						
Carey, 2012 [102]	DMA	All drinking behaviour; college or university students	Computer-delivered interventions and face-to-face interventions	College and university settings	Government	No
Cheng, 2020a [103]	DMA, NMA	Alcohol dependence or AUD; NR	Interventions in recently detoxified, alcohol dependent patients	Primary care setting	Government	No
Davis, 2017 [104]	DMA	NR; emerging adults aged 18–25 years (excludes college students)	Potential moderators of prevention and treatment among emerging adults	Not-for-profit, hospital, emergency department	NR	NR
Dinh-Zarr, 2004 [105]	DMA	Diagnosed with alcohol dependence, alcohol abuse, or hazardous use of alcohol, all of which are’problem drinking’	Interventions for problem drinking	The clinical setting	NR	NR
Egholm, 2018 [106]	DMA	Risky drinking; undergoing all types of surgical procedures under general anaesthesia, regional anaesthesia, or sedation, aged 18 years and over	Perioperative alcohol cessation interventions	Surgical departments (elective and acute) in Copenhagen, Denmark	NR	Yes
Gao, 2018 [107]	DMA, NMA	AUD; NR	Alcohol interventions	NR	NR	No
Hai, 2019 [108]	DMA	Any level of drinking behaviour; women of childbearing age (18 to 45 years old)	Technology-based interventions (TBIs)- Website, text messages, and tablet	Internet, prenatal clinic, hospitals	NR	No
Jarosz, 2013 [109]	DMA	Alcohol-dependent patients	Naltrexone as an adjunct therapy to psychotherapy	NR	Private (pharmaceutical company)	No
Riper, 2011 [110]	DMA	People with alcohol use disorder (exclude students)	Internet-based alcohol interventions (iAIs)	Workplace, community, hospital settings	World Health Organization (WHO)	No
Riper, 2014 [111]	DMA	Exceeded local guidelines for low-risk drinking; adults aged 18 or older	A low-intensity self-help intervention that the participant could perform on a computer or mobile phone, with or without guidance from a professional	Computer or mobile phone	NR	No
Riper, 2018 [112]	SR, DMA	Regular drinker and problem drinkers (exclude students and pregnant women)	Internet-based interventions	Workplace, community, hospital settings (with internet)	None	Yes
Rooke, 2010 [113]	DMA	All drinking behaviour	Computer-delivered brief interventions	Home and research setting	NR	No
Ujhelyi-Gomez, 2021 [114]	DMA	Alcohol use (casual or dependent); pregnant women and women children ≤ 18 years	‘Mocktails’–recipe booklet of non-alcoholic beverages; single session MI; computer-delivered screening and BI; Cognitive behavioural self-help intervention	NR	Government	No
Van Ginneken, 2021 [115]	DMA	Disorders associated with substance abuse; children (aged < 18 years) and adults with mental disorders or distress seeking first-level care/primary care or detected in the community in LMICs	Mental health treatments delivered by trained PWs [Primary-level workers]	LMICs; intervention delivered by primary-level workers (PWs), including primary healthcare professionals (PHPs), lay health workers (people living at the community level with no prior health professional training); and community professionals (e.g. social workers, teachers, development workers)	Government	No

*AUD* Alcohol use disorder, *DMA* Direct meta-analysis, *NMA* Network meta-analysis, *NR* Not reported, *SR* Systematic review without meta-analysis

Almost all studies were direct meta-analyses, with only 6 studies (7%) using network meta-analysis. Most reviews were on brief interventions (30 studies, 35%), followed by pharmacological interventions (29 studies, 34%), multiple interventions (14 studies, 16%), and psychosocial interventions (10 studies, 12%). There was additionally one study on each of the following: physical activity [100], bibliotherapy [99], and home visits during pregnancy [101]. A third of studies did not specify setting of the review. General practice or primary healthcare settings were the most common, at 14 studies (16%), followed by educational institutions (8 studies, 9%) and emergency departments (7 studies, 8%).

### Quality assessment

**Fig. 2:**
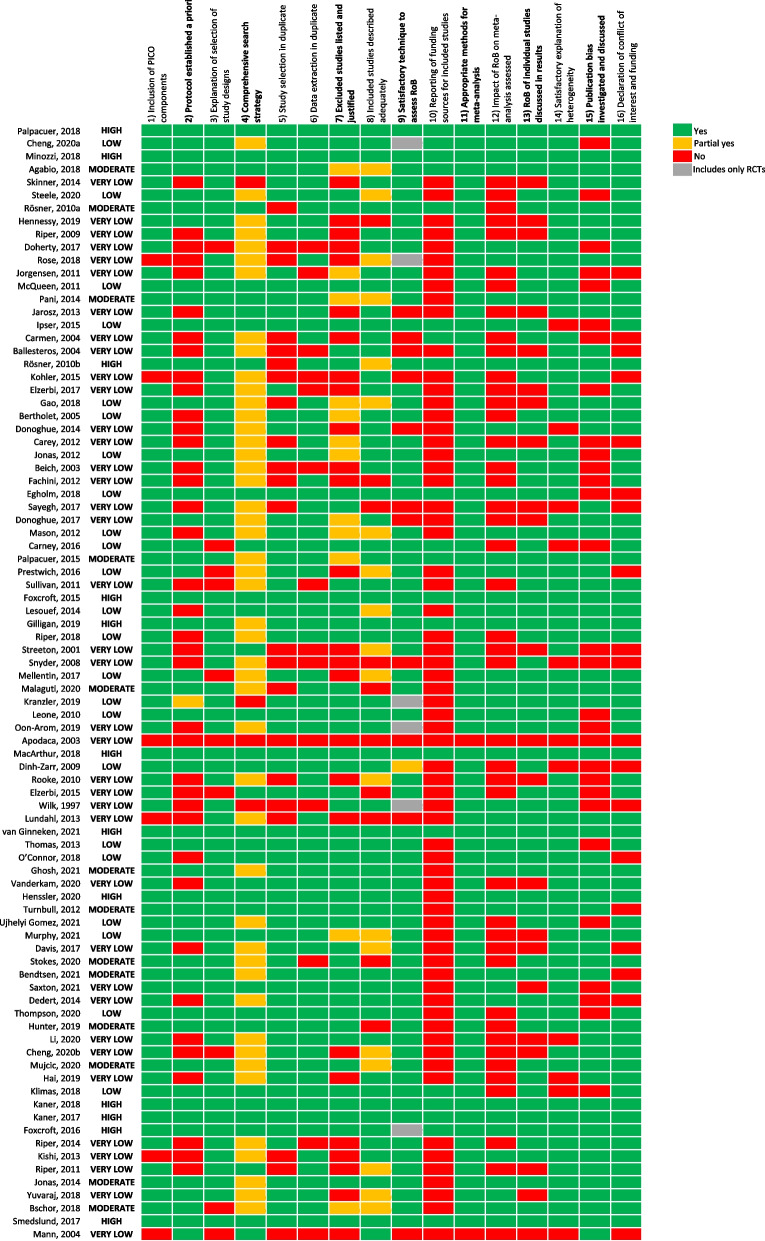
Quality assessment of included studies (AMSTAR2 rating)

The average AMSTAR rating of studies was low (Fig. 2). Among the 11 studies published before 2009, all but two were rated as very low quality. More than 80% studies were assessed as having included PICO components (population, intervention, comparator, outcome), explained the selection of study designs, conducted data extraction in duplicate, selected appropriate methods for meta-analysis, and provided a satisfactory explanation of heterogeneity. However, 66 of 86 studies (77%) did not report funding sources of included studies. Less than half of studies reported a comprehensive search strategy or assessed the impact of risk of bias on the meta-analysis.

### Screening, brief intervention, and referral to treatment (SBIRT) outcomes

From the review, 29 studies were identified for screening, brief intervention and referral to treatment and 1 network meta-analysis considered the impact of brief intervention combined with other interventions (Tables 3 and 4).

**Table 3:** Summary results of included systematic reviews, for screening, brief intervention, and referral to treatment (SBIRT)

**Intervention**	**Population**	**Comparator**	**Outcome**	**Timeframe**	**Effect size** (method: effect (95% CI))	**I**^**2**^	**Publication bias**	**Number of RCTs**	**Quality assessment of RCTs**
**Minimal intervention**									
Minimal intervention: a unique session of general advice on alcohol consumption lasting ~ 3–5 min but without stressing strategies to decrease consumption [31]	Hazardous drinker	Control (no specific advice on alcohol consumption to participants from their primary care providers)	Decrease in the frequency of hazardous drinkers	6–12 months	OR: 0.95 (0.72, 1.25)	NR	NR	3	NR
**Brief intervention**									
Screening in general practice for excessive alcohol use and providing brief interventions [30]	Excessive alcohol use	No/less intervention	Heavy drinking: Absolute risk reduction (%)	6 months to 4 years	Risk reduction: 10.5% (7.1%, 13.9%)	NR	NR	8	NR
			Heavy drinking: Number needed to treat (NNT)	6 months to 4 years	Number needed to treat: 10 (7, 14)	NR	NR	8	NR
Brief intervention (~ 10–15 min in one session concerning alcohol consumption, health risks, and strategies to decrease alcohol intake, with possible simple reinforcing visits through follow-up of ~ 3–5 min each [31]	Hazardous drinker	Control (no specific advice on alcohol consumption to participants from their primary care providers)	Decrease in the frequency of hazardous drinkers	6–18 months	OR: 1.6 (1.33, 1.93)	NR	NR	9	NR
	Hazardous drinker, non-treatment seeker	Control (no specific advice on alcohol consumption to participants from their primary care providers)	Decrease in the frequency of hazardous drinkers	6–12 months	OR: 2.19 (1.68, 2.84)	20.5%	NR	3	NR
	Hazardous drinker, treatment seeker	Control (no specific advice on alcohol consumption to participants from their primary care providers)	Decrease in the frequency of hazardous drinkers	6–12 months	OR: 1.41 (1.20, 1.65)	9.4%	NR	9	NR
	Heavy drinker	Control (no specific advice on alcohol consumption to participants from their primary care providers)	Decrease in the frequency of hazardous drinkers	6–12 months	OR: 1.94 (1.55, 2.43)	37.6%	NR	NR	NR
	Moderate drinker	Control (no specific advice on alcohol consumption to participants from their primary care providers)	Decrease in the frequency of hazardous drinkers	6–12 months	OR: 1.42 (1.19, 1.68)	22.5%	NR	NR	NR
Brief intervention: delivered individually, focused on alcohol consumption with a face-to-face component during the initial session, and “brief intervention” or “motivational intervention” or reporting the use of feedback or advice to reduce alcohol consumption [33]	Risky drinkers attending primary care facilities but not seeking help for alcohol-related problems	Usual care without explicit mention of advice regarding alcohol use or no intervention	Alcohol consumption: net change of ethanol	6 to 12 months	OR: –37.87 (–51.13, –24.61)	25.8%	No	12	Moderate: the quality score ranked from 5–18 from total score of 18
Face-to-face brief intervention [102]	College or university students	No-treatment control	Alcohol consumption: Quantity per week/month	≤ 13 to ≥ 27 weeks	SMD: 0.19 (0.11, 0.27)	0%	NR	21	Moderate
			Frequency of heavy drinking days	≤ 13 weeks	MD: 0.16 (0.07, 0.25)	0%	NR	21	Moderate
BI delivered face-to-face [34]	Adolescents attending high school, secondary school, or a further education training college; used alcohol or other drugs, not dependent, but faced negative behavioural consequences due to substance use	Control (no intervention, placebo, assessment only, or other types of interventions or education)	Alcohol consumption frequency	Short term (1–3 months)	SMD: 0.02 (-0.22, 0.26)	NR	NR	2	NR
				Intermediate term (4–6 months)	SMD: -0.14 (-0.33 to 0.05)	0%	NR	2	NR
			Heavy drinking	3—12 months	SMD: -0.01 (-0.20, 0.18)	0%	NR	6	NR
Brief intervention [105]	Diagnosed with alcohol dependence, alcohol abuse, or hazardous use of alcohol	No intervention	Injury-Related Deaths	16 weeks to 16 years	RR: 0.65 (0.21, 2.00)	0.0%	Yes	3	NR
Brief alcohol intervention [36]	Military conscripts	Control	Self-reported alcohol consumption	6 – 20 months	WMD: 0.17 (-0.59, 0.93)	0%	NR	4	Mean quality rating score: 20 out of 32
	Military serving	Control	Self-reported alcohol consumption	6 – 20 months	WMD: 0.68 (-0.64, 1.99)	0%	NR	2	
	Veterans	Control	Self-reported alcohol consumption	6 – 20 months	WMD: 2.39 (-1.51, 6.29)	0%	NR	4	
BI face to face [36]	Military and veterans	Control	Self-reported alcohol consumption	6 – 20 months	WMD: 0.16 (-0.88, 1.20)	0%	NR	3	
BI in **primary healthcare** (opportunistic screening and early intervention, no more than four sessions, each session lasting no longer than 30 min, delivered by non-specialist personnel) [38]	Non-treatment-seeking, hazardous or harmful drinking	Control (screening only, assessment only, treatment as usual, evaluation only, minimal intervention)	Quantity of alcohol consumed per week	6 months	MD (g/week): -21.98 (-37.40, -6.57)	24%	NR	10	NR
				12 months	MD (g/week): -30.86 (-46.49, -15.23)	65%	NR	16	NR
BI in **emergency departments** (opportunistic screening and early intervention, no more than four sessions, each session lasting no longer than 30 min, delivered by non-specialist personnel) [38]	Non-treatment-seeking, hazardous or harmful drinking	Control (screening only, assessment only, treatment as usual, evaluation only, minimal intervention)	Quantity of alcohol consumed per week	6 months	MD (g/week): -17.97 (-29.69, -6.24)	0%	NR	4	NR
				12 months	MD (g/week): -18.21 (-26.71, -9.70)	28%	NR	8	NR
Brief intervention (targeted injury studies) after opportunistic screening at emergency department [39]	Non-treatment seeking, hazardous or harmful drinkers	Control: from “screening only” and “assessment only” to “treatment as usual,” “evaluation only,” or “minimal intervention” (such as the provision of an information leaflet)	Reduction in final quantity of alcohol consumed	up to 5 months	SMD: -0.14 (-0.30, 0.03)	29%	Yes	4	High quality
				6 months	SMD: -0.10 (- 0.17, -0.02)	0%	Yes	4	High quality
				12 months	SMD: -0.04 (-0.11, 0.03)	0%	Yes	8	High quality
Brief intervention (non-injury-specific trials) after opportunistic screening at emergency department [39]	Non-treatment seeking, hazardous or harmful drinking	Control: from “screening only” and “assessment only” to “treatment as usual,” “evaluation only,” or “minimal intervention” (such as the provision of an information leaflet)	Reduction in final quantity of alcohol consumed	up to 5 months	SMD: -0.15 (-0.24, -0.07)	0%	Yes	NR	High quality
				6 months	SMD: -0.06 (-0.13, 0.02)	1%	Yes	NR	High quality
				12 months	SMD: -0.08 ( -0.15, -0.01)	0%	Yes	NR	High quality
Brief Alcohol Screening Intervention for College Students (BASICS): delivered face-to-face and usually conducted over two structured sessions, including motivational interview and personalized feedback based on drinking behaviour [40]	College students	Control intervention	Alcohol consumption: Self-reported drinks per week	12 months	MD: − 1.50 (-3.24, − 0.29)	NR	NR	12	Moderate
Brief intervention [107]	AUD	CM	Abstinent rate, during treatment	≥ 12 weeks	OR: 2.75 (0.31, 31.82)	67.3%	Yes	60	NR
			Abstinent rate, after treatment	≥ 12 weeks	OR: 1.47 (0.24, 10.96)	67.3%	Yes	60	NR
		CM + psychotherapy	Abstinent rate, during treatment	≥ 12 weeks	OR: 4.06 (2.07, 8.18)	67.3%	Yes	60	NR
			Abstinent rate, after treatment	≥ 12 weeks	OR: 0.81 (0.42, 1.52)	67.3%	Yes	60	NR
		Control	Abstinent rate, during treatment	≥ 12 weeks	OR: 0.81 (0.59, 1.12)	67.3%	Yes	60	NR
			Abstinent rate, after treatment	≥ 12 weeks	OR: 0.76 (0.46, 1.22)	67.3%	Yes	60	NR
		Pharmacotherapy	Abstinent rate, during treatment	≥ 12 weeks	OR: 0.96 (0.64, 1.48)	67.3%	Yes	60	NR
			Abstinent rate, after treatment	≥ 12 weeks	OR: 0.52 (0.26, 1.02)	67.3%	Yes	60	NR
		Pharmacotherapy + BI	Abstinent rate, during treatment	≥ 12 weeks	OR: 1.87 (1.40, 2.49)	67.3%	Yes	60	NR
			Abstinent rate, after treatment	≥ 12 weeks	OR: 0.53 (0.39, 0.74)	67.3%	Yes	60	NR
		Pharmacotherapy + psychotherapy	Abstinent rate, during treatment	≥ 12 weeks	OR: 1.43 (0.96, 2.22)	67.3%	Yes	60	NR
			Abstinent rate, after treatment	≥ 12 weeks	OR: 0.48 (0.27, 0.84)	67.3%	Yes	60	NR
		Psychotherapy	Abstinent rate, during treatment	≥ 12 weeks	OR: 1.23 (0.87, 1.76)	67.3%	Yes	60	NR
			Abstinent rate, after treatment	≥ 12 weeks	OR: 0.75 (0.52, 1.06)	67.3%	Yes	60	NR
		Psychotherapy + BI	Abstinent rate, during treatment	≥ 12 weeks	OR: 1.48 (0.79, 2.78)	67.3%	Yes	60	NR
			Abstinent rate, after treatment	≥ 12 weeks	OR: 1.65 (0.71, 3.92)	67.3%	Yes	60	NR
Brief intervention -conversation comprising five or fewer session of brief advice or brief lifestyle counselling and a total duration of less than 60 min [46]	Hazardous or harmful alcohol consumption as identified by a screening tool	Control group—screening or assessment only	Quantity of drinking (g/week)	12 months	MD: -20.08 (-28.36, -11.81)	73%	Yes	34	Moderate
			Frequency of binge drinking (binges/week)	12 months	MD: -0.08 (-0.14, -0.02)	22%	NR	15	Moderate
			Frequency of drinking (days drinking/week)	12 months	MD: -0.13 (-0.23, -0.04)	0%	NR	11	Moderate
			Intensity of drinking (g/drinking day)	12 months	MD: -0.18 (-3.09, 2.73)	25%	NR	10	Moderate
			Heavy drinkers	12 months	MD: -0.09 (-0.13, -0.04)	77%	NR	18	NR
			Binge drinkers	12 months	Risk difference: -0.07 (-0.12, -0.02)	76%	NR	10	NR
			Laboratory markers—GGT (IU/L)	12 months	MD: -0.89 (-3.86, 2.08)	0.0%	NR	3	NR
Brief intervention: as a single session or up to three sessions involving an individual patient and health care practitioner comprising information and advice, often using counselling type skills to encourage a reduction in alcohol consumption and related problems [49]	Heavy alcohol users admitted to general hospital inpatient units	Assessment only (screening) or treatment as usual including provision of leaflets	Self-reported alcohol consumption in grams per week	6 months	WMD: -69.43 (-128.14, -10.72)	68%	NR	8	Six studies high risk of selection bias; six studies inadequate allocation concealment; outcome assessors blinded in 11 studies
				9 months	WMD: -182.88 (-360.00, -5.76)				
				1 year	WMD: -33.62 (-82.27, 15.03)				
			Mean alcohol consumption per week	6 months	SMD: -0.26 (-0.73, 0.21)	79%	NR	3	
				1 year	SMD: -0.08 (-0.41, 2.4)				
			Laboratory markers (GammaGT)	6 months	WMD: 7.00 (-33.77, 47.77)	0%	NR	3	
				1 year	WMD: -5.05 (-36.82, 26.73)				
Assessment and feedback [55]	At risk drinking	No intervention	Alcohol consumption	0–6 months	SMD: -0.17 (-0.27, -0.08)	52%	Yes	15	Low
				> 6 months	SMD: -0.17 (-0.30, -0.04)	0%	NR	3	Low
		Assessment only	Alcohol consumption	0–6 months	SMD: -0.15 (-0.25, -0.06)	64%	No	23	Low
				> 6 months	SMD: -0.03 (-0.19, 0.12)	0%	NR	3	Very low
		Education	Alcohol consumption	0–6 months	SMD: -0.02 (-0.21, 0.17)	52%	NR	7	Very low
Feedback plus moderation skills [55]	At risk drinking	Feedback only	Blood alcohol content	0–6 months	SMD: -0.26 (-0.49, -0.03)	0%	NR	2	Low
Primary health professional- and community professional-led interventions (single brief intervention) [115]	Adult patients with harmful or hazardous alcohol use	Enhanced usual care	AUDIT/ASSIST score	1 to 6 months	RR: 0.93 (0.77, 1.12)	28%	NA (< 4 studies)	3	Low/unclear risk
				1 to 6 months	SMD: -0.15 (-0.27, -0.03)	0%	NA (< 4 studies)	3	Low/unclear risk
				> 6 months	RR: 0.88 (0.73, 1.06)	4%	NA (< 4 studies)	2	Low/unclear risk
				> 6 months	SMD: 0.12 (-0.32, 0.55)	83%	NA (< 4 studies)	2	Low/unclear risk
**Extended brief intervention**									
Extended brief interventions (characteristics of brief intervention but also included several specific reinforcement sessions through follow-up, ~ 10–15 min each) [31]	Hazardous drinker	control (no specific advice on alcohol consumption to participants from their primary care providers)	Decrease in the frequency of hazardous drinkers	6–12 months	OR: 1.5 (1.12, 1.95)	NR	NR	3	NR
Brief intervention + extended brief intervention (all studies combined) [31]	Hazardous drinker	control or minimal intervention	Decrease in the frequency of hazardous drinkers	6–12 months	OR: 1.55 (1.27, 1.90)	19.24%	NR	12	NR
Extended intervention—more than five sessions or total combined session durations was more than 60 min [46]	Hazardous or harmful alcohol consumption, as identified by a screening tool	Minimal or no intervention—screening or assessment only, usual care for the presenting condition or written information such as education leaflet	Quantity of drinking (g/week)	12 months	MD: -14.43 ( -37.41, 8.54)	41%	NR	6	Moderate
			Frequency of drinking (days drinking/week)	12 months	MD: -0.45 ( -0.81, -0.09)	0.0%	NR	2	Moderate
			Frequency of binge drinking (binges/week)	12 months	MD: -0.08 ( -0.28, 0.12)	0.0%	NR	2	NR
			Binge drinkers	12 months	Risk difference: -0.02 ( -0.07, 0.03)	0.0%	NR	2	NR
		Brief intervention	Quantity of drinking (g/week)	12 months	MD: 1.54 (-42.01, 45.10)	0.0%	NR	3	Low
			Binge drinkers	12 months	Risk difference: 0.02 (-0.08, 0.12)	0.0%	NR	2	NR
Multi-dose assessment and feedback [55]	At risk drinking	Single-dose assessment and feedback	Alcohol consumption	0–6 months	RR: 0.84 (0.78, 0.91)	38%	NR	4	Moderate
**Brief counselling interventions**									
Behavioural counselling after screening [44]	Adults with risky drinking	Control	Change in alcohol consumption from baseline to 12 months (drinks/week)	12 months	MD: -3.573 (-4.758, -2.389)	13.7%	NR	12	NR
			Heavy drinking: No heavy drinking episodes at 12 months	12 months	Risk difference: 0.118 (0.074, 0.162)	17.2%	NR	7	NR
Screening, counselling intervention and referral [51]	Non dependent alcohol user; adolescents and adult	No intervention	Total alcohol consumption (drink/week)	NR	MD: -1.59 (-2.15, -1.03)	63%	Yes	32	Good to fair
	Non dependent alcohol user; young adults	No intervention	Total alcohol consumption (drink/week)	NR	MD: -0.86 (-1.29, -0.43)	11%	NR	14	NR
	Non dependent alcohol user; general adult	No intervention	Total alcohol consumption (drink/week)	NR	MD: -2.51 (-3.81, -1.21)	70%	NR	15	NR
	Non dependent alcohol user; older adult	No intervention	Total alcohol consumption (drink/week)	NR	MD: -2.98 (-6.96, 0.99)	81%	NR	2	NR
Brief counselling interventions (provided by nonphysicians) [57]	Unhealthy alcohol drinkers	Control (no structured alcohol intervention)	Alcohol consumption (self-reported drink per week)	6 to 12 months	MD: 1.73 (0.03, 3.5)	46.8%	No	7	Moderate (4 studies = fair; 3 = poor)
Lay health worker-led interventions (counselling, motivational brief intervention, brief intervention) [115]	Adult patients with harmful or hazardous alcohol use	Enhanced usual care	ASSIST/AUDIT score	1 to 6 months	SMD: -0.22 (-0.32, -0.11)	0%	NA (< 4 studies)	3	1 study high risk blinding outcomes
**Brief motivational interview**									
Brief motivational interventions [47]	Young people with existing alcohol use problems	Control interventions, or standard care	Self-reported the frequency of drinking days/ AUDIT-C cut-off score	3 – 12 months	SMD: − 0.17 (− 0.32, − 0.02)	42%	NR	6	The quality of the six RCTs included was poor to good
Brief motivational interventions—feedback and education in the harm of heavy drinking and advice to moderate drinking to low-risk, problem-free levels [58]	Heavy drinkers	No intervention	Drinking moderation	6 months—12 months	OR: 1.95 (1.66, 2.30)	NR	NR	8	Mostly equivalent to published rates
Brief intervention/MI [61]	Non-dependent, harmful or hazardous alcohol use	Waitlist, usual care	Risk-scores in standard screening instruments	3 months	SMD: − 0.34 (− 0.67, − 0.01)	90%	No	7	Moderate
				6 months	SMD: − 0.06, (− 0.32, 0.21)	70%	NR	3	NR
				12 months	SMD: 0.15 (− 0.21, 0.52)	NR	NR	2	NR
			Change in % persons with heavy drinking	3 months	OR: 0.87 (0.61,1.25)	0%	NR	2	NR
			Change in proportion of the frequency of heavy drinking	6 months	SMD: 0.03 (-0.18,0.25)	0%	NR	2	NR
				12 months	SMD: 0.03 (-0.16,0.22)	0%	NR	2	NR
			Transition from high risk to low risk alcohol use (AUDIT scores)	3 months	OR: 1.2 (0.86,1.68)	74%	NR	3	NR
				6 months	OR: 0.97 (0.64,1.49)	56%	NR	4	NR
				12 months	OR: 1.44 (0.73,2.86)	84%	NR	3	NR
**Social norms interventions**									
Social norms interventions (all delivery modes) [41]	University and college students	No intervention (assessment only or alcohol information or alternative (non-normative) intervention)	Mean number of drinks per week	4 + months	SMD: -0.08 (-0.12, -0.05)	13%	Suspected	32	Moderate
			Mean peak Blood alcohol concentration (BAC)	4 + months	SMD: -0.08 (-0.17, 0.00)	50%	Suspected	11	Low
Social norms interventions delivered via individual face to-face feedback [41]	University and college students	No intervention (assessment only or alcohol information or alternative (non-normative) intervention)	Number of drinking days per week	4 + months	SMD: -0.21 (-0.31, -0.10)	0%	Suspected	8	Moderate
			Mean peak Blood alcohol concentration (BAC)	4 + months	SMD: -0.08 (-0.26, 0.10)	0%	Suspected	4	Moderate
Social norms interventions delivered via GROUP face to-face feedback [41]	University and college students	No intervention (assessment only or alcohol information or alternative (non-normative) intervention)	Number of drinking days per week	4 + months	SMD: -0.26 (-0.54, 0.02)	55%	Suspected	5	Low
Social influence interventions [52]	College or university students	Combined multiple comparators (with no social influences)	Combined outcomes of alcohol consumption	NR	SMD: 0.29 (0.22, 0.37)	83.8%	Yes	41	Moderate
**Other brief interventions**									
‘Mocktails’–recipe booklet of non-alcoholic beverages; single session MI; computer-delivered screening and BI; cognitive behavioural self-help intervention [114]	Pregnant women with alcohol problem	Usual care	Abstinence	NR	OR: 2.31 (1.61,3.32)	0%	NR	3	Low
	Motherhood with alcohol problem	Usual care	Alcohol consumption	NR	SMD: 0.20 (0.38, 0.02)	0%	NR	4	Low
Targeted individual-level multiple risk behaviour interventions [48]	Children and young people aged 0–18 years	No intervention/ usual practice	Combined measure of alcohol consumption	12 months	OR: 1.02 (0.80,1.31)	48.02%	NR	4	Moderate
			Combined measure of binge drinking	12 months	OR: 0.97 (0.6, 1.37)	0%	NR	3	NR
Targeted family-level multiple risk behaviour interventions [48]	Children and young people aged 0–18 years	No intervention/ usual practice	Combined measure of alcohol consumption	12 months	OR: 0.83 (0.47, 1.46)	29.14%	NR	3	Moderate
				Over 12 months	OR: 1.24 (0.69, 2.24)	70.02%	NR	3	NR
Targeted school-level multiple risk behaviour interventions [48]	Children and young people aged 0–18 years	No intervention/ usual practice	Combined measure of alcohol consumption	Over 12 months	OR: 0.73 (0.52, 1.03)	0%	NR	2	NR
Lifestyle campaign over four weeks at workplace: "Group sessions and web-based follow-up, pen and paper based mental simulation manipulation exercise" [59]	Adults aged more than 18 years in employment, current alcohol drinkers	Usual care	Alcohol consumption	NR	MD: − 2.25 (− 4.20, − 0.30)	0%	No	NR	Moderate (high risk blinding of participants and outcome assessment)
**Combination interventions**									
Pharmacotherapy + BI [107]	AUD	Control	Abstinent rate, during treatment	≥ 12 weeks	OR: 2.30 (1.51, 3.51)	67.3%	NR	60	NR
			Abstinent rate, after treatment	≥ 12 weeks	OR: 0.70 (0.40, 1.26)	67.3%	NR	60	NR
Phamacotherapy + BI [107]	AUD	Pharmacotherapy + psychotherapy	Abstinent rate, during treatment	≥ 12 weeks	OR: 0.76 (0.47, 1.28)	67.3%	NR	60	NR
			Abstinent rate, after treatment	≥ 12 weeks	OR: 0.91 (0.50, 1.63)	67.3%	NR	60	NR
Phamacotherapy + BI [107]	AUD	Psychotherapy	Abstinent rate, during treatment	≥ 12 weeks	OR: 0.66 (0.42, 1.03)	67.3%	NR	60	NR
			Abstinent rate, after treatment	≥ 12 weeks	OR: 1.41 (0.90, 2.20)	67.3%	NR	60	NR
Phamacotherapy + BI [107]	AUD	Psychotherapy + BI	Abstinent rate, during treatment	≥ 12 weeks	OR: 0.79 (0.40, 1.58)	67.3%	NR	60	NR
			Abstinent rate, after treatment	≥ 12 weeks	OR: 3.13 (1.26, 7.74)	67.3%	NR	60	NR
Pharmacotherapy [107]	AUD	Pharmacotherapy + BI	Abstinent rate, during treatment	≥ 12 weeks	OR: 1.95 (1.18, 3.18)	67.3%	NR	60	NR
			Abstinent rate, after treatment	≥ 12 weeks	OR: 1.02 (0.50, 2.14)	67.3%	NR	60	NR
Pharmacotherapy [107]	AUD	Pharmacotherapy + BI	Abstinent rate, during treatment	≥ 12 weeks	OR: 1.02 (0.50, 2.14)	67.3%	NR	60	NR
			Abstinent rate, after treatment	≥ 12 weeks	OR: 1.54 (0.72, 3.28)	67.3%	NR	60	NR
Pharmacotherapy [107]	AUD	Psychotherapy + BI	Abstinent rate, during treatment	≥ 12 weeks	OR: 3.20 (1.08, 9.55)	67.3%	NR	60	NR
			Abstinent rate, after treatment	≥ 12 weeks	OR: 1.02 (0.50, 2.14)	67.3%	NR	60	NR
Pharmacotherapy [107]	AUD	Psychotherapy + BI	Abstinent rate, during treatment	≥ 12 weeks	OR: 1.54 (0.72, 3.28)	67.3%	NR	60	NR
			Abstinent rate, after treatment	≥ 12 weeks	OR: 3.20 (1.08, 9.55)	67.3%	NR	60	NR
Pharmacotherapy + Psychotherapy [107]	AUD	Psychotherapy + BI	Abstinent rate, during treatment	≥ 12 weeks	OR: 1.03 (0.48, 2.19)	67.3%	NR	60	NR
			Abstinent rate, after treatment	≥ 12 weeks	OR: 3.47 (1.26, 9.52)	67.3%	NR	60	NR
Psychotherapy + BI [107]	AUD	Control	Abstinent rate, during treatment	≥ 12 weeks	OR: 1.82 (0.90, 3.71)	67.3%	NR	60	NR
			Abstinent rate, after treatment	≥ 12 weeks	OR: 2.18 (0.84, 6.01)	67.3%	NR	60	NR

*ASI* Alcohol severity index, *AUD* Alcohol use disorder, *AUDIT* AUD identification test, *BI* Brief intervention, *CI* Confidence interval, *CM* Contingency management, *MD* Mean difference, *MW* Mean weighted effect size, *NMD* Network mean difference, *NR* Not reported, *OR* Odds ratio, *RAPI* Rutgers Alcohol Problems Index, *RCT* Randomized controlled trial, *RR* Relative risk, *SBIRT* Screening, brief intervention and referral to treatment, *SMD* Standard mean difference, *WMD* Weighted mean difference

**Table 4:** Certainty in evidence (GRADE) for screening, brief intervention, and referral to treatment (SBIRT)

		Hazardous/harmful alcohol use				AUD	
		Screening and brief intervention	Brief motivational interviewing	Brief counselling	Social norms	Brief intervention (BI)	BI with another intervention
Risk of bias	Meta-analyses (AMSTAR)	Low	Very low	Very low	Moderate	Very low	Low
	RCTs	Low	Moderate	Moderate	Moderate	NR	NR
Inconsistency	Meta-analyses	Inconsistent (but most showed a significant effect)	Very inconsistent	Mostly consistent	Consistent	Consistent	NA (single study)
	Heterogeneity of RCTs	Very high	Very high	Moderate	Moderate	Moderate	High
Indirectness		Applicable	1/3 studies on young people	1/3 studies on non-physicians	Students only	No consumption outcomes	Applicable
Imprecision	Confidence interval	Variable	Variable	Small	Small	Very wide	Very wide
	Sample size	Thousands	Thousands	Thousands	Thousands	Thousands	Thousands for NMA
Small study effect (number of studies)		Yes (2), No (1), NR (7)	No (1), NR (2)	Yes (1), No (1), NR (1)	Yes (2)	Yes (2)	Yes (1)
Other		Small effect size, long timeframe	NA	NA	Shorter timeframe	NA	NA
**Overall**		**Low**	**Very low**	**Very low**	**Very low**	**Very low**	**Very low**

*AUD* Alcohol use disorder, *NA* Not applicable, *NMA* Network meta-analysis, *NR* Not reported, *RCT* Randomised controlled trial

Eight studies reviewed the efficacy of brief interventions across all hazardous and harmful drinkers [30, 31, 33, 38, 39, 46, 49, 115]. One high quality review of brief interventions totalling up to five sessions with total duration less than 60 min found a small but significant reduction across seven measures of alcohol consumption, heavy drinking, and binge drinking after a period of 12 months [46]. Five studies on interventions provided in primary care or general practice settings consistently found a significant effect beyond 6 months follow-up, across heavy drinking (absolute risk reduction 10.5%, 95% CI 7.1% to 13.9%) [30], frequency of hazardous drinkers (OR 1.6, 95% CI 1.33, 1.93) [31], net change in ethanol consumed (OR -37.87, 95% CI -51.13 to -24.61) [33], and grams of alcohol consumed per week (MD -30.86, 95% CI -46.49 to -15.23, at 12 months [38]. Among the studies on brief interventions delivered following opportunistic screening among non-treatment seeking drinkers in the emergency department, one found a significant reduction in grams of alcohol consumed per week (MD -18.21, 95% CI -26.71 to -9.70 at 12 months), while the other found a very small effect on alcohol consumption up to 5 months (SMD -0.15, 95% CI -0.24, -0.07), at 6 months (SMD -0.10, 95% CI -0.17 to -0.02), and at 12 months follow-up (SMD -0.06, 95% CI -0.13 to 0.02), but not among trials specifically considering injury patients [39]. The single study on heavy users admitted to general hospital inpatient units reported a significant effect on self-reported alcohol consumption at 6 months (WMD -69.43, 95% CI -128.14 to -10.72) and 9 months follow-up, but not at 12 months (WMD -33.62, 95% CI -82.27 to 15.03) and no effect for laboratory markers at either 6 months (WMD 7.00, 95% CI -33.77 to 47.77) or 12 months follow-up (WMD -5.05, 95% CI -36.82 to 26.73) [49]. A study considering a single brief intervention delivered by primary health professionals and community professionals in LMICs found no significant effect on relative risk reduction at 1–6 months (RR 0.93, 95% CI 0.77 to 1.12) or more than 6 months follow-up (RR 0.88, 95% CI 0.73 to 1.06) [115]. Overall confidence in the evidence for brief interventions in hazardous and harmful drinkers was graded as low. Although the sample size in all studies was very large and most studies showed an effect over the mid-long term, methodological quality of RCTs and meta-analyses was low, two reviews identified publication bias (out of three studies reporting on publication bias), and there was high heterogeneity among RCTs in many of the reviews.

Among the studies considering effect on hazardous/harmful drinking in specific sub-populations, a low-quality meta-analysis among concurrent illicit drug users with a small total sample (170 participants) found no effect on alcohol use after 3 months (SMD 0.07, 95% CI -0.24 to 0.37) [64]. A study among military and veterans did not identify a significant effect on self-reported alcohol consumption at 6–20 months, although confidence intervals were very wide (WMD 0.16, 95% CI -0.88 to 1.20, face-to-face delivery) [36]. However, a review encompassing different types of brief interventions delivered to pregnant women and mothers with alcohol problems (including single session motivational interview, computer-delivered screening and brief intervention, and self-help cognitive behavioural therapy) found a significant effect when combining all interventions on abstinence in pregnant women (OR 2.31, 95% CI 1.61 to 3.32) and alcohol consumption among women with dependent children (SMD 0.20, 95% CI 0.02, 0.38) as compared with usual care [114]. Similarly, a review of group sessions and web-based follow-up delivered through the workplace found a significant reduction in alcohol consumption compared with usual care (MD -2.25, 95% CI -4.20 to -0.30) [59].

Six studies considered brief interventions among adolescents, students and young adults [34, 40, 41, 52, 55, 102]. A high quality review of assessment and feedback among young people with at-risk drinking found a small but significant effect on alcohol consumption compared with no intervention at 0–6 months (SMD –0.17, 95% CI –0.27 to –0.08) and more than 6 months (SMD –0.17, 95% CI –0.3- to –0.04), but there was no significant effect when compared with education [55]. The same review found a significant reduction in blood alcohol content at 0–6 months for feedback combined with moderation skills when compared with feedback alone (SMD -0.26, 95% CI -0.49 to -0.03) [55]. Another study of adolescents in school or college facing negative consequences due to their alcohol use did not find a significant effect on alcohol consumption frequency in the short or intermediate term, nor on heavy drinking at 3–12 months follow-up (SMD -0.01, 95% CI -0.20 to 0.18) [34]. Conversely, studies in college/university students found a small but significant effect, for self-reported drinks per week at 12 months follow-up (MD -1.50, 95% CI -3.24 to -0.29) [40], frequency of heavy drinking days after 3 months (MD 0.16, 95% CI 0.07 to 0.25) [102], and alcohol consumption after 3–7 months (SMD 0.19, 95% CI 0.11 to 0.27) [102]. Among the studies of social norms interventions among university and college students, one high quality study found a small but significant effect across all delivery modes at more than 4 months follow-up for mean peak blood alcohol concentration (SMD -0.08, 95% CI -0.17 to 0.00) and drinks per week (SMD -0.08, 95% CI -0.12 to -0.05), although there was suspected publication bias [41]. Sub-group analyses for face-to-face and group feedback were not significant, aside from drinking days per week for face-to-face feedback. A low quality study across all modes of social influence interventions for any length of follow-up found a significant decrease across all outcomes of alcohol consumption (SMD 0.29, 95% CI 0.22 to 0.37) but noted very high heterogeneity and publication bias [52]. The body of evidence for social norms interventions was downgraded to very low due to the presence of publication bias and small effect size.

Regarding length of the intervention, a single study considered minimal intervention, defined as lasting 3–5 min, compared to control in hazardous drinkers and found no significant effect at 6–12 months (OR 0.95, 95% CI 0.72 to 1.25) [31]. Although the study found a significant decrease in the frequency of hazardous drinkers for extended brief interventions after 6–12 months (OR 1.5, 95% CI 1.12 to 1.95), the effect was similar to the combined effect for brief intervention and extended brief intervention studies (OR 1.55, 95% CI 1.27 to 1.90) [31]. It should also be noted that the study defined an extended brief intervention as having reinforcement sessions of 10–15 min, which overlaps with the definition of brief intervention in certain other studies [31, 38–40, 46, 49]. Another study found a very small reduction for extended brief intervention (defined as either more than 5 sessions or combined session durations totalling more than 60 min) in drinking days per week at 12 months follow-up, as compared to minimal or no intervention (MD -0.45, 95% CI -0.81 to -0.09), but no effect for binge drinking or quantity of alcohol consumption, or when compared with brief intervention [46]. A study comparing multi-dose assessment and feedback with single dose assessment and feedback among at-risk young drinkers found a small reduction in alcohol consumption at 0–6 months post-intervention (RR 0.84, 95% CI 0.78 to 0.91) [55].

Among the studies considering a specific type of brief interview, three considered brief motivational interviewing [9, 47, 58], three brief counselling [44, 51], one lay health-worker delivered interventions [115], and one brief interventions targeting multiple risk behaviours simultaneously [48]. All brief counselling interventions found a small but significant effect on drinks per week (MD -3.57, 95% CI -4.76 to -2.39, 12 months [44]; MD -1.59, 95% CI -2.15 to -1.03 [51]; MD 1.73, 95% CI 0.03 to 3.5, 6–12 months [57]) and heavy drinking (risk difference 0.12, 95% CI 0.07 to 0.16, at 12 months) [44]. Certainty in the evidence was graded as very low due to the presence of publication bias and very low AMSTAR rating of meta-analyses. For brief motivational interviews, one study in heavy drinkers found a large improvement in drinking moderation at 6–12 months (OR 1.95, 95% CI 1.66 to 2.30) [58] and a study of motivational interviewing delivered to young people with alcohol problems in emergency care found a significant effect at 3–12 months (SMD -0.17, 95% CI -0.32 to -0.02) [47]. However, a study across LMICs (primarily South Africa and India) found no effect at 3 months, 6 months, or 12 months across four different outcomes, except screening risk score after 3 months (SMD -0.34, 95% CI -0.67 to -0.01) [9]. There was very high heterogeneity for all of the outcomes based on more than 2 RCTs, suggesting significant variation in either context or how the intervention was delivered across RCTs. A review of lay health-worker led interventions in LMICs, including counselling, brief intervention, and brief motivational intervention, did find a significant improvement in screening score at 1 to 6 months (SMD -0.22, 95% CI -0.32 to -0.11) [115]. Certainty in the evidence for brief motivational interviewing was rated as very low due to very low AMSTAR ratings and inconsistency across both the meta-analyses and the RCTs within each meta-analysis. A high quality review of interventions targeting multiple risk behaviours in people aged 0–18 years found no effect on alcohol consumption for individual level (OR 1.02, 95% CI 0.80 to 1.31), family level (OR 0.83, 95% CI 0.47 to 1.46), or school level (OR 0.73, 95% CI 0.52 to 1.03) interventions, or on binge drinking [48].

Two studies were identified that included individuals with alcohol dependency or AUD. One study assessing injury-related deaths among individuals with alcohol dependency, abuse, or hazardous use found no significant effect (RR 0.65, 95% CI 0.21 to 2.00) [105]. One network meta-analysis on the effect of brief interventions on abstinence among individuals with AUD found no effect during treatment (OR 0.81, 95% CI 0.59 to 1.12) or after treatment (OR 0.76, 95% CI 0.46 to 1.22) [107]. The same study found that brief interventions in combination with either pharmacological interventions or psychological interventions had a lower probability of abstinence than pharmacological interventions. The certainty of evidence for brief interventions targeting AUD, either alone or in combination, was rated as very low due to low study quality and imprecision.

### Psychosocial intervention outcomes

A total of sixteen publications reported outcomes for psychosocial interventions [42, 46, 56, 60, 62–69, 101, 103, 107, 115]. Tables 5 and 6 summarise the outcomes for psychosocial interventions.

**Table 5:** Summary results of included systematic reviews, for psychosocial interventions

**Intervention**	**Population**	**Comparator**	**Outcome**	**Timeframe**	**Effect size** (method: effect (95% CI))	**I**^**2**^	**Publication bias**	**Number of RCTs**	**RCT quality assessment**
Psychotherapy (CBT, MI, BI) [107]	AUD	Control	Abstinent rate, during treatment	≥ 12 weeks	OR: 1.51 (1.04, 2.21)	67.3%	NR	4	NR
Psychotherapy (CBT, MI, BI) [107]	AUD	Control	Abstinent rate, after treatment	≥ 12 weeks	OR: 0.99 (0.57, 1.70)	67.3%	NR	2	NR
Psychotherapy (CBT, MI, BI) [107]	AUD	psychotherapy + BI	Abstinent rate, during treatment	≥ 12 weeks	OR: 1.20 (0.58, 2.46)	67.3%	NR	0 [NMA]	NR
Psychotherapy (CBT, MI, BI) [107]	AUD	psychotherapy + BI	Abstinent rate, after treatment	≥ 12 weeks	OR: 2.22 (0.89, 5.63)	67.3%	NR	0 [NMA]	NR
Psychotherapy (CBT, MI, miscellaneous) [104]	18–25 years (not in college)	Control group	Multiple (social consequences, AUDIT, ASI)	> 6 months	Cohen’s d: 0.18 (0.07, 0.29)	NR	NR	22	NR
**Cognitive behavioural therapy (CBT)**									
Lay health worker-led interventions (cognitive behavioural therapy) [115]	Adult patients with harmful or hazardous alcohol use	Enhanced usual care	Drinks per drinking day	up to 1 month	SMD: -0.37 (-0.52, -0.22)	0%	NR (< 4 studies)	2	1 study high risk blinding outcomes
Lay health worker-led interventions (cognitive behavioural therapy, counselling) [115]	Adult patients with harmful or hazardous alcohol use	Enhanced usual care	Amount of alcohol consumed	1 to 6 months	SMD: -0.23 (-0.56, 0.09)	61%	NR (< 4 studies)	3	1 study high risk blinding outcomes
Lay health worker-led interventions (counselling, cognitive behavioural therapy) [115]	Adult patients with harmful or hazardous alcohol use	Enhanced usual care	Amount of alcohol consumed	> 6 months	SMD: -0.11 (-0.29, 0.06)	42%	NR (< 4 studies)	2	1 study high risk blinding outcomes
CBT [103]	Alcohol dependence or AUD	Placebo	Continuous abstinence	84–365 days	OR: 0.53 (0.23, 1.22)	NA	NR	64	Low
Short form CBT [103]	Alcohol dependence or AUD	Placebo	Continuous abstinence	84–365 days	OR: 0.05 (0, 1.16)	NA	NR	64	Very low
Placebo + CBT [103]	Alcohol dependence or AUD	Placebo	Continuous abstinence	84–365 days	OR: 0.83 (0.28, 2.42)	NA	NR	64	Very low
**Contingency management (CM)**									
Contingency management [103]	Alcohol dependence or AUD	Placebo	Continuous abstinence	84–365 days	OR: 0.78 (0.17, 3.61)	NA	NR	64	Low
Contingency management [107]	AUD	Control	Abstinent rate, during treatment	≥ 12 weeks	OR: 0.30 (0.03, 2.59)	67.3%	NR	1	NR
Contingency management [107]	AUD	Control	Abstinent rate, after treatment	≥ 12 weeks	OR: 0.52 (0.07, 3.05)	67.3%	NR	1	NR
Contingency management [107]	AUD	CM + psychotherapy	Abstinent rate, during treatment	≥ 12 weeks	OR: 1.46 (0.12, 14.07)	67.3%	NR	0 [NMA]	NR
Contingency management [107]	AUD	CM + psychotherapy	Abstinent rate, after treatment	≥ 12 weeks	OR: 0.55 (0.07, 3.74)	67.3%	NR	0 [NMA]	NR
Contingency management [107]	AUD	Pharmacotherapy	Abstinent rate, during treatment	≥ 12 weeks	OR: 0.35 (0.03, 3.31)	67.3%	NR	0 [NMA]	NR
Contingency management [107]	AUD	Pharmacotherapy	Abstinent rate, after treatment	≥ 12 weeks	OR: 0.35 (0.05, 2.18)	67.3%	NR	0 [NMA]	NR
Contingency management [107]	AUD	Pharmacotherapy + BI	Abstinent rate, during treatment	≥ 12 weeks	OR: 0.68 (0.06, 5.98)	67.3%	NR	0 [NMA]	NR
Contingency management [107]	AUD	Pharmacotherapy + BI	Abstinent rate, after treatment	≥ 12 weeks	OR: 0.36 (0.05, 2.30)	67.3%	NR	0 [NMA]	NR
Contingency management [107]	AUD	Pharmacotherapy + psychotherapy	Abstinent rate, during treatment	≥ 12 weeks	OR: 0.51 (0.04, 4.78)	67.3%	NR	0 [NMA]	NR
Contingency management [107]	AUD	Pharmacotherapy + psychotherapy	Abstinent rate, after treatment	≥ 12 weeks	OR: 0.33 (0.04, 2.17)	67.3%	NR	0 [NMA]	NR
Contingency management [107]	AUD	Psychotherapy	Abstinent rate, during treatment	≥ 12 weeks	OR: 0.44 (0.04, 3.99)	67.3%	NR	0 [NMA]	NR
Contingency management [107]	AUD	Psychotherapy	Abstinent rate, after treatment	≥ 12 weeks	OR: 0.51 (0.07, 3.19)	67.3%	NR	0 [NMA]	NR
Contingency management [107]	AUD	Psychotherapy + BI	Abstinent rate, during treatment	≥ 12 weeks	OR: 0.54 (0.04, 5.09)	67.3%	NR	0 [NMA]	NR
Contingency management [107]	AUD	Psychotherapy + BI	Abstinent rate, after treatment	≥ 12 weeks	OR: 1.12 (0.14, 8.60)	67.3%	NR	0 [NMA]	NR
**Community reinforcement**									
Social network support [63]	Alcohol dependents	No social network support	Abstinence in the past 90 days	> 12 months	OR: 3.97 (2.26, 6.95)	NR	None	2	High risk of bias
**Motivational interview (MI)**									
Motivational Enhancement Therapy (MET) [103]	Alcohol dependence or AUD	Placebo	Continuous abstinence	84–365 days	OR: 0.45 (0.19, 1.11)	NA	NR	64	Very low
MI or motivational enhancement therapy (MI plus feedback) [65]	NR	Comparison group: traditional waitlist, information only groups, ‘treatment-as-usual	Self-reported consumption	NR	OR: 2.31 (1.75, 3.06)	NR	No	9	NR
Motivational interviewing [68]	Alcohol use	NR	Biochemically confirmed measures of alcohol use	4–6 months	Cohen’s d: 0.30 (0.03, 0.57)	NR	No	5	NR
Motivational Interviewing [60]	Young people aged up to 25 years, at risk	No intervention/placebo/ treatment as usual	Quantity of alcohol consumed	Less than four months follow-up	SMD: -0.25 (-0.37, -0.14)	45%	NR	33	NR
Motivational Interviewing [60]	Young people aged up to 25 years, at risk	No intervention/placebo/ treatment as usual	Quantity of alcohol consumed	4 + months follow up	SMD: -0.12 (-0.17, -0.07)	0.0%	No	26	Low
Motivational Interviewing [60]	Young people aged up to 25 years, at risk	No intervention/placebo/ treatment as usual	Frequency of alcohol consumption	4 + months follow up	SMD: -0.15 (-0.23, -0.07)	27%	NR	14	Low
Motivational Interviewing [60]	Young people aged up to 25 years, at risk	No intervention/placebo/ treatment as usual	Binge drinking	4 + months follow up	SMD: -0.06 (-0.12, 0.01)	0%	NR	16	Low
Motivational Interviewing [60]	Young people aged up to 25 years, at risk	No intervention/placebo/ treatment as usual	Alcohol problems	4 + months follow up	SMD: -0.11 (-0.19,-0.03)	52%	NR	22	Moderate
Motivational Interviewing [60]	Young people aged up to 25 years, at risk	No intervention/placebo/ treatment as usual	Quantity of alcohol consumed	4 + months follow-up	SMD: -0.14 (-0.20, -0.08)	22%	NR	39	moderate
Motivational Interviewing [56]	Adolescents (12–20 years) with AUD or problematic alcohol use	Treatment as usual	Days of alcohol use	NR	Net mean difference: -1.1 (-2.2, -0.3)	NA	NR	7	Moderate
Motivational interviewing [64]	Concurrent problem alcohol and illicit drug users	treatment as usual	Alcohol use as AUDIT or ASSIST scores	3 months	SMD: 0.04 (-0.29,0.37)	0%	NR	2	Low
**Family-oriented approach**									
Family-based prevention programmes [42]	School-aged children (< = 18 years)	No intervention/ standard care	Prevalence of alcohol use	NR	SMD: -0.16 (-0.36, 0.05)	NR	No	2	NR
Family-based prevention programmes [42]	School-aged children (< = 18 years)	No intervention/ standard care	Volume of alcohol use	NR	SMD: 0.06 (-0.15, 0.27)	NR	No	2	NR
Family-based prevention programmes [42]	School-aged children (< = 18 years)	No intervention /standard care	Frequency of alcohol use	NR	SMD: -0.65 (-1.64, 0.33)	97%	NR	5	NR
**Other**									
Coping skill training [103]	Alcohol dependence or AUD	Placebo	Continuous abstinence	84–365 days	OR: 0.35 (0.1, 1.19)	NR	NR	64	Very low
Cue Exposure Therapy; OR Cue Exposure Therapy and Coping Skills Training [67]	Adult participants (≥ 18) diagnosed with sub-clinical or clinical AUD	Active target conditions like cognitive behavioral therapy, relaxation or meditation and daily contact with assessment	Self-reported drinking days	3 months	SMD: -0.07 (-0.34, 0.49)	0%	Yes	2	Very low
Cue Exposure Therapy; OR Cue Exposure Therapy and Coping Skills Training [67]	Adult participants (≥ 18) diagnosed with sub-clinical or clinical AUD	Active target conditions like cognitive behavioral therapy, relaxation or meditation and daily contact with assessment	Self-reported drinking days	6 months	SMD: -0.21 (-0.48, 0.06)	24%	Yes	5	Very low
Cue Exposure Therapy; OR Cue Exposure Therapy and Coping Skills Training [67]	Adult participants (≥ 18) diagnosed with sub-clinical or clinical AUD	Active target conditions like cognitive behavioral therapy, relaxation or meditation and daily contact with assessment	Drinks per day	3 months	SMD: -0.07 (-0.48, 0.34)	0%	Yes	2	Very low
Cue Exposure Therapy; OR Cue Exposure Therapy and Coping Skills Training [67]	Adult participants (≥ 18) diagnosed with sub-clinical or clinical AUD	Active target conditions like cognitive behavioral therapy, relaxation or meditation and daily contact with assessment	Drinks per day	6 months	SMD: -0.16 (-0.52, 0.19)	64%	Yes	6	Very low
Cue Exposure Therapy; OR Cue Exposure Therapy and Coping Skills Training [67]	Adult participants (≥ 18) diagnosed with sub-clinical or clinical AUD	Active target conditions like cognitive behavioral therapy, relaxation or meditation and daily contact with assessment	Days with heavy drinking	6 months	SMD: -0.02 (-0.38, 0.41)	37%	Yes	2	Very low
Forming implementation intentions [66]	General population (no restrictions)	Passive and active control groups	Self-reported alcohol consumption	2 weeks to 3 months	OR: 0.31 (0.21, 0.42)	18%	No	16	Good
Home visit [101]	Pregnant women with alcohol problem	Usual care	Continued alcohol use	NR	RR: 1.18 (0.96, 1.46)	0%	NR	3	Low
Home visit [103]	Alcohol dependence or AUD	Placebo	Continuous abstinence	84–365 days	OR: 0.95 (0.32, 2.85)	NA	NR	64	Low
Mentoring interventions [69]	Alcohol use; children or adolescents	No intervention or wait list	Alcohol use	9–15 months	OR: 0.72 (0.58, 0.90)	0%	NR	2	Low risk of bias
Non-abstinent treatment strategies [62]	Alcohol dependent: adult	Abstinent-based treatment strategies	Controlled drinking goal	6–42 month	OR: 1.32 (0.51–3.39)	0%	No	2	High
**Combination**									
Psychotherapy + BI [107]	AUD	Control	Abstinent rate, during treatment	≥ 12 weeks	OR: 1.82 (0.90, 3.71)	67.3%	NR	2	NR
Psychotherapy + BI [107]	AUD	Control	Abstinent rate, after treatment	≥ 12 weeks	OR: 2.18 (0.84, 6.01)	67.3%	NR	0	NR
CM + psychotherapy [107]	AUD	Control	Abstinent rate, during treatment	≥ 12 weeks	OR: 0.20 (0.10, 0.40)	67.3%	NR	0	NR
CM + psychotherapy [107]	AUD	Control	Abstinent rate, after treatment	≥ 12 weeks	OR: 0.93 (0.44, 2.05)	67.3%	NR	0	NR
CM + psychotherapy [107]	AUD	Psychotherapy	Abstinent rate, during treatment	≥ 12 weeks	OR: 0.30 (0.17, 0.54)	67.3%	NR	10	NR
CM + psychotherapy [107]	AUD	Psychotherapy	Abstinent rate, after treatment	≥ 12 weeks	OR: 0.93 (0.51, 1.68)	67.3%	NR	3	NR
CM + psychotherapy [107]	AUD	Psychotherapy + BI	Abstinent rate, during treatment	≥ 12 weeks	OR: 0.37 (0.14, 0.90)	67.3%	NR	0	NR
CM + psychotherapy [107]	AUD	Psychotherapy + BI	Abstinent rate, after treatment	≥ 12 weeks	OR: 2.04 (0.71, 6.05)	67.3%	NR	0	NR

*ASSIST* Alcohol, Smoking and Substance Involvement Screening Test, *AUD* Alcohol use disorder, *AUDIT* AUD identification test, *BI* Brief intervention, *CBT* Cognitive behavioural therapy, *CI* Confidence interval, *CM* Contingency management, *MD* Mean difference, *MI* Motivational interviewing, *NMA* Network meta-analysis, *NR* Not reported, *OR* Odds ratio, *RCT* Randomized controlled trial, *RR* Relative risk, *SMD* Standard mean difference

**Table 6:** Certainty in evidence (GRADE) for psychosocial interventions

		Psychosocial interventions	Cognitive behavioural therapy	Contingency management	Community reinforcement	Motivational interviewing	Family-oriented approach	Mentoring	Coping skills	Cue exposure	Implementation intentions	Home visit	Controlled drinking
Risk of bias	Meta-analyses (AMSTAR)	Very low	Moderate	Low	Moderate	Adolescent moderate; others low	High	Low	Low	Low	Moderate	Moderate	High
	RCTs	NR	High	High	High	High	NR	Low	Very low	Very high	Low	High	Low
Inconsistency	Meta-analyses	Not consistent	Incomparable (population, time)	Consistent	NA (single study)	Adolescent consistent; others not	NA (single study)	NA (single study)	NA (single study)	NA (single study)	NA (single study)	Consistent	NA (single study)
	Heterogeneity of RCTs	High	Moderate	High	NR	Moderate adolescent, NR others	Very high	Low	NR	Moderate-high	Low	Low	Very low
Indirectness		AUD and emerging adults only	Moderately applicable	Applicable	Limited applicability	Applicable	Under 18 years only	Under 18 years only	Applicable (AUD)	Applicable (AUD)	Applicable	AUD and pregnant women	Applicable (AUD)
Imprecision	Confidence interval	Moderate	Wide	Very wide	Wide	Moderate	Mixed	Small	Very wide	Moderate	Small	Very wide	Very wide
	Sample size	Less than 100	Hundreds	Less than 100	Hundreds	Adolescents thousands; others NR	Hundreds	Thousands	Less than 100 (intervention)	Less than 100	Thousands	Hundreds	Less than 100
Small study effect (number of studies)		NR	NR	NR	No (2)	No (3), NR (3)	No (1)	NR (2)	NR (1)	Yes (1)	No (1)	NR (2)	No (1)
Other		NA	NA	NA	Large effect, long-term	Sub-group differences; short timeframe	NA	Long-term	NA	NA	Short timeframe	NA	Long-term
**Overall**		**Very low**	**Very low**	**Very low**	**Low**	**Adolescents—moderate; others—very low**	**Very low**	**Moderate**	**Very low**	**Very low**	**Moderate**	**Very low**	**Low**

*AUD* Alcohol use disorder, *NA* Not applicable, *NMA* Network meta-analysis, *NR* Not reported, *RCT* Randomised controlled trial

Two studies reported outcomes for cognitive behavioural therapy (CBT) [103, 115]. There was a significant decrease in alcohol consumption among hazardous/harmful drinkers in LMICs receiving CBT and counselling interventions from lay health workers up to 6 months post-intervention (SMD -0.23, 95% CI -0.56 to -0.09) [115], but the effect was not significant beyond 6 months (SMD -0.22, 95% CI -0.26 to 0.06). Among recently detoxified, alcohol dependent patients, one network meta-analysis found no effect on continuous abstinence (OR 0.53, 95% CI 0.23 to 1.22) [103]. We identified no studies reporting short-term outcomes among alcohol-dependent populations. The certainty of evidence was graded as very low due to risk of bias and imprecision.

For contingency management, two network meta-analyses considered abstinence among people with AUD or alcohol dependency [103, 107]. Neither found a significant effect of contingency management and all outcomes had very wide confidence intervals. Although one of the network meta-analyses showed considerable benefit of CM combined with psychotherapy (defined in the paper as BI, CBT, or motivational interviewing) relative to control (OR 0.20, 95% CI 0.10—0.40) and all other interventions, the effect was not maintained after treatment (OR 0.52, 95% CI 0.07 to 3.05) [107]. The body of evidence for contingency management was graded as very low due to high risk of bias and imprecision.

Only one meta-analysis was identified for the community reinforcement approach [63]. The study reported a very high reduction in abstinence among alcohol dependents after more than a year of follow-up (OR 3.97, 95% CI 2.26–6.95) for social network support. Although the long-term effect size was large and the meta-analysis was rated as moderate quality, the certainty of this finding was graded as low, since findings were based on two RCTs at high risk of bias with a total sample size of 210 participants. No studies for other types of community reinforcement approach were identified.

Six meta-analyses were identified for motivational interviewing, two of which were focussed on adolescents/young adults [56, 60], two on alcohol use in the general population [65, 68], one on people with AUD [103], and one on concurrent problem alcohol and illicit drug users [64]. Among adolescents and young adults, there were small reductions in alcohol consumption and alcohol problems, but not for binge drinking, over the intermediate term (over 4 months) among adolescents and young adults with risky drinking [60] and in days of alcohol use among adolescents with AUD or problematic alcohol use (MD -1.1, 95% CI -2.2 to -0.3) [56]. In the general population, one very low quality meta-analyses showed a considerable decrease in self-reported consumption for MI delivered in medical care settings (OR 2.31, 95% CI 1.75–3.06) [65] and among biochemically confirmed alcohol use after 4–6 months follow-up (Cohen’s d 0.30, 95% CI 0.03–0.57) [68]. However, no significant effect was found for abstinence maintenance after a year of follow-up among people with AUD (OR 0.45, 95% CI 0.19–1.11) [103] or for alcohol use among problem alcohol and illicit drug users after 3 months follow-up (SMD 0.04, 95% CI -0.29 to 0.37) [64]. The body of evidence for motivational interviewing in adolescents and young adults was graded as moderate due to good precision and consistency between studies, but very low among other population groups due to high risk of bias and small sample sizes.

One study was identified for family-oriented approaches, which reported on measures of alcohol use among school-aged children following family-based prevention programmes, administered to either the children themselves or to family members [42]. There was no significant effect across prevalence of alcohol use (SMD -0.16, 95% CI -0.36 to 0.05), volume of alcohol use (SMD 0.06, 95% CI -0.15 to 0.27), or frequency of alcohol use (SMD -0.65, 95% CI -1.64 to 0.33). Although the meta-analysis was rated as high methodological quality, the body of evidence was graded as very low due to high heterogeneity and limited generalisability to population groups outside of school children.

No reviews of mutual help groups met our inclusion criteria.

Among other psychosocial interventions identified, two reported on home visits [101, 103], one on coping skills training [103], and one on mentoring for children or adolescents with alcohol use [69]. For home visits, no significant effect was found in reducing continued alcohol use among pregnant women with alcohol problems (RR 0.18, 95% CI 0.96 to 1.46) [101] or in maintaining abstinence among recently detoxified, alcohol dependent patients (OR 0.95, 95% CI 0.32 to 2.85) [103], although the body of evidence was graded as very low due to high risk of bias and imprecision. For coping skills training, no significant effect was observed for promoting abstinence in recently detoxified, alcohol dependent patients (OR 0.95, 95% CI 0.32 to 2.85) [103]. Certainty in the evidence for coping skills training was graded as very low. For mentoring, there was a moderate reduction in alcohol use among children and adolescents after 9 to 15 months [69]. The body of evidence for mentoring among children and adolescents was graded as moderate due to low risk of bias, good precision, low heterogeneity, and the long timeframe.

Three reviews reported outcomes for specific psychosocial techniques, namely cue exposure therapy [67], forming implementation intentions [66], and controlled drinking non-abstinent strategies [62]. There was no evidence that cue exposure therapy affects alcohol consumption at 3- or 6-months follow-up among adults diagnosed with AUD or sub-clinical AUD, although the body of evidence was graded as very low due to high risk of bias, inconsistency in RCTs, publication bias and a very small sample size. Forming implementation intentions showed a large reduction in self-reported alcohol consumption in the general population over a short timeframe (up to 3 months) (OR 0.31, 95% CI 0.21, 0.42) [66]. Although risk of bias was low and there was good precision, certainty in the evidence was graded as moderate due to the short timeframe and single meta-analysis. Controlled drinking non-abstinent strategies were shown to be inferior to abstinence-based strategies among alcohol dependent adults at 6 to 42 months follow-up (OR 1.32, 95% CI 0.51 to 3.39) [62]. Body of evidence was downgraded to low due to wide confidence intervals and very small sample size.

Two reviews considered outcomes across psychosocial interventions. One review with very low AMSTAR rating found a small effect beyond 6 months for psychosocial interventions (primarily CBT and MI) delivered to emerging adults aged 18–25 years outside of college settings (Cohen’s d 0.18, 95% CI 0.07 to 0.29) [104], while a network meta-analysis considering the effect of CBT, MI and BI on promoting abstinence among people with AUD found a significant effect during treatment (OR 1.51, 95% CI 1.04 to 2.21) but not after (OR 0.99, 95% CI 0.57 to 1.70) [107]. Body of evidence was rated as very low.

### Digital intervention outcomes

Eighteen studies analysed the effect of digital interventions (encompassing internet-, computer-, and mobile-based interventions) (Tables 7 and 8). Of the two very low quality studies considering impact among individuals at high risk of AUD or diagnosis with AUD, one found a decrease in alcohol consumption up to 9 months following internet-based interventions (Hedge’s g 0.44, 95% CI 0.17 to 0.71) but noted very high heterogeneity (I^2^ = 81%) [110], while a review of e-interventions, including web-based and mobile applications, did not find a significant longer-term reduction in alcohol consumption at 6 months or 12 months, but did find a large improvement in abstinence (OR 1.94, 95% CI 1.14 to 3.31) [35]. Overall confidence in the evidence for digital interventions for alcohol dependent populations was graded as very low given risk of bias from study methods and high inconsistency.

**Table 7:** Summary results of included systematic reviews, for digital interventions

**Intervention**	Population	**Comparator**	**Outcome**	**Timeframe**	**Effect size** (method: effect (95% CI))	**I**^**2**^	**Publication bias**	**Number of RCTs**	**Quality assessment of RCTs**
Mobile text messaging [32]	Risky drinker	Minimal/no contact, basic health information up to once a week, referral to information sources or primary health care with reminders up to once a week, or intervention not focussed on alcohol consumption	Weekly alcohol consumption (grams)	1–15 months	MD: -18.62 (-39.61, 2.38)	66%	No	5	Low
			Heavy episode drinking per month	1–15 months	MD: -0.33 (-0.79, 0.12)	41%	Yes (very small)	7	Low
Computer-delivered intervention [102]	College or university students	No treatment control	Alcohol consumption per week/ month	≤ 13 weeks	WMD: 0.14 (0.03, 0.24)	80%	NR	28	Moderate
				14–26 weeks	WMD: 0.13 (− 0.01, 0.27)	0%	NR	8	Moderate
				≥ 27 weeks	WMD: 0.08 (− 0.09, 0.26)	0%	NR	5	Moderate
			Frequency of heavy drinking days	≤ 13 weeks	WMD: 0.13 (0.02, 0.24)	67%	NR	17	Moderate
				14–26 weeks	WMD: 0.17 (− 0.05, 0.39)	0%	NR	4	Moderate
				≥ 27 weeks	WMD: 0.13 (− 0.01, 0.26)	0%	NR	5	Moderate
e-interventions (including CD-ROM-based, web-based, IVR, or mobile applications [35]	Adults at high risk of AUD or a diagnosis of AUD	Inactive controls	Weekly alcohol consumption (grams)	6 months	MD: -25.0 (-59.3, 9.3)	54.5%	No	6	Low
				12 months	MD: -8.6 (-53.7, 36.5)	73%	No	5	Low
		Control	Abstinence	NR	OR: 1.94 (1.14, 3.31)	NR	No	3	Low
	College students at high risk of AUD or a diagnosis of AUD	Inactive controls	Weekly alcohol consumption (grams)	6 months	MD: -12.4 (-26.6, 1.9)	41%	No	8	Low
			Binge drinking episodes	6 months	MD: -0.1 (-1.0, 0.9)	45.5%	No	4	Moderate
BI web-based [36]	Military and veterans	Control	Self-reported alcohol consumption	6 – 20 months	WMD: 1.81 (-0.06, 3.68)	79.4%	NR	6	Mean quality rating score: 20 out of 32
			Death	6 months	RR: 0.42 (0.19, 0.94)	NR	NR	9	
				1 year	RR: 0.60 (0.40, 0.91)	NR	NR	9	
Electronic screening and brief intervention (eSBI) (brief intervention comprised of a single session, ranging from 5–45 min in duration, and up to a maximum of 4 sessions) [37]	Hazardous alcohol consumption	Control condition (care as usual, assessment only, non-intervention)	Weekly alcohol consumption (grams)	0–3 months	MD: -32.74 (-56.80, -8.68)	53.5%	No	9	Moderate
				3–6 months	MD: -17.33 (-31.82, -2.84)	30.4%	No	8	NR
				6–12 months	MD: -14.91 (-25.56, -4.26)	26.7%	No	10	NR
				> = 12 months	MD: -7.46 (-25.34, 10.43)	41.1%	No	7	NR
Social norms interventions delivered via web/ computer feedback [41]	University and college students	No intervention (assessment only or alcohol information or alternative (non-normative) intervention)	Number of drinking days per week	4 + months	SMD: -0.12 (-0.18, -0.05)	38%	Suspected	9	Moderate
Technology-based interventions (TBIs) such as website, text messages, and tablet [108]	Women of childbearing age (18 to 45 years old), any level of drinking behaviour	Inactive (no treatment, waitlist control, treatment as usual) or active (non–TBIs) controls	Combined outcomes of alcohol consumption	Min: 4 weeks; Max: 6 months	SMD: 0.13 (-0.03, 0.29)	NR	No	11	Moderate
Alcohol misuse prevention course (AlcoholEdu) [43]	College students with any drinking behaviour	No intervention	Quantity of alcohol use	0–3 months	SMD: -0.13 (- 0.22,—0.04)	NR	Yes	52	Low
				3–6 months	SMD: -0.07 (- 0.55, 0.42)	NR	Yes	52	Low
			Frequency of alcohol use	0–3 months	SMD: -0.04 (-0.15, 0.06)	NR	Yes	52	Low
Brief Alcohol Screening and Intervention for College Students (BASICS) [43]	College students with any drinking behaviour	No intervention	Quantity of alcohol use	0–3 months	SMD: -0.26 (-0.36, -0.16)	NR	Yes	52	Low
				3–6 months	SMD: -0.23 (-0.44, -0.02)	NR	Yes	52	Low
			Frequency of alcohol use	0–3 months	SMD: -0.36 (-0.55, -0.18)	NR	Yes	52	Low
Electronic CHECKUP TO GO (e-CHUG): Personalized prevention intervention to motivate individuals to reduce alcohol or marijuana consumption [43]	College students with any drinking behaviour	No intervention	Quantity of alcohol use	0–3 months	SMD: -0.25 (-0.45, -0.05)	NR	Yes	52	Low
				3–6 months	SMD:—0.12 (-0.39, 0.16)	NR	Yes	52	Low
			Frequency of alcohol use	0–3 months	SMD: -0.15 (-0.44, -0.14)	NR	Yes	52	Low
Tertiary Health Research Intervention Via Email (THRIVE) [43]	College students with any drinking behaviour	No intervention	Quantity of alcohol use	0–3 months	SMD:—0.47 (- 0.60,—0.33)	NR	Yes	52	Low
				3–6 months	SMD:—0.47 (-0.95, 0.02)	NR	Yes	52	Low
			Frequency of alcohol use	0–3 months	SMD:—0.15 (-0.44, 0.14)	NR	Yes	52	Low
Personalised digital intervention (including web-based, mobile phone text messaging, smartphone apps, social networking, or standalone computer-based technologies [45]	People living in the community	No or minimal intervention	Quantity of drinking (g/week)	1 to 12 months	MD: -22.84 (-30.31, -15.36)	78%	Yes	41	Moderate
			Frequency of drinking	1 to 12 months	MD: -0.16 (-0.24, -0.09)	39%	NR	16	Moderate
			Frequency of binge drinking	1 to 12 months	MD: -0.24 (-0.35, -0.13)	53%	NR	15	Moderate
			Binge drinkers	1 to 12 months	MD: 0.98 (0.97, 1.0)	0%	NR	9	NR
			Intensity of drinking	1 to 12 months	MD: -4.63 (-8.01, -1.23)	83%	NR	15	Moderate
		Face-to-face intervention	Quantity of drinking (g/week)	1 to 24 months	MD: 0.52 (-24.59, 25.63)	NA	NR	5	Low
			Frequency of binge drinking	1 to 24 months	MD: 0.04 (-0.15, 0.22)	0%	NR	3	Low
Distance-based alcohol moderation [50]	Cancer survivors, drank alcohol in the past week	No intervention or brochures	Combined outcomes (days of drinking, AUDIT score, consumption)	NR (mean: 10 months)	SMD: 0.12 (-0.08, 0.31)	0%	NR (inadequate studies)	3	Risk of bias concerns for all RCTs, especially in selection of the reported result
Personalised feedback [54]	College students and the adult population	Control condition (e.g., assessment only, waitlist, or minimal intervention)	Alcohol-drinking behaviour (e.g., frequency or quantity)	1.6 weeks to 9 months	SMD: 0.22 (0.16, 0.29)	0%	No	14	NR
Internet-based alcohol interventions (iAIs) including internet and CD-ROM [110]	Alcohol use disorders	Control conditions (information, assessment-only, waiting list)	Alcohol consumption	4 weeks to 9 months	Hedges g: 0.44 (0.17, 0.71)	81.08%	No	9	All studies used well-validated alcohol consumption measures and well-described, theoretically based interventions. Dropout rates differed from 0 to 42%
Low-intensity self-help intervention, performed on computer or mobile phone, with or without guidance from a professional [111]	Alcohol drinkers who exceeded local guidelines for low-risk drinking	Assessment only, waitlisted or alcohol information brochure control condition	Quantity consumed (mixture of subjective and objective)	0 months (post-test)	SMD: 0.2 (0.13, 0.27)	27%	Yes	16	The risk of bias varied among studies
				6–12 months	SMD: 0.06 (-0.14, 0.25)	NR	NR	8	NR
Internet-based alcohol interventions (iAIs) including internet, SMS, phone, CD-ROM [112]	Regular drinker and problem drinkers (exclude students and pregnant women)	Control condition (e.g., assessment only, waitlist, or minimal intervention)	Mean weekly alcohol consumption	1–12 month	MD: -5.02 (-7.57, -2.48)	90%	Yes	19	High
Computer-delivered intervention [113]	No specific population	Active comparison (cognitive-behavioural therapy: CBT), attention/placebo (assessment only, placebo, and treatment as usual)	Combined outcomes of alcohol consumptions	1–156 weeks	SMD 0.22 (0.14,0.29)	NR	NR	28	Non-significant Pearson correlations between treatment effect and methodological quality score (*r* = 0.13, *P* = 0.40)
Personalised normative feedback (able to be delivered remotely) [53]	Hazardous alcohol use	Passive control	Number of drinking days last month	12–23 months	SMD: -0.02 (-0.15, 0.11)	0%	NR	2	Low
			Symptom severity (RAPI score)	12–23 months	SMD: 0.13 (-0.01, 0.26)	0%	NR	2	Low
Personalised normative feedback combined with other self-directed interventions [53]	Hazardous alcohol use	Passive control	Number of drinking days last month	12–23 months	SMD: -0.01 (-0.14, 0.12)	0%	NR	2	Low
			Symptom severity (RAPI score)	12–23 months	SMD: 0.24 (0.11, 0.37)	0%	NR	2	Low
Computer assessment and feedback [55]	At risk drinking	Counsellor assessment and feedback	Alcohol consumption	0–6 months	SMD: -0.1 (-0.3, 0.11)	51%	NR	6	Very low
			Alcohol consumption	> 6 months	SMD: -0.11 (-0.53, 0.32)	81%	NR	2	Very low

*AUD* Alcohol use disorder, *AUDIT* AUD identification test, *CI* Confidence interval, *IVR* Interactive Voice Response, *MD* Mean difference, *NR* Not reported, *OR* Odds ratio, *RAPI* Rutgers Alcohol Problem Index, *RCT* Randomized controlled trial, *RR* Relative risk, *SMD* Standard mean difference

**Table 8:** Certainty in evidence (GRADE) for digital interventions

		e-interventions						Personalised digital interventions
		Hazardous/ harmful use	AUD	Students and young adults	Military and veterans	Women, child-bearing age	Cancer survivors	
Risk of bias	Meta-analyses (AMSTAR)	Low	Very low	Very low	Very low	Very low	Moderate	Very low
	RCTs	High	High	Moderate	Moderate	Moderate	High	High
Inconsistency	Meta-analyses	Inconsistent	Inconsistent	Consistent	NA (single study)	NA (single study)	NA (single study)	Consistent
	Heterogeneity of RCTs	Moderate	Moderate	Moderate	Very high	NR	Very low	Moderate
Indirectness		Applicable	Applicable	Applicable	US	May include low risk	May include low risk	Applicable
Imprecision	Confidence interval	Wide	Wide	Small	Moderate	Small	Moderate	Small
	Sample size	Thousands	Thousands	Thousands	Thousands	Thousands	Hundreds	Thousands
Small study effect (number of studies)		Yes (3), No (1)	No (2)	Yes (2), No (1), NR (3)	NR	No (1)	NR	Yes (1), No (1), NR (1)
Other		NA	NA	NA	Long-term	NA	NA	NA
**Overall**		**Very low**	**Very low**	**Very low**	**Very low**	**Very low**	**Very low**	**Very low**

Of the studies on hazardous/harmful drinkers in the general population, a review of electronic screening and brief intervention found a significant reduction in weekly alcohol consumption up to 3, 6 and 12 months (MD (grams) -14.91, 95% CI -25.56 to -4.26, 6–12 months) but not beyond 12 months (MD (grams) -7.46, 95% CI -25.34 to 10.43) [37]. Similarly, a review of internet interventions (including computer and mobile based interventions) found a small reduction in mean weekly alcohol consumption (MD -5.02, 95% CI -7.57 to -2.48) at 1–12 months follow-up, although heterogeneity among studies was very high (I^2^ = 90%) and there was evidence of publication bias [112]. A meta-analysis of low-intensity self-help interventions performed on a computer or mobile phone found a reduction in quantity of alcohol consumption immediately following delivery of the intervention (SMD 0.2, 95% CI 0.13 to 0.27) but not after 6–12 months (SMD 0.06, 95% CI -0.14 to 0.25) [111]. A single study focussed on mobile and text messaging interventions did not find a significant reduction in weekly alcohol consumption (MD (grams) -18.62, 95% CI -39.61 to 2.38) or heavy drinking episodes per month (MD -0.33, 95% CI -0.79 to 0.12), although confidence intervals were very wide [32]. Overall certainty in the evidence for digital interventions among hazardous and harmful drinkers was graded as very low due to risk of bias in study methods, presence of publication bias and inconsistency across studies.

Three studies evaluated personalised digital interventions delivered via web or mobile [45, 53, 54]. One study found a significant improvement across various measures of alcohol consumption, heavy drinking and binge drinking across a follow-up period of 1–12 months when compared with no intervention; moreover there was no significant difference in grams alcohol consumed per week (MD 0.52, 95% CI -24.59 to 25.63) or frequency of binge drinking (MD 0.04, 95% CI -0.15 to 0.22) when compared to face-to-face interventions for up to 24 months follow-up [45]. While another study also found a small improvement in drinking behaviour up to 9 months (SMD 0.22, 95% CI 0.16 to 0.29) [54], a review of remotely delivered personal normative feedback interventions found no improvement in drinking days (SMD -0.02, 95% CI -0.15 to 0.11) or symptom severity score (SMD 0.13, 95% CI -0.01 to 0.26) [53]. The body of evidence for personalised digital interventions was graded as very low certainty due to methodological risk of bias in the reviews and RCTs, as well as the presence of publication bias.

Among reviews of digital interventions in specific populations, 5 considered college and university students [35, 41, 43, 102, 113], 1 young people [55], 1 women aged 18–45 years [108], 1 military and veterans [36], and 1 cancer survivors [50]. A high-quality review found no significant difference between assessment and feedback delivered by computer as compared with assessment and feedback delivered by a counsellor (AMD -0.11, 95% CI -0.53 to 0.32, > 6 months) [55]. Although one review of computer-delivered interventions among college and university students found a decrease in alcohol consumption at 1 week to 3 years follow-up (SMD 0.22, 95% CI 0.14 to 0.29) [113], another found a very small reduction in alcohol consumption (WMD 0.14, 95% CI 0.03 to 0.24) and frequency of heavy drinking (WMD 0.13, 95% CI 0.02 to 0.24) up to 13 weeks post-intervention, but no significant effect at longer follow-up [102]. A review of social norms interventions delivered via web/computer, including tens of thousands of participants, found a small reduction in number of drinking days per week at more than 4 months follow-up (SMD -0.12, 95% CI -0.18 to -0.05) but noted suspected publication bias [41]. One network meta-analysis of digital intervention programmes found a significant reduction in quantity of alcohol consumed among all programmes (AlcoholEdu, BASICS, e-CHUG, THRIVE) at 0–3 months, but only BASICS showed continued benefit at 3–6 months (SMD -0.07, 95% CI -0.55 to 0.42) and the review found evidence of publication bias [43]. A single study considered e-interventions among college students at risk of, or diagnosed with, AUD and found no significant effect at 6 months follow-up for either alcohol consumption (MD (grams) -12.4, 95% CI -26.6 to 1.9) or binge drinking episodes (MD -0.1, 95% CI -1.0 to 0.9) [35]. Certainty in the evidence for digital interventions aimed at young people and college/university students was graded as low, as there was good precision and consistency, but poor methodological quality and publication bias.

Among military and veterans, web-based brief interventions showed no reduction in self-reported alcohol consumption at 6–20 months (WMD 1.81, 95% CI -0.06 to 3.68) but there was a significant reduction in deaths at 6 months and 12 months (RR 0.60, 95% CI 0.40 to 0.91, 12 months) [36]. Web or text-based interventions in women aged 18–45 years did not show a significant reduction in alcohol consumption at 1–6 months post-intervention (SMD 0.13, 95% CI -0.03 to 0.29), although it should be noted that any type of control intervention (inactive and active) was included [108]. Among cancer survivors, distance-based moderation showed no effect (SMD 0.12, 95% CI -0.08 to 0.31) [50]. Certainty in the evidence for military and veterans, women aged 18–45 years, and cancer survivors was downgraded to very low due to high risk of bias in review methods [36, 108] or RCTs [50].

### Pharmacological intervention outcomes

Outcomes for pharmacological interventions were reported by 30 studies [70–85, 87–98, 103, 107] and four additional studies reported outcomes for a combination of psychosocial and pharmacological interventions [86, 106, 107, 109]. Tables 9 and 10 summarise the outcomes for pharmacological interventions.

**Table 9:** Summary results of included systematic reviews, for pharmacological interventions

**Intervention**	**Population**	**Comparator**	**Outcome**	**Timeframe**	**Effect size** (method: effect (95% CI))	**I**^**2**^	**Publication bias**	**Number of RCTs**	**Quality assessment of RCTs**
**Anticonvulsants**									
Anticonvulsants [90]	Alcohol dependence	Placebo	Drinks/drinking day	11.9 weeks	MD: -1.49 (-2.32, -0.65)	31%	NR	11	NR
			Mean heavy drinking	11.2 weeks	MD: -0.35 (-0.51, -0.19)	34%	NR	12	NR
			Abstinence	15.5 weeks	MD: 1.21 (0.97, 1.52)	7%	NR	8	Moderate
Gabapentin [73]	AUD	Placebo	Alcohol consumption	NR	Hedge’s g: 0.14 (-0.35, 0.63)	82.2%	NR	4	NR
			Percentage heavy drinking days	NR	Hedge’s g: 0.55 (0.01, 1.08)	89%	NR	7	NR
			Percentage abstinent days	NR	Hedge’s g: 0.50 (-0.17, 1.16)	81.9%	NR	3	NR
			Abstinence rate	NR	OR: 1.47 (0.82, 2.65)	2%	NR	4	NR
Gabapentin 300–3600 mg/day [80]	Alcohol dependence or AUD (aged > = 18 years)	Placebo	Drink per day	NR	MD: -0.15 (-0.64, 0.35)	89%	None	5	Good
			Relapse of heavy drinking	NR	RR: 0.80 (0.57, 1.13)	65%	None	6	Good
			Percentage of heavy drinking days	NR	MD: -0.64 (-1.22, -0.06)	92%	None	7	Good
			Abstinence	NR	RR: 1.33 (0.84, 2.10)	44%	None	6	Good
			Percentage of days abstinent	NR	MD: 0.26 (-0.16, 0.69)	69%	None	4	Good
Carbamazepine [103]	Alcohol dependence or AUD	Placebo	Continuous abstinence	84–365 days	OR: 0.55 (0.08, 3.90)	NR	NR	64	Very low
Levetiracetam [103]	Alcohol dependence or AUD	Placebo	Continuous abstinence	84–365 days	OR: 1.03 (0.46, 2.34)	NR	NR	64	Low
Oxcarbazepine [103]	Alcohol dependence or AUD	Placebo	Continuous abstinence	84–365 days	OR: 2.46 (0.91, 6.61)	NR	NR	64	Very low
Pregabalin [103]	Alcohol dependence or AUD	Placebo	Continuous abstinence	84–365 days	OR: 1.97 (0.58, 6.74)	NR	NR	64	Low
Topiramate [103]	Alcohol dependence or AUD	Placebo	Continuous abstinence	84–365 days	OR: 1.88 (1.06, 3.34)	NR	NR	64	Very low
Topiramate [89]	Alcohol dependence or AUDs	Placebo	Total alcohol consumption	3–52 weeks	SMD: -0.77 (-1.12, -0.42) NMD: -0.79 (-1.21, -0.36)	0%, 51.5%	Yes	2	Very low
			Non-drinking days	3–52 weeks	SMD: 0.45 (0.15, 0.75) NMD: 0.42 (0.19, 0.66)	62.9% 41.2%	Yes	3	NR
			Drinks per drinking day	3–52 weeks	SMD: -0.40 (-0.88, 0.09) NMD: -0.39 (-0.83, 0.04)	79.1% 61.5%	Yes	2	NR
			Drinking days	3–52 weeks	SMD: -0.75 (-1.46, -0.05) NMD: -0.75 (-1.53, 0.02)	NA 46.4%	Yes	1	NR
			Heavy drinking day	3–52 weeks	SMD: -0.59 (-0.96, -0.22) NMD: -0.49 (-0.71, -0.27)	73.8% 38.4%	Yes	4	Very low
			Mortality	3–52 weeks	OR: 0.14 (0, 7.01)	NA	Yes	3	NR
**Antidepressants**									
SSRI-based [96]	Alcohol disorder and major depressive disorder	Placebo	Alcohol abstinence	NR	OR: 1.26 (0.06, 2.56)	30%	NR	3	NR
Nefazodone [96]	Alcohol disorder and major depressive disorder	Placebo	Alcohol abstinence	NR	OR: 2.18 (0.68, 7.07)	0%	NR	2	NR
Mirtazapine [76]	AUD; comorbid depression/depressive symptoms	Control	AUD remission rate	NR	MD: -0.95 (-86.65, 83.82) SMD: -0.78 (-1.69, 0.13)	NR	No	NR	Low-moderate quality (all outcomes)
SARI [76]	AUD; comorbid depression/depressive symptoms	Control	AUD remission rate	NR	OR: 1.85 (0.62, 5.66) MD: 19.49 (-6.59, 45.62) SMD: -0.23 (-1.38, 0.92)	NR	No	NR	Low-moderate quality (all outcomes)
		NRI	AUD remission rate	NR	OR: 1.60 (0.2, 12.0)	NR	No	NR	
		SSRI	AUD remission rate	NR	OR: 1.53 (0.46, 5.0)	NR	No	NR	
		Tricyclic antidepressants	AUD remission rate	NR	OR: 1.12 (0.3, 5.2)	NR	No	NR	
NRI [76]	AUD; comorbid depression/depressive symptoms	Control	AUD remission rate	NR	OR: 1.15 (0.21, 6.31) SMD: -2.44 (-3.53, -1.36)	NR	No	NR	Low-moderate quality (all outcomes)
		SSRI	AUD remission rate	NR	OR: 0.96 (0.2, 5.3)	NR	No	NR	
		Tricyclic antidepressants	AUD remission rate	NR	OR: 0.70 (0.1, 5.1)	NR	No	NR	
SSRI [76]	AUD; comorbid depression/depressive symptoms	Control	AUD remission rate	NR	OR: 1.21 (0.78, 1.92) MD: 0.45 (-6.35, 7.36) SMD: -0.33 (-0.90, 0.24)	NR	No	NR	Low-moderate quality (all outcomes)
		Tricyclic antidepressants	AUD remission rate	NR	OR: 0.73 (0.2, 2.3)	NR	No	NR	
SSRI [70]	AUD; depression patients	Placebo	Drinks per drinking days	NR	MD: -1.42 (-2.58, -0.26)	15%	Yes	3	Moderate
			Number of abstinent participants	NR	RR: 1.66 (1.02, 2.68)	20%	Yes	4	Moderate
Tricyclic antidepressants [76]	AUD; comorbid depression/depressive symptoms	Control	AUD remission rate	NR	OR: 1.65 (0.57, 4.73) MD: 2.50 (-21.44, 26.40) SMD: -0.31 (-1.11, 0.49)	NR	No	NR	Low-moderate quality (all outcomes)
5-HT2 antagonist [70]	AUD; depression patients	Placebo	Drinks per drinking days	NR	MD: -1.06 (-2.00, -0.11)	0%	Yes	3	Moderate
Citalopram/ escitalopram [103]	Alcohol dependence or AUD	Placebo	Continuous abstinence	84–365 days	OR: 1.03 (0.33, 3.16)	NR	NR	64	Low
Fluoxetine [103]	Alcohol dependence or AUD	Placebo	Continuous abstinence	84–365 days	OR: 2.97 (0.97, 9.05)	NR	NR	64	Very low
Fluvoxamine [103]	Alcohol dependence or AUD	Placebo	Continuous abstinence	84–365 days	OR: 1.03 (0.57, 1.88)	NR	NR	64	Low
Nefazodone [103]	Alcohol dependence or AUD	Placebo	Continuous abstinence	84–365 days	OR: 0.57 (0.19, 1.76)	NR	NR	64	Very low
Tianeptine [103]	Alcohol dependence or AUD	Placebo	Continuous abstinence	84–365 days	OR: 1.22 (0.58, 2.57)	NR	NR	64	Low
Tiapride [103]	Alcohol dependence or AUD	Placebo	Continuous abstinence	84–365 days	OR: 0.56 (0.3, 1.05)	NR	NR	64	Moderate
Trazodone [103]	Alcohol dependence or AUD	Placebo	Continuous abstinence	84–365 days	OR: 0.61 (0.2, 1.84)	NR	NR	64	Very low
**Antipsychotics**									
Antipsychotics [76]	AUD; comorbid depression/depressive symptoms	Control	AUD remission rate	NR	OR: 0.97 (0.30, 3.22) MD: 9.56 (-13.88, 32.99) SMD: -0.03 (-1.42, 1.36)	NR	No	NR	Low-moderate quality (all outcomes)
		Baclofen	AUD remission rate	NR	OR: 0.56 (0.2, 2.1)	NR	No	NR	
		Bromocriptine	AUD remission rate	NR	OR: 2.65 (0.4, 22.0)	NR	No	NR	
		Buspirone	AUD remission rate	NR	OR: 0.57 (0.1, 3.2)	NR	No	NR	
		Disulfiram	AUD remission rate	NR	OR: 0.19 (0.04, 0.9)	NR	No	NR	
		Lithium	AUD remission rate	NR	OR: 1.40 (0.4, 6.3)	NR	No	NR	
		Memantine	AUD remission rate	NR	OR: 0.86 (0.1, 5.2)	NR	No	NR	
		Naltrexone	AUD remission rate	NR	OR: 0.70 (0.2, 2.5)	NR	No	NR	
		Naltrexone + disulfiram	AUD remission rate	NR	OR: 0.38 (0.1, 2.2)	NR	No	NR	
		Naltrexone + SSRI	AUD remission rate	NR	OR: 0.44 (0.1, 1.7)	NR	No	NR	
		SARI	AUD remission rate	NR	OR: 0.53 (0.1, 2.7)	NR	No	NR	
		NRI	AUD remission rate	NR	OR: 0.85 (0.1, 6.7)	NR	No	NR	
		SSRI	AUD remission rate	NR	OR: 0.81 (0.2, 2.8)	NR	No	NR	
		Tricyclic antidepressant	AUD remission rate	NR	OR: 0.59 (0.1, 22.9)	NR	No	NR	
Antipsychotics (all) [79]	Alcohol dependence	Placebo	Relapse	2–52 weeks	RR: 1.05 (0.95, 1.16)	68%	No	9	High
			Abstinence/drinking days	12—52 weeks	RR: 0.17 (0.01, 0.33)	29%	No	5	High
Aripiprazole [79]	Alcohol dependence	Placebo	Relapse	2–12 weeks	RR: 1.07 (0.92, 1.24)	70%	NR	2	High
Quetiapine [79]	Alcohol dependence	Placebo	Relapse	12 weeks	RR: 0.87 (0.65, 1.17)	78%	NR	2	High
Tiapride [79]	Alcohol dependence	Placebo	Relapse	24–26 weeks	RR: 1.07 (0.67, 1.71)	88%	NR	2	High
Amisulpride [103]	Alcohol dependence or AUD	Placebo	Continuous abstinence	84–365 days	OR: 0.39 (0.09, 1.64)	NA	NR	64	Low
Aripiprazole [103]	Alcohol dependence or AUD	Placebo	Continuous abstinence	84–365 days	OR: 1.49 (0.43, 5.18)	NA	NR	64	Low
Flupentixol [103]	Alcohol dependence or AUD	Placebo	Continuous abstinence	84–365 days	OR: 0.44 (0.2, 0.95)	NA	NR	64	Very low
Quetiapine [103]	Alcohol dependence or AUD	Placebo	Continuous abstinence	84–365 days	OR: 6.75 (1.2, 38.05)	NA	NR	64	Low
**Aversive agents**									
Disulfiram [76]	AUD; comorbid depression/depressive symptoms	Control	AUD remission rate	NR	OR: 5.00 (1.97, 12.95) MD: 17.03 (3.83, 30.47) SMD: 0.31 (-0.99, 1.61)	NR	No	NR	Low-moderate quality (all outcomes)
		Lithium	AUD remission rate	NR	OR: 7.19 (2.2, 27.0)	NR	No	NR	
		Memantine	AUD remission rate	NR	OR: 4.44 (0.8, 23.0)	NR	No	NR	
		Naltrexone	AUD remission rate	NR	OR: 3.62 (1.4, 9.3)	NR	No	NR	
		Naltrexone + disulfiram	AUD remission rate	NR	OR: 1.94 (0.4, 8.0)	NR	No	NR	
		Naltrexone + SSRI	AUD remission rate	NR	OR: 2.25 (0.7, 6.9)	NR	No	NR	
		SARI	AUD remission rate	NR	OR: 2.74 (0.6, 12.0)	NR	No	NR	
		NRI	AUD remission rate	NR	OR: 4.36 (0.6, 30.0)	NR	No	NR	
		SSRI	AUD remission rate	NR	OR: 4.16 (1.5, 12.0)	NR	No	NR	
		Tricyclic antidepressant	AUD remission rate	NR	OR: 3.04 (0.7, 13.0)	NR	No	NR	
Disulfiram [77]	AUD; adult	Placebo or another medication	Return to any drinking	NR	RD: -0.04 (-0.11 to 0.03)	0%	NR	2	Moderate
Disulfiram [78]	Alcohol dependent patients	Other treatment	Abstinence	12 months	OR: 1.83 (1.02, 3.29)	94%	NR	2	NR
		Placebo	Abstinence	12 month	OR: 1.48 (0.98, 2.23)	0%	NR	2	NR
		Other or no treatment	Abstinence	NR	OR: 2.24 (1.69, 2.97)	77%	NR	9	NR
Supervised disulfiram [78]	Alcohol dependent patients	Other or no treatment	Abstinence	NR	OR: 3.89 (2.66, 5.58)	84%	NR	7	NR
Unsupervised disulfiram [78]	Alcohol dependent patients	Other or no treatment	Abstinence	NR	OR: 1.59 (1.07, 2.37)	34%	NR	3	NR
Disulfiram [94]	Alcohol abuse or dependent	Control	Combined measure	8—52 weeks	Hedges' g: 0.58 (0.35, 0.82)	72%	Yes	22	NR
		No disulfiram	Combined measure	NR	Hedges' g: 0.43, (0.17, 0.69)	44%	NR	8	NR
Disulfiram [103]	Alcohol dependence or AUD	Placebo	Continuous abstinence	84–365 days	OR: 0.93 (0.48, 1.79)	NR	NR	64	Low
**Baclofen**									
Baclofen [71]	Alcohol dependence	Placebo	Amount of drinking	NR	SMD: 0.28 (0.00, 0.56)	71.9%	No	10	high
Baclofen [76]	AUD; comorbid depression/depressive symptoms	Control	AUD remission rate	NR	OR: 1.75 (0.97, 3.29) MD: 10.42 (1.70, 19.33) SMD: -0.25 (-1.03, 0.54)	NR	No	NR	Low-moderate quality (all outcomes)
		Bromocriptine	AUD remission rate	NR	OR: 4.76 (0.8, 30.0)	NR	No	NR	
		Buspirone	AUD remission rate	NR	OR: 1.03 (0.3, 4.1)	NR	No	NR	
		Disulfiram	AUD remission rate	NR	OR: 0.35 (0.1, 1.1)	NR	No	NR	
		Lithium	AUD remission rate	NR	OR: 2.49 (0.96, 7.6)	NR	No	NR	
		Memantine	AUD remission rate	NR	OR: 1.54 (0.3, 6.9)	NR	No	NR	
		Naltrexone	AUD remission rate	NR	OR: 1.26 (0.6, 2.8)	NR	No	NR	
		Naltrexone + disulfiram	AUD remission rate	NR	OR: 0.68 (0.2, 2.9)	NR	No	NR	
		Naltrexone + SSRI	AUD remission rate	NR	OR: 0.78 (0.3, 2.0)	NR	No	NR	
		SARI	AUD remission rate	NR	OR: 0.95 (0.3, 3.3)	NR	No	NR	
		NRI	AUD remission rate	NR	OR: 1.52 (0.3, 9.2)	NR	No	NR	
		SSRI	AUD remission rate	NR	OR: 1.44 (0.7, 3.1)	NR	No	NR	
		Tricyclic antidepressant	AUD remission rate	NR	OR: 0.22 (0.03, 1.6)	NR	No	NR	
Baclofen [82]	Alcohol-dependent patients	Placebo	Percentage abstinent patients	4–12 weeks	RR: 2.79 (1.79, 4.34)	0%	No	3	Low
			Mean abstinent days	4–12 weeks	SMD: 3.69 (0.74, 8.11)	96%	No	3	Low
Baclofen [85]	Alcohol use disorder	Placebo	Relapse: return to any drinking	4–52 weeks	RR: 0.88 (0.74, 1.04)	76.65%	NR	5	Moderate
			Percentage of heavy drinking days	NR	MD: 0.25 (-1.25, 1.76)	0%	NR	3	Moderate
			Drinks per drinking day	NR	MD: 1.55 (1.32, 1.77)	0%	NR	2	Low
			Percentage days abstinent	NR	MD: 0.39 (-11.51, 12.29)	96%	NR	6	Low
Baclofen [89]	Alcohol dependence or AUDs	Placebo	Total alcohol consumption	3–52 weeks	SMD: -1.00 (-1.80, -0.19) NMD: -1.00 (-1.86, -0.13)	NA	Yes	1	Very low
			Non-drinking days	3–52 weeks	SMD -0.08 (-0.44, 0.27) NMD -0.09 (-0.50, 0.32)	0%, 41.2%	Yes	2	NR
			Heavy drinking days	3–52 weeks	SMD 0.03 (-0.33, 0.39) NMD 0.03 (-0.37, 0.44)	0%, 38.4%	Yes	2	Very low
Baclofen [91]	AUD with heavy drinking, craving, anxiety and depression	Placebo	Heavy drinking days	3–26 weeks	SMD: -0.26 (-0.68, 0.15)	95%	Yes	6	Acceptable
			Abstinent days	3–26 weeks	SMD: 0.03 (-0.10, 0.15)	23%	Yes	6	Acceptable
Baclofen [103]	Alcohol dependence or AUD	Placebo	Continuous abstinence	84–365 days	OR: 4.63 (1, 21.48)	NA	NR	64	Low
**Benzodiazepine**									
**Glutamate antagonist**									
Acamprosate [72]	Alcohol dependence	Placebo	Abstinence rate	1 – 12 months	OR: 1.88 (1.57, 2.25)	NR	Yes	11	NR
			Cumulative abstinence duration	3 – 12 months	WMD: 26.55 (17.56, 35.54)	NR	NR	7	NR
Acamprosate [74]	Alcohol dependence or harmful alcohol use/alcohol abuse	Placebo	Abstinence	6 months	RR: 0.83 (0.78,0.89)	64%	Yes	17	Moderate
Acamprosate [76]	AUD; comorbid depression/depressive symptoms	Control	AUD remission rate	NR	OR: 1.66 (0.89, 3.05) MD: 6.79 (-2.30, 16.19) SMD: -0.4 (-1.45, 0.60)	NR	No	NR	Low-moderate quality (all outcomes)
		Antiepileptics	AUD remission rate	NR	OR: 0.65 (0.3, 1.6)	NR	No	NR	
		Antipsychotics	AUD remission rate	NR	OR: 1.72 (0.4, 6.4)	NR	No	NR	
		Baclofen	AUD remission rate	NR	OR: 0.96 (0.3, 3.8)	NR	No	NR	
		Bromocriptine	AUD remission rate	NR	OR: 4.55 (0.8, 28.0)	NR	No	NR	
		Buspirone	AUD remission rate	NR	OR: 0.98 (0.3, 3.8)	NR	No	NR	
		Disulfiram	AUD remission rate	NR	OR: 0.33 (0.1, 0.9)	NR	No	NR	
		Lithium	AUD remission rate	NR	OR: 2.39 (0.1, 0.9)	NR	No	NR	
		Memantine	AUD remission rate	NR	OR: 1.48 (0.3, 6.5)	NR	No	NR	
		Naltrexone	AUD remission rate	NR	OR: 1.21 (0.6, 2.4)	NR	No	NR	
		Naltrexone + disulfiram	AUD remission rate	NR	OR: 0.65 (0.2, 2.6)	NR	No	NR	
		Naltrexone + SSRI	AUD remission rate	NR	OR: 0.75 (0.3, 1.8)	NR	No	NR	
		SARI	AUD remission rate	NR	OR: 0.91 (0.3, 3.1)	NR	No	NR	
		NRI	AUD remission rate	NR	OR: 1.45 (0.2, 8.6)	NR	No	NR	
		SSRI	AUD remission rate	NR	OR: 1.39 (0.6, 2.9)	NR	No	NR	
		Tricyclic antidepressants	AUD remission rate	NR	OR: 0.99 (0.3, 3.4)	NR	No	NR	
Acamprosate [77]	AUD; adult	Placebo or another medication	Return to any drinking	NR	RD: -0.09 (-0.14, -0.04)	80.8%	NR	16	Low and moderate
			Return to heavy drinking	NR	RD: -0.01 (-0.04, 0.03)	0%	NR	7	Low and moderate
			% Drinking Days	NR	WMD: -8.8 (-12.8, -4.8)	NR	NR	13	Moderate
Acamprosate [83]	Alcohol dependence	Placebo	Continuous abstinence	3 months	RR: 1.33 (1.20, 1.47)	NR	NR	NR	NR
				6 months	RR: 1.47 (1.29, 1.69)	NR	No	17	Moderate
				12 months	RR: 1.95 (1.58, 2.42)	NR	NR	5	NR
Acamprosate [84]	Alcohol dependence	Placebo	Mean percentage abstinent days	NR	MD: 10.38 (7.10, 13.65)	NR	NR	NR	NR
			Abstinence: Abstinence rate	NR	OR: 1.87 (1.57, 2.23)	NR	NR	NR	NR
Acamprosate [95]	Adults with alcohol dependence	Placebo	Cumulative abstinence duration	3 months	RR: 1.76 (1.14, 2.39)	NR	NR	9	Good
				6 months	RR: 1.16 (1.03, 1.16)	NR	NR	8	Good
				12 months	RR: 1.11 (1.01, 1.21)	NR	NR	6	Good
Acamprosate [103]	Alcohol dependence or AUD	Placebo	Continuous abstinence	84–365 days	OR: 1.86 (1.49, 2.33)	NR	NR	64	Moderate
Acamprosate [92]	Adults with alcohol dependence	Placebo	Return to any drinking	12 months	RR: 0.86 (0.81, 0.91)	79.11%	No	24	NR
		Placebo	Cumulative abstinence duration	12 months	MD: 10.94 (5.08, 16.81)	94.32%	No	19	NR
**Opioid antagonist**									
Nalmefene [88]	Alcohol dependence	Placebo	Total alcohol consumption	6 months	SMD: -0.2 (-0.3, -0.1)	NR	NR	5	NR
			Monthly number of heavy drinking days	6 months	MD: − 1.65 (− 2.41, − 0.89)	NR	NR	5	NR
			Mortality	6 months	RR: 0.43 (0.08, 2.38)	0%	NR	4	NR
Nalmefene [89]	Alcohol dependence or AUDs	Placebo	Total alcohol consumption	3–52 weeks	SMD: -0.19 (-0.29, -0.10) NMD: -0.18 (-0.35, -0.02)	0%, 51.5%	Yes	7	Moderate
			Non-drinking days	3–52 weeks	SMD: 0.09 (-0.01, 0.19) NMD: 0.10 (-0.05, 0.25)	0%, 38.4%	Yes	8	NR
			Drinks per drinking day	3–52 weeks	SMD: -0.26 (-0.48, -0.05) NMD: -0.24 (-0.65, 0.18)	0% 61.5%	Yes	3	NR
			Heavy drinking days	3–52 weeks	SMD: -0.22 (-0.32, -0.12) NMD: -0.18 (-0.35, -0.02)	0%, 51.5%	Yes	7	Moderate
			Mortality	3–52 weeks	SMD: 0.41 (0.08, 2.11)	0%	Yes	9	NR
Naltrexone [89]	Alcohol dependence or AUDs	Placebo	Total alcohol consumption	3–52 weeks	SMD: -0.11 (-0.40, 0.18) NMD: -0.09 (-0.29, 0.11)	75.6% 51.5%	Yes	5	Very low
			Non-drinking days	3–52 weeks	SMD: -0.28 (-0.95, 0.40) NMD: -0.21 (-0.52, 0.10)	82.8% 41.2%	Yes	3	NR
			Drinks per drinking day	3–52 weeks	SMD: -0.04 (-0.31, 0.23) NMD: -0.05 (-0.30, 0.21)	66.1% 61.5%	Yes	8	NR
			Drinking day	3–52 weeks	SMD: -0.16 (-0.35, 0.04) NMD: -0.16 (-0.35, 0.04)	46.4%	Yes	6	NR
			Heavy drinking days	3–52 weeks	SMD -0.03 (-0.21, 0.16) NMD -0.04 (-0.19, 0.12)	51%, 38.4%	Yes	8	Very low
Naltrexone [103]	Alcohol dependence or AUD	Placebo	Continuous abstinence	84–365 days	OR: 1.36 (0.97, 1.91)	NR	NR	64	Low
Naltrexone [76]	AUD; comorbid depression/depressive symptoms	Control	AUD remission rate	NR	OR: 1.38 (0.88, 2.18) MD: 4.76 (-3.59, 13.13) SMD: 0.11 (-0.68, 0.89)	NR	No	NR	Low-moderate quality (all outcomes)
		Naltrexone + disulfiram	AUD remission rate	NR	OR: 0.54 (0.1, 1.9)	NR	No	NR	
		Naltrexone + SSRI	AUD remission rate	NR	OR: 0.62 (0.3, 1.3)	NR	No	NR	
		SARI	AUD remission rate	NR	OR: 0.75 (0.2, 2.4)	NR	No	NR	
		NRI	AUD remission rate	NR	OR: 1.20 (0.2, 6.8)	NR	No	NR	
		SSRI	AUD remission rate	NR	OR: 1.15 (0.62, 2.0)	NR	No	NR	
		Tricyclic antidepressant	AUD remission rate	NR	OR: 0.84 (0.3, 2.7)	NR	No	NR	
Naltrexone, 50 mg oral [77]	AUD; adults	Placebo or another medication	Return to any drinking	NR	RD: -0.05 (-0.10, -0.002)	46.4%	NR	16	Low and moderate
			Return to heavy drinking	NR	RD: -0.09 (-0.13, -0.04)	43.7%	NR	19	Low and moderate
			% Drinking Days	NR	WMD: -5.4 (-7.5, -3.2)	NR	NR	15	Moderate
			% Heavy Drinking Days	NR	WMD: -4.1 (-7.6, -0.61)	NR	NR	6	Moderate
			Drinks per Drinking Day	NR	WMD: -0.49 (-0.92, -0.06)	NR	NR	9	Low
Naltrexone, 100 mg oral [77]	AUD; adults	Placebo or another medication	Return to any drinking	NR	RD: -0.03 (-0.08, 0.02)	0%	NR	3	Low to moderate
			Return to heavy drinking	NR	RD: -0.05 (-0.11, 0.01)	0%	NR	2	Low to moderate
			% Drinking Days (DDs)	NR	WMD: -0.9 (-4.2, 2.5)	NR	NR	2	Low
			% Heavy Drinking Days	NR	WMD: -3.1 (-5.8, -0.3)	NR	NR	2	Low
Naltrexone injection [77]	AUD; adults	Placebo or another medication	Return to any drinking	NR	RD: -0.04 (-0.10, 0.03)	58.5%	NR	2	moderate
			Return to heavy drinking	NR	RD: -0.01 (-0.14, 0.13)	72.2%	NR	2	moderate
			% Heavy Drinking Days (HDDs)	NR	WMD: -4.6 (-8.5, -0.56)	NR	NR	2	Low
Naltrexone [93]	AUD	Placebo	Drinking days	NR	MD: -3.89 (-5.75, -2.04)	94.4%	NR	26	NR
			Heavy drinking days	NR	MD: -3.25 (-5.51, -0.99)	81%	NR	15	NR
Naltrexone [72]	Alcohol dependence	Placebo or reference group	Drinking days	12 weeks	WMD: -4.49 (-5.22, -3.77)	NR	Yes	7	NR
			Drinks/ drinking days	12 weeks	WMD -0.75 (-1.2, -0.29)	NR	Yes	5	NR
			Total alcohol consumption (g/ week)	12 weeks	WMD -100 (-107, -109)	NR	Yes	2	NR
			Heavy drinking days	Min: 12 weeks; Max: 9 months	WMD -1.1 (-2.0, -0.21)	NR	Yes	2	NR
			Abstinence rate	Min: 12 weeks; Max: 9 months	OR: 1.26 (0.97, 1.64)	NR	Yes	10	Good quality
			Relapse rate	Min: 12 weeks; Max: 9 months	OR: 0.62 (0.52, 0.75)	NR	Yes	14	Good quality
Naltrexone [95]	Adults with alcohol dependence	Placebo	Cumulative abstinence duration (CAD)	3 months	RR: 1.23 (1.00 1.78)	NR	NR	8	Good
			Relapse rate	3 months	RR: 1.2 (1.17, 1.47)	NR	NR	18	Good
Naltrexone [97]	Alcohol dependence or abuse (aged > = 18 years)	Placebo	Mean percentage of drinking days	12 weeks	MD: –2.8 (–5.8, –0.2)	NR	NR	5	NR
			Abstinence rate	12 weeks	RR: 1.28 (1.08, 1.52)	NR	NR	7	NR
**Other**									
Alpha-blocker [98]	AUD: adult	Placebo	Drink/ day or week	6–13 week	SMD: -0.32 (-0.56, -0.07)	15%	No	6	NR
			Heavy drinking days	NR	SMD: -0.44 (-0.94, 0.06)	76%	No	5	NR
Antiepileptic [76]	AUD; comorbid depression/depressive symptoms	Control	AUD remission rate	NR	OR: 2.55 (1.26, 5.22) MD: 16.94 (-0.80,34.58) SMD: -0.70 ( -2.05, 0.65)	NR	No	NR	Low-moderate quality (all outcomes)
		Antipsychotics	AUD remission rate	NR	OR: 2.63 (0.7, 10.0)	NR	No	NR	Low-moderate quality (all outcomes)
		Baclofen	AUD remission rate	NR	OR: 1.47 (0.6, 3.7)	NR	No	NR	
		Bromocriptine	AUD remission rate	NR	OR: 6.98 (1.2, 45.0)	NR	No	NR	
		Buspirone	AUD remission rate	NR	OR: 1.51 (0.4, 6.2)	NR	No	NR	
		Disulfiram	AUD remission rate	NR	OR: 0.51 (0.2, 1.6)	NR	No	NR	
		Lithium	AUD remission rate	NR	OR: 3.67 (1.3, 11.0)	NR	No	NR	
		Memantine	AUD remission rate	NR	OR: 2.26 (0.5, 10.0)	NR	No	NR	
		Naltrexone	AUD remission rate	NR	OR: 1.85 (0.8, 4.1)	NR	No	NR	
		Naltrexone + disulfiram	AUD remission rate	NR	OR: 0.99 (0.2, 4.3)	NR	No	NR	
		Naltrexone + SSRI	AUD remission rate	NR	OR: 1.14 (0.4, 3.0)	NR	No	NR	
		SARI	AUD remission rate	NR	OR: 1.39 (0.4, 5.1)	NR	No	NR	
		NRI	AUD remission rate	NR	OR: 2.22 (0.4, 14.0)	NR	No	NR	
		SSRI	AUD remission rate	NR	OR: 2.13 (0.9, 4.8)	NR	No	NR	
		Tricyclic antidepressant	AUD remission rate	NR	OR: 1.55 (0.4, 5.5)	NR	No	NR	
Atenolol [103]	Alcohol dependence or AUD	Placebo	Continuous abstinence	84–365 days	OR: 0.85 (0.25, 2.95)	NA	NR	64	Very low
Bromocriptine [76]	AUD; comorbid depression/depressive symptoms	Control	AUD remission rate	NR	OR: 1.70 (0.50, 5.65) MD: 7.51 (-4.83, 19.91) SMD: -0.48 (-2.44, 1.48)	NR	No	NR	Low-moderate quality (all outcomes)
		Buspirone	AUD remission rate	NR	OR: 0.22 (0.03, 1.7)	NR	No	NR	
		Disulfiram	AUD remission rate	NR	OR: 0.07 (0.01, 0.5)	NR	No	NR	
		Lithium	AUD remission rate	NR	OR: 0.53 (0.01, 3.4)	NR	No	NR	
		Memantine	AUD remission rate	NR	OR: 0.32 (0.04, 2.7)	NR	No	NR	
		Naltrexone	AUD remission rate	NR	OR: 0.27 (0.05, 1.5)	NR	No	NR	
		Naltrexone + disulfiram	AUD remission rate	NR	OR: 0.14 (0.02, 1.2)	NR	No	NR	
		Naltrexone + SSRI	AUD remission rate	NR	OR: 0.16 (0.03, 0.96)	NR	No	NR	
		SARI	AUD remission rate	NR	OR: 0.20 (0.03, 1.4)	NR	No	NR	
		NRI	AUD remission rate	NR	OR: 0.32 (0.03, 3.3)	NR	No	NR	
		SSRI	AUD remission rate	NR	OR: 0.30 (0.05, 1.7)	NR	No	NR	
		Tricyclic antidepressant	AUD remission rate	NR	OR: 0.22 (0.03, 1.6)	NR	No	NR	
Buspirone [76]	AUD; comorbid depression/depressive symptoms	Control	AUD remission rate	NR	OR: 0.37 (0.07, 1.89) MD: 4.29 (-24.62, 33.18) SMD: -0.14 (-2.11, 1.83)	NR	No	NR	
		Disulfiram	AUD remission rate	NR	OR: 0.34 (0.07, 1.5)	NR	No	NR	
		Lithium	AUD remission rate	NR	OR: 2.44 (0.6, 11.0)	NR	No	NR	
		Memantine	AUD remission rate	NR	OR: 1.50 (0.2, 9.1)	NR	No	NR	
		Naltrexone	AUD remission rate	NR	OR: 0.82 (0.2, 3.5)	NR	No	NR	
		Naltrexone + disulfiram	AUD remission rate	NR	OR: 0.66 (0.1, 3.8)	NR	No	NR	
		Naltrexone + SSRI	AUD remission rate	NR	OR: 0.76 (0.2, 3.0)	NR	No	NR	
		SARI	AUD remission rate	NR	OR: 0.92 (0.2, 4.7)	NR	No	NR	
		NRI	AUD remission rate	NR	OR: 1.48 (0.2, 12.0)	NR	No	NR	
		SSRI	AUD remission rate	NR	OR: 1.41 (0.4, 5.0)	NR	No	NR	
		Tricyclic antidepressant	AUD remission rate	NR	OR: 0.97 (0.2, 5.1)	NR	No	NR	
Galantamine [103]	Alcohol dependence or AUD	Placebo	Continuous abstinence	84–365 days	OR: 0.31 (0.11, 0.87)	NR	NR	64	Low
Sodium oxybate (GHB) [103]	Alcohol dependence or AUD	Placebo	Continuous abstinence	84–365 days	OR: 2.31 (1.22, 4.36)	NR	NR	64	Very low
GHB 50 mg [81]	Alcohol dependent patients receiving therapy to prevent or to treat alcohol withdrawal symptom (AWS)	Naltrexone	Relapse to heavy drinking	3 months	RR: 3.23 (0.57, 18.33)	NR	NR	2	Very low
			Abstinence	3 months	RR: 2.59 (1.35, 4.98)	NR	NR	2	Very low
Lisuride [103]	Alcohol dependence or AUD	Placebo	Continuous abstinence	84–365 days	OR: 0.38 (0.13, 1.12)	NR	NR	64	Very low
Lithium [103]	Alcohol dependence or AUD	Placebo	Continuous abstinence	84–365 days	OR: 1.43 (0.39, 5.23)	NR	NR	64	Low
Lithium [76]	AUD; comorbid depression/depressive symptoms	Control	AUD remission rate	NR	OR: 0.70 (0.29, 1.52) MD: 4.98 (-7.23, 17.41) SMD: 0.01 (-1.35, 1.37)	NR	No	NR	Low-moderate quality (all outcomes)
		Memantine	AUD remission rate	NR	OR: 0.62 (0.1, 2.9)	NR	No	NR	
		Naltrexone	AUD remission rate	NR	OR: 0.51 (0.2, 2.1)	NR	No	NR	
		Naltrexone + disulfiram	AUD remission rate	NR	OR: 0.27 (0.1, 1.2)	NR	No	NR	
		Naltrexone + SSRI	AUD remission rate	NR	OR: 0.31 (0.1, 0.9)	NR	No	NR	
		SARI	AUD remission rate	NR	OR: 0.38 (0.1, 1.4)	NR	No	NR	
		NRI	AUD remission rate	NR	OR: 0.60 (0.1, 3.8)	NR	No	NR	
		SSRI	AUD remission rate	NR	OR: 0.58 (0.2, 1.4)	NR	No	NR	
		Tricyclic antidepressants	AUD remission rate	NR	OR: 0.42 (0.1, 1.5)	NR	No	NR	
Lithium, naltrexone/ disulfiram, imipramine [96]	Alcohol addiction and major depressive disorder	Placebo	Alcohol abstinence	NR	OR: 1.59 (0.84, 3.01)	32%	NR	3	NR
Memantine [76]	AUD; comorbid depression/depressive symptoms	Control	AUD remission rate	NR	OR: 1.10 (0.29, 4.60) SMD: -0.50 (-2.51, 1.50)	NR	No	NR	Low-moderate quality (all outcomes)
		Naltrexone	AUD remission rate	NR	OR: 0.82 (0.2, 3.5)	NR	No	NR	
		Naltrexone + disulfiram	AUD remission rate	NR	OR: 0.44 (0.1, 2.9)	NR	No	NR	
		Naltrexone + SSRI	AUD remission rate	NR	OR: 0.51 (0.1, 2.2)	NR	No	NR	
		SARI	AUD remission rate	NR	OR: 0.62 (0.1, 36)	NR	No	NR	
		NRI	AUD remission rate	NR	OR: 0.98 (0.1, 8.8)	NR	No	NR	
		SSRI	AUD remission rate	NR	OR: 0.94 (0.3, 3.5)	NR	No	NR	
		Tricyclic antidepressant	AUD remission rate	NR	OR: 0.69 (0.1, 4.0)	NR	No	NR	
Modafinil [103]	Alcohol dependence or AUD	Placebo	Continuous abstinence	84–365 days	OR: 2.48 (0.72, 8.53)	NR	NR	64	Low
Paroxetine [75]	Anxiety and comorbid alcohol use disorders	Placebo	Drinks per drinking day	12 – 16 weeks	MD: -2.42 (-4.97, 0.14)	0.0%	NR	2	Very low quality
			Proportion of days abstinent	12 – 16 weeks	MD: 0.08 (-0.26, 0.43)	68%	NR	2	Very low quality
Varenicline [87]	Patients people with problematic alcohol use	Placebo	Heavy drinking days	NR	SMD: -0.14 (-0.33, 0.05)	15%	No	5	NR
			Alcohol consumption (number of standard drinks over time)	NR	SMD: -0.37 (-0.66, -0.07)	49%	NR	3	High quality
**All pharmacological interventions**									
Pharmacological treatments [96]	Alcohol addiction and mood disorders	Placebo	Alcohol consumption	NR	SMD: -0.10 (-0.24, 0.04)	0%	NR	9	NR
	Alcohol addiction and mood disorders in bipolar disorder	Placebo	Alcohol consumption	NR	SMD: -0.07 (-0.25, 0.11)	0%	NR	4	NR
	Alcohol addiction and mood disorders in major depressive disorder	Placebo	Alcohol consumption	NR	SMD: -0.15 (-0.38, 0.08)	0%	NR	5	NR
	Alcohol addiction and major depressive disorder, no bipolar disorder	Placebo	Alcohol abstinence	NR	OR: 1.46 (1.02, 2.11)	0%	NR	8	NR
Pharmacotherapy [107]	AUD	Control	Abstinent rate, during treatment	≥ 12 weeks	OR: 1.18 (0.88, 1.60)	67.3%	NR	60	NR
			Abstinent rate, after treatment	≥ 12 weeks	OR: 0.68 (0.4, 1.16)	67.3%	NR	60	NR
		Pharmacotherapy + BI	Abstinent rate, during treatment	≥ 12 weeks	OR: 1.95 (1.18, 3.18)	67.3%	NR	60	NR
			Abstinent rate, after treatment	≥ 12 weeks	OR: 1.02 (0.50, 2.14)	67.3%	NR	60	NR
		Pharmacotherapy + psychotherapy	Abstinent rate, during treatment	≥ 12 weeks	OR: 1.49 (0.97, 2.34)	67.3%	NR	60	NR
			Abstinent rate, after treatment	≥ 12 weeks	OR: 0.93 (0.40, 2.21)	67.3%	NR	60	NR
		Psychotherapy	Abstinent rate, during treatment	≥ 12 weeks	OR: 1.27 (0.84, 1.96)	67.3%	NR	60	NR
			Abstinent rate, after treatment	≥ 12 weeks	OR: 1.44 (0.70, 3.01)	67.3%	NR	60	NR
		Psychotherapy + BI	Abstinent rate, during treatment	≥ 12 weeks	OR: 1.54 (0.72, 3.28)	67.3%	NR	60	NR
			Abstinent rate, after treatment	≥ 12 weeks	OR: 3.20 (1.08, 9.55)	67.3%	NR	60	NR
Combination interventions									
Naltrexone + disulfiram [76]	AUD; comorbid depression/depressive symptoms	Control	AUD remission rate	NR	OR: 2.60 (0.71, 10.15) MD: 10.72 (-6.11, 27.65) SMD: 0.30 (-1.37, 1.98)	NR	No	NR	Low-moderate quality (all outcomes)
		Naltrexone + SSRI	AUD remission rate	NR	OR: 1.15 (0.3, 5.1)	NR	No	NR	
		SARI	AUD remission rate	NR	OR: 1.40 (0.3, 8.0)	NR	No	NR	
		NRI	AUD remission rate	NR	OR: 2.25 (0.3, 19.0)	NR	No	NR	
		SSRI	AUD remission rate	NR	OR: 2.14 (2.5, 8.8)	NR	No	NR	
		Tricyclic antidepressant	AUD remission rate	NR	OR: 1.57 (0.3, 8.8)	NR	No	NR	
Naltrexone + SSRI [76]	AUD; comorbid depression/depressive symptoms	Control	AUD remission rate	NR	OR: 2.24 (1.15, 4.50) MD: 10.01 (-4.32, 24.42) SMD: -0.19 (-1.07, 0.68)	NR	No	NR	
		SARI	AUD remission rate	NR	OR: 1.22 (0.3, 4.4)	NR	No	NR	
		NRI	AUD remission rate	NR	OR: 1.94 (0.3, 12.0)	NR	No	NR	
		SSRI	AUD remission rate	NR	OR: 1.86 (1.0, 3.6)	NR	No	NR	
		Tricyclic antidepressant	AUD remission rate	NR	OR: 1.36 (0.4, 4.8)	NR	No	NR	
Acamprosate + nurse visit [103]	Alcohol dependence or AUD	Placebo	Continuous abstinence	84–365 days	OR: 4.59 (1.47, 14.36)	NR	NR	64	Very low
Acamprosate + Naltrexone [103]	Alcohol dependence or AUD	Placebo	Continuous abstinence	84–365 days	OR: 3.68 (1.5, 9.02)	NR	NR	64	Low
GHB + Escitalopram [103]	Alcohol dependence or AUD	Placebo	Continuous abstinence	84–365 days	OR: 5.13 (0.53, 49.92)	NR	NR	64	Low
GHB + Naltrexone [103]	Alcohol dependence or AUD	Placebo	Continuous abstinence	84–365 days	OR: 12.64 (2.77, 57.78)	NR	NR	64	Very low
Naltrexone + Escitalopram [103]	Alcohol dependence or AUD	Placebo	Continuous abstinence	84–365 days	OR: 2.57 (0.25, 25.85)	NR	NR	64	Low
Naltrexone + GHB + Escitalopram [103]	Alcohol dependence or AUD	Placebo	Continuous abstinence	84–365 days	OR: 25.65 (2.13, 309.46)	NR	NR	64	Low
Combination interventions (psychosocial and pharmacological)									
XR-naltrexone + psychosocial intervention [86]	AUD	Placebo + psychosocial intervention	Drinking days per month	< 6 weeks	MD: -2.00 (-3.39, -0.61)	0%	No	5	NR
XR-naltrexone + psychosocial intervention [86]	AUD	Placebo + psychosocial intervention	Heavy drinking days per month	< 6 weeks	MD: -1.16 (-2.1, -0.23)	0%	No	7	NR
Intensive perioperative cessation programme (disulfiram, chlordiazepoxide, motivational counselling, brief interview on alcohol intake, B vitamins) [106]	Hazardous drinkers undergoing all types of surgical procedures	Treatment as usual	Abstinence (self-report/ interview)	Min: 4 weeks Max: 3 months	RR: 8.22 (1.67, 40.44)	47%	NR	3	Moderate (GRADE)
Naltrexone (50 mg) plus psychotherapy [109]	Alcohol-dependent patients	Placebo plus psychotherapy	Percentage of drinking days	12–16 weeks	MD: -4.30 (-6.16, -2.44)	NR	NR	4	High
Naltrexone (50 mg) plus psychotherapy [109]	Alcohol-dependent patients	Placebo plus psychotherapy	Abstinence rate (AR)	12—16 weeks	OR: 1.46 (1.07, 2.00)	NR	Yes	9	High
Naltrexone (50 mg) plus psychotherapy [109]	Alcohol-dependent patients	Placebo plus psychotherapy	Number of drinks per drinking days	12–16 weeks	MD: -0.28 (-0.50, -0.07)	NR	NR	6	High
Naltrexone (50 mg) plus psychotherapy [109]	Alcohol-dependent patients	Placebo plus psychotherapy	Abstinence rate (AR)	24—36 weeks	Not significant	NR	NR	2	High
Naltrexone (50 mg) plus psychotherapy [109]	Alcohol-dependent patients	Placebo plus psychotherapy	Relapse rate	12—16 weeks	OR: 0.48 (0.36, 0.64)	NR	Yes	13	High
Pharmacotherapy + psychotherapy [107]	AUD	Control	Abstinent rate, during treatment	≥ 12 weeks	OR: 1.76 (1.17, 2.73)	67.3%	NR	1	NR
Pharmacotherapy + psychotherapy [107]	AUD	Control	Abstinent rate, after treatment	≥ 12 weeks	OR: 0.63 (0.32, 1.31)	67.3%	NR	0	NR
Phamacotherapy + Psychotherapy [107]	AUD	Psychotherapy	Abstinent rate, during treatment	≥ 12 weeks	OR: 0.86 (0.63, 1.14)	67.3%	NR	25	NR
Phamacotherapy + Psychotherapy [107]	AUD	Psychotherapy	Abstinent rate, after treatment	≥ 12 weeks	OR: 1.56 (0.94, 2.56)	67.3%	NR	4	NR
Phamacotherapy + Psychotherapy [107]	AUD	Psychotherapy + BI	Abstinent rate, during treatment	≥ 12 weeks	OR: 1.03 (0.48, 2.19)	67.3%	NR	1	NR
Phamacotherapy + Psychotherapy [107]	AUD	Psychotherapy + BI	Abstinent rate, after treatment	≥ 12 weeks	OR: 3.47 (1.26, 9.52)	67.3%	NR	0	NR

*ASI* Alcohol severity index, *AUD* Alcohol use disorder, *AUDIT* AUD identification test, *BI* Brief intervention, *CI* Confidence interval, *CM* Contingency management, *GHB* Gamma hydroxybutyric acid, *MD* Mean difference, *NMD* Network mean difference, *NR* Not reported, *NRI* Noradrenaline reuptake inhibitor, *OR* Odds ratio, *RAPI* Rutgers Alcohol Problems Index, *RCT* Randomized controlled trial, *RR* Relative risk, *SARI* Serotonin receptor antagonist reuptake inhibitor, *SMD* Standard mean difference, *SSRI* Selective serotonin reuptake inhibitor, *WMD* Weighted mean difference, *XR* Extended release

**Table 10:** Certainty in evidence (GRADE) for pharmacological interventions

		**Anticonvulsants**	**Antidepressants**	**Antipsychotics**	**Disulfiram**	**Baclofen**	**Acamprosate**	**Nalmefene**	**Naltrexone**
Risk of bias	Meta-analyses (AMSTAR)	Low	Low	Very low	Very low	Low	Very low	Moderate	Low
	RCTs	Low	Low	Low	Low	Low	Moderate	Moderate	Low
Inconsistency	Meta-analyses	Consistent	Consistent	Consistent	Inconsistent	Inconsistent	Consistent	Consistent (overlap in RCTs)	Very inconsistent
	Heterogeneity of RCTs	High	Low	High	High	Moderate	High	Low	Moderate
Indirectness		Applicable	Applicable	Applicable	Applicable	Applicable	Applicable	Applicable	Applicable
Imprecision	Confidence interval	Moderate	Wide	Very wide	Wide	Wide	Moderate	Small	Wide
	Sample size	Thousands	Less than 100	Hundreds	Thousands	Hundreds	Thousands	Hundreds	Thousands
Small study effect (number of studies)		Yes (1 topiramate), No (1), NR (3)	Yes (1), No (1), NR (2)	No (1), NR (2)	No (1), Yes (1), NR (3)	No (3), Yes (2), NR (2)	Yes (2), No (3), NR (5)	Yes (1), NR (1)	Yes (2), No (1), NR (5)
Other		NA	Effect only in studies with publication bias	Only quetiapine has large effect size	NA	NA	NA	NA	NA
**Overall**		**Very low**	**Very low**	**Very low**	**Very low**	**Very low**	**Low**	**Low**	**Very low**

Five studies reported outcomes for anticonvulsants compared to placebo in individuals with AUD or alcohol dependency [73, 80, 89, 90, 103]. A moderate quality review considering all anticonvulsants found a small but significant effect after 12 weeks in terms of drinks per drinking day (MD -1.49, 95% CI -2.32 to -0.65) and mean heavy drinking (MD -0.35, 95% CI -0.51 to -0.19), but the effect for abstinence at 15 weeks was not significant (MD 1.21, 95% CI 0.97 to 1.52) [90]. A low quality network meta-analysis found no significant effect on continuous abstinence (although confidence intervals were very wide) for carbamazepine (OR 0.55, 95% CI 0.08, 3.90), levetiracetam (OR 1.03, 95% CI 0.46 to 2.34), oxcarbazepine (OR 2.46, 95% CI 0.91 to 6.61) or pregabalin (OR 1.97, 95% CI 0.58 to 6.47) [103]. Two low quality reviews of gabapentin with high heterogeneity found no significant effect across measures of alcohol consumption, heavy drinking, and abstinence, except for a very small effect on percentage of heavy drinking days in both studies (Hedge’s g 0.55, 95% CI 0.01, 1.08 [73]; MD -0.64, 95% CI -0.64, 95% CI -1.22 to -0.06 [80]). For topiramate, one low quality network meta-analysis found a significant increase in continuous abstinence at 84–365 days (OR 1.88, 95% CI 1.06 to 3.34) [103] and a high quality network meta-analysis found a significant reduction in total consumption at 3–52 weeks (SMD -0.77, 95% CI -1.12 to -0.42; NMD -0.79, 95% CI -1.21 to -0.36), as well as for drinking days and heavy drinking days, but drinks per drinking day and mortality outcomes were not significant, and the study noted very low quality of RCTs alongside presence of publication bias [89]. Certainty in the evidence for anticonvulsants was downgraded to very low, due to high risk of bias, high heterogeneity in RCTs, low precision, and presence of publication bias.

Four studies evaluated antidepressants for AUD, of which three considered individuals with comorbid depression [70, 76, 96]. No significant effect was observed on alcohol abstinence among people with comorbid major depressive disorder for nefazodone compared with placebo (OR 2.18, 95% CI 0.68 to 7.07) [96]; AUD remission rate compared to control among individuals with comorbid depression for mirtazapine (SMD -0.78, 95% CI -1.69 to 0.13) [76], SARI (OR 1.85, 95% CI 0.62 to 5.66) [76], tricyclic antidepressants (OR 1.65, 95% CI 0.57 to 4.73) [76]; or continuous abstinence when compared with placebo at 84–365 days for citalopram or escitalopram (OR 1.03, 95% CI 0.33 to 3.16), nefazodone (OR 0.57, 95% CI 0.19 to 1.76), tianeptine (OR 1.22, 95% CI 0.58 to 2.57), or tiapride (OR 0.56, 95% CI 0.3 to 1.05) [103]. For NRI, a very low quality meta-analysis found a reduction in AUD remission rate compared to control in individuals with comorbid depression for SMD (-2.44, 95% CI -3.53 to -1.36) but not OR (1.15, 95% CI 0.21 to 6.31) [76]. All studies considering any type of SSRI were conducted among people with comorbid depression. Whilst a moderate quality study found no significant effect on abstinence (OR 1.26, 95% CI 0.06 to 2.56) [96] and a very low quality network meta-analysis found no significant effect on AUD remission rate (OR 1.21, 95% CI 0.78 to 1.92) [76], a low quality study reporting publication bias reported a significant effect on drinks per drinking day (MD -1.42, 95% CI -2.58 to -0.26) and number of abstinent participants (RR 1.66, 95% CI 1.02 to 2.68) [70]. The same study identified a reduction in drinks per drinking day for 5-HT2 antagonists compared to placebo (MD -1.06, 95% CI-2.00 to -0.11) [70]. Another study reviewing the effect of specific SSRIs in promoting abstinence did not identify a significant effect for fluoxetine (OR 2.97, 95% CI 0.97 to 9.05), fluvoxamine (OR 1.03, 95% CI 0.57 to 1.88), or trazodone (OR 0.61, 95% CI 0.2 to 1.84) [103]. Certainty in the evidence for antidepressants was rated as very low due to low methodological quality, imprecision and publication bias.

For antipsychotics, three studies reported outcomes [76, 79, 103]. No significant effect was found compared to placebo for preventing relapse with aripiprazole (RR 1.07, 95% CI 0.92 to 1.24), quetiapine (RR 0.87, 95% CI 0.65 to 1.17), or tiapride (RR 1.07, 95% CI 0.67 to 1.71) [79], nor for maintaining abstinence with amisulpride (OR 0.39, 95% CI 0.09 to 1.64) or aripiprazole (OR 1.49, 95% CI to 0.43 to 5.18) [103]. Flupentixol was found to be inferior to placebo in maintaining abstinence in a network meta-analysis (OR 0.44, 95% CI 0.2 to 0.95), whereas quetiapine had a very large positive effect (OR 6,75, 95% CI 1.2 to 38.05) [103]. It should be noted that total number of participants for both flupentixol and quetiapine was below 100. For antipsychotics as a whole, a network meta-analysis of AUD remission rate among people with comorbid depression found no significant effect compared with control (OR 0.97, 95% CI 0.30 to 3.22) and suggested that antipsychotics are inferior to disulfiram (OR 0.19, 95% CI 0.04 to 0.9) [76]. However, whilst there was no effect on relapse, another review found a significant improvement in ratio of abstinent to drinking days when compared with placebo at 3–12 months follow-up (RR 0.17, 95% CI 0.01 to 0.33) [79]. Certainty in the evidence for antipsychotics was rated as very low due to risk of bias, heterogeneity among RCTs and poor precision.

Of the five studies on disulfiram, three very low qualities found a significant effect, for increase in AUD remission rate among people with comorbid depression compared with control (OR 5.00, 95% CI 1.97 to 12.95), acamprosate, antipsychotics, bromocriptine, lithium, naltrexone, and SSRI [76]; abstinence at 12 months (OR 2.24, 95% CI 1.69 to 2.27, compared to other or no treatment), with greater improvement for supervised administration of disulfiram (OR 3.89, 95% CI 2.66 to 5.58 [78]; and combined measures of alcohol use at 8–52 weeks (Hedge’s g 0.58, 95% CI 0.35 to 0.82), although the latter study identified potential publication bias [94]. In contrast, one low quality network meta-analysis found no effect on abstinence compared to placebo at 84–365 days (OR 0.93, 95% CI 0.48 to 1.79) [103] and a moderate quality review of moderate quality RCTs found no effect on return to drinking compared with placebo or another medication (risk difference -0.04, 95% CI -0.11 to 0.03) [77]. Evidence for disulfiram was rated as very low because of a high level of inconsistency and risk of bias in many reviews.

There was considerable variation in outcomes among studies comparing baclofen to control. One very low quality study on people with comorbid anxiety and depression identified no significant reduction in heavy drinking days (SMD -0.26, 95% CI -0.68 to 0.15) or improvement in abstinent days (SMD 0.03, 95% CI -0.10 to 0.15) [91]. Conversely, one low quality network meta-analysis with a very small sample identified a significant improvement in abstinence (OR 4.63, 95% CI 1.00 to 21.48) [103] and a moderate quality review with high quality RCTs found a significant decrease in amount of drinking (SMD 0.28, 95% CI 0.00 to 0.56) [71]. A very low quality network meta-analysis among people with comorbid depression did not find a significant effect in AUD remission rate aside from mean difference compared to control (the odds ratio and standardised mean difference measures were not significant) [76]. A low quality review found a significant increase in percentage abstinent days at 1–3 months (RR 2.79, 95% CI 1.79 to 4.34) but not for mean abstinent days (SMD 3.69, 95% CI 0.74 to 8.11) [82], while a high quality review found a small but significant effect for drinks per drinking day (MD 1.55, 95% CI 1.32 to 1.77) but not relapse (RR 0.88, 95% CI 0.74 to 1.04), percentage of heavy drinking days (MD 0.25, 95% CI -1.25 to 1.76), or percentage days abstinent (MD 0.39, 95% CI -11.51 to 12.29) [85], and a high quality network meta-analysis with a very small sample and suspected publication bias identified a significant reduction in total alcohol consumption at 3–52 weeks follow up (SMD -1.00, 95% CI -1.80 to -0.19; NMD -1.00, 95% CI -1.86 to -0.13), but not for non-drinking days or heavy drinking days [89]. Overall evidence for baclofen was graded as very low due to the inconsistency, small sample size, and presence of publication bias in many reviews.

No reviews were identified for benzodiazepines that met our inclusion criteria.

Nine reviews assessed the efficacy of acamprosate [72, 74, 76, 77, 83, 84, 92, 95, 103]. Seven of the studies showed acamprosate to be effective relative to placebo for promoting abstinence (OR 1.88, 95% CI 1.57 to 2.25, 1–12 months [72]; RR 0.83, 95% CI 0.78 to 0.89, 6 months [74]; RR 1.95, 95% CI 1.58to 2.42, 12 months [83]; OR 1.86, 95% CI 1.49 to 2.33, 84–365 days [103]; OR 1.87, 95% CI 1.57 to 2.23 [84]), cumulative abstinence duration (WMD 26.55, 95% CI 17.56 to 35.54, 3–12 months [72]; RR 1.11, 95% CI 1.01 to 1.21, 12 months [95]; MD 10.94, 95% CI 5.08 to 16.81, 12 months [92]), and mean percentage abstinent days (MD 10.38, 95% CI 7.10 to 13.65) [84]. A moderate quality review of acamprosate compared to either placebo or another medication found a small but significant effect for return to any drinking (risk difference -0.09, 95% CI -0.14 to -0.04) and percentage drinking days (WMD -8.8, 95% CI -12.8 to -4.8) but not for return for heavy drinking (risk difference -0.01, 95% CI -0.04 to 0.03) [77]. A very low quality network meta-analysis of AUD remission rates among individuals with comorbid depression did not find a significant effect relative to control (OR 1.66, 95% CI 0.89 to 3.05) and found acamprosate to be inferior to disulfiram (OR 0.33, 95% CI 0.1 to 0.9) [76]. Acamprosate combined with a nurse visit showed a significant improvement in continuous abstinence as compared to placebo (OR 4.59, 95% CI 1.47 to 14.36) [103]. Although methodological quality of reviews was low and two studies identified publication bias [72, 74], the evidence was graded as low certainty due to good agreement across a large number of studies, each with thousands of participants.

Reviews evaluated two opioid antagonists: nalmefene and naltrexone. Both studies comparing nalmefene to placebo found a small but significant decrease in alcohol consumption (SMD -0.2, 95% CI -0.3 to -0.1 [88]; SMD -0.19, 95% CI -0.29 to -0.10 [89]) and heavy drinking days (MD -1.65, 95% CI -2.41 to -0.89 [88]; SMD -0.22, 95% CI -0.32 to -0.12 [89]). However, only one of the studies found a significant decrease in mortality and no effect was found on non-drinking days (SMD 0.09, 95% CI-0.01 to 0.19) [89]. Certainty in the evidence for nalmefene was graded as low, since there was a small sample size and presence of publication bias.

Among the nine reviews of naltrexone, three network meta-analyses found no significant effect on abstinence (OR 1.36, 95% CI 0.97 to 1.91) [103], AUD remission rate (OR 1.38, 95% CI 0.88 to 2.18), or across five outcomes of alcohol use [89]. A low quality study found naltrexone to be significantly inferior to GHB (OR 2.31, 95% CI 1.22 to 4.36) [81]. Four studies did find a significant effect across mean percentage of drinking days (MD -2.8, 95% CI -5.8 to -0.2) and abstinence rate (RR 1.28, 95% CI 1.08 to 1.52) [97]; drinking days (MD-3.89, 95% CI -5.75 to -2.04) and heavy drinking days (MD -3.25, 95% CI -5.51 to -0.99) [93]; cumulative abstinence duration (RR 1.23, 95% CI 1.00 to 1.78) and relapse rate (RR 1.2, 95% CI 1.17 to 1.47) [95];and five measures of alcohol use, but not abstinence rate (OR 1.26, 95% CI 0.97 to 1.64) [72]. A moderate quality review found a small but significant effect on 50 mg oral naltrexone for five alcohol use outcomes, but most outcomes were not significant for 100 mg oral naltrexone and naltrexone injection, aside from percentage heavy drinking days (WMD -3.1, 95% CI -5.8 to -0.3 and WMD -4.6, 95% CI -8.5 to -0.56 respectively) [77]. Certainty of evidence was rated as very low due to low methodological quality, inconsistency of results and presence of publication bias.

Among other pharmacological interventions, a low quality meta-analysis found no significant effect compared to placebo on continuous abstinence for atenolol (OR 0.85, 95% CI 0.25 to 2.95), lisuride (OR 0.38, 95% CI 0.13 to 1.12), or modafinil (OR 2.48, 95% CI 0.72 to 8.53) [103]. The same study found galantamine to be inferior to placebo in promoting abstinence (OR 0.31, 95% CI 0.11 to 0.87). A very low quality network meta-analysis on AUD remission rate among people with comorbid depression did not find a significant effect relative to control of bromocriptine (OR 1.70, 95% CI 0.50 to 5.65), buspirone (OR 0.37, 95% CI 0.07 to 1.89), or memantine (OR 0.70, 95% CI 0.29 to 1.52) [76]. The same meta-analysis found bromocriptine to be inferior to disulfiram (OR 0.07, 95% CI 0.01 to 0.5) and naltrexone combined with SSRI (OR 0.16, 95% CI 0.03 to 0.96). Neither network meta-analysis identified a significant effect of lithium (AUD remission rate among individuals with comorbid depression OR 0.70, 95% CI 0.29 to 1.52 [76]; continuous abstinence OR 1.43, 95% CI 0.39 to 5.23 [103]). A comparison of paroxetine with placebo among patients with comorbid anxiety did not find a significant effect of paroxetine for reducing drinks per drinking day (MD –2.42, 95% CI –4.97 to 0.14) or proportion of days abstinent (MD 0.08, 95% CI –0.26 to 0.43) [75]. Of the studies on GHB, the effect on relapse to heavy drinking compared to naltrexone was not significant (RR 3.23, 95% CI 0.57 to 18.33) but there was a significant improvement in abstinence compared to naltrexone at 3 months (2.59, 95% CI 1.35 to 4.98) [81] and a significant effect on abstinence relative to placebo at 84–365 days (OR 2.31, 95% CI 1.22 to 4.36) [103]. For alpha blockers, a very low quality study showed significant reduction in drinks per day or week at 6–13 weeks follow-up (SMD –0.32, 95% CI –0.56 to –0.07) but not for heavy drinking days (SMD –0.44, 95% CI –0.94 to 0.06) [98]. Similarly, evidence from was inconclusive for antiepileptics, which showed a significant improvement in AUD remission rate among individuals with comorbid depression when compared to control, bromocriptine, or lithium in a network meta-analysis of odds ratios, but not when compared to control using standardised mean difference (SMD –0.70, 95% CI –2.05 to 0.65) [76], and for varenicline, which showed a significant effect for alcohol consumption (SMD –0.37, 95% CI –0.66 to –0.07) but not heavy drinking days (SMD -0.14, 95% CI –0.33 to 0.05) [87]. Certainty in the evidence for each of the other pharmacological interventions was rated as very low, due to high risk of bias and imprecision in many studies, resulting from very wide confidence intervals and/or sample sizes less than 100 (Supplement 6).

Two studies evaluated pharmacological interventions as a whole. A review of pharmacological treatments for people with concurrent bipolar disorder or depression found no effect on alcohol consumption (SMD –0.10, 95% CI –0.24 to 0.04) but did find an improvement in abstinence (OR 1.46, 95% CI 1.02 to 2.11) [96], unlike a network meta-analysis of pharmacotherapy for AUD which found no improvement in abstinence rate (OR 0.68, 95% CI 0.4 to 1.16, after treatment) [107]. Due to the variation in efficacy across different types of pharmacological intervention, any analysis of pharmacological interventions is likely to be very dependent on the specific interventions included.

Two network meta-analyses assessed combinations of pharmacological interventions [76, 103]. There were six combinations including naltrexone. Among individuals with comorbid AUD and depression, there was no significant effect on AUD remission rate as compared to control for naltrexone combined with disulfiram (OR 2.60, 95% CI 0.71 to 10.15) and although naltrexone combined with SSRI showed a significant effect compared to control (OR 2.24, 95% CI 1.15 to 4.50) and SSRI (OR 1.86, 95% CI 1.0 to 3.6) when odds ratios were considered, standardised mean difference was not significant (SMD –0.19, 95% CI –1.07 to 0.68) and there was no significant effect compared with naltrexone alone (OR 0.62, 95% CI 0.3 to 1.3), although confidence intervals were very wide [76]. Among people with alcohol dependence or AUD, there was no significant change in continuous abstinence relative to placebo for naltrexone combined with escitalopram (OR 2.57, 95% CI 0.25 to 25.85), but there was a significant effect for naltrexone combined with acamprosate (OR 3.68, 95% CI 1.5 to 9.02), GHB (OR 12.64, 95% CI 2.77 to 57.78), and GHB with escitalopram (OR 25.65, 95% CI 2.13 to 309.46) [103]. The effect on continuous abstinence of GHB combined with escitalopram was not significant (OR 5.13, 95% CI 0.53 to 49.92) [103]. Certainty in the evidence for all combinations of pharmacological interventions was graded as very low due to considerable imprecision and low methodological quality of meta-analyses and RCTs (Supplement 6).

Three reviews considered combinations of psychosocial and pharmacological interventions. Extended-release naltrexone combined with psychosocial interventions had a small but significant effect up to 6 weeks in reducing drinking days per month (MD -2.00, 95% CI -3.39 to -0.61) and heavy drinking days per month (MD -1.16, 95% CI -2.1 to -0.23) among patients with AUD, compared to psychosocial interventions alone [86]. Naltrexone (50 mg) combined with psychotherapy was found to be more effective than placebo and psychotherapy in reducing alcohol consumption and improving abstinence in alcohol dependent patients at 3–4 months (OR 1.46, 95% CI 1.07 to 2.00), but the effect was not significant at 6–9 months follow-up [109]. The body of evidence for naltrexone combined with psychosocial interventions was rated as very low. One review reported outcomes for intensive perioperative cessation programmes, which provided disulfiram, chlordiazepoxide, motivational counselling, brief interview, and B vitamins to hazardous drinkers undergoing surgical procedures [106]. There was a high improvement in self-reported abstinence between 1 and 3 months (RR 8.22, 95% CI 1.67 to 40.44) [106]. Although there was high methodological quality, as well as large effect size, the meta-analysis only incorporated 70 participants, so the certainty was downgraded to low (Supplement 6).

### Miscellaneous interventions

The literature search yielded one very low quality study on physical activity and one very low quality study on bibliotherapy (Table 11). The meta-analysis of bibliotherapy (intervention in a written format) found a mean weighted effect size for alcohol consumption of 0.21 when compared with no intervention, but confidence intervals were not reported [99]. Certainty of evidence was graded as very low due to poor methodological quality of a single study with heterogeneous results. For physical activity, no significant effect was found on abstinence among individuals with AUD or those seeking treatment for alcohol use (RR 1.56, 95% CI 0.78 to 3.14) [100]. The certainty of evidence was graded as very low due to poor precision and very low quality RCTs.

**Table 11:** Summary results of included systematic reviews, for miscellaneous interventions

**Intervention**	**Population**	**Comparator**	**Outcome**	**Timeframe**	**Effect size** (method: effect (95% CI))	**I**^**2**^	**Publication bias**	**Number of RCTs**	**Quality assessment of RCTs**
Bibliotherapy (any therapeutic intervention presented in a written format, designed to be read and implemented by the client (e.g. brochures, self-help manuals or books) [99]	Patients Identified as “At-Risk” Through Screening	No intervention	Alcohol consumption	2–12 months	MW: 0.21	NR	NR	9	High
	Patients Identified as “At-Risk” Through Screening	More extensive treatment	alcohol consumption	2–12 months	MW: -0.1	NR	NR	3	High
Physical activity [100]	Individuals diagnosed with AUD and/or seeking treatment for alcohol use	Alternative non-physical activity, usual care, no intervention	Abstinence from alcohol	1 month to 3 years	RR: 1.56 (0.78, 3.14)	67.1%	NR	2	Very low

*AUD* Alcohol use disorder, *CI* Confidence interval, *MW* Mean weight, *NR* Not reported, *RCT* Randomised controlled trial, *RR* Relative risk

### Comparison against clinical practice guidelines

A summary of results from the umbrella review are presented in Table 12. For screening, brief interventions and referral to treatment, the NICE clinical practice guidelines (CPG) recommend that all individuals identified with harmful drinking or dependence receive motivational interviewing [24]. Whilst our review found motivational interviewing (and brief motivational interviewing) to be effective, we identified a greater level of variation than for brief counselling or general brief interventions, and no evidence to support delivery of motivational interviews to those with alcohol dependence. However, it should be noted that findings from our review considered motivational interviewing delivered independently, not at the start of treatment initiation as with the NICE CPG.

**Table 12:** Summary of findings table

Intervention category	Summary of findings	Certainty in findings (GRADE)
**Research question 1:** interventions for individuals at risk of, or with, hazardous/harmful drinking		
Screening, brief intervention and referral to treatment	**Brief interventions** show a small effect in the mid to long term (6 to 12 months). More than one session should be provided, although further research is required on the optimal duration and frequency of sessions. There does not seem to be a significant difference of effect among different sub-groups, but further research is required **Brief counselling** interventions show high effectiveness in the mid-long term (up to 12 months). **Brief motivational interviewing** effect is highly variable and likely to depend on context and delivery. There is insufficient evidence to support **social norms** interventions	Low
	No evidence identified for referral mechanisms	NA
Psychosocial interventions	**Cognitive behavioural therapy** delivered by lay health workers may be effective in the mid-term (up to 6 months). No evidence for other settings	Very low
	No evidence identified for **contingency management**	NA
	No evidence identified for **community reinforcement approach**	NA
	**Motivational interviewing** shows a small reduction in consumption in the mid-term (up to 6 months)	Low
	**Family-oriented approaches** has no evidence of effect in school-aged children. No evidence identified for other groups	Very low
	**Mentoring** may significantly reduce alcohol use among children and adolescents over the long-term (over 1 year)	Moderate
	**Forming implementation intentions** may have a large short-term effect	Moderate
Digital interventions	Digital interventions (web, computer, mobile-based) show non-inferiority to face-to-face interventions	Very low
Pre-operative alcohol cessation programme	Intensive pre-operative cessation programmes (including psychosocial interventions and disulfiram) show a significant improvement in abstinence	Low
**Research question 2:** interventions for individuals with alcohol dependence or alcohol use disorder		
Screening, brief intervention and referral to treatment	No effect has been shown for brief interventions	Very low
	No evidence identified for referral to treatment	NA
Psychosocial interventions	**Cognitive behavioural therapy** shows no evidence of effect	Very low
	For **contingency management**, imprecision in the evidence is too great to draw any conclusion. Further research is required	Very low
	**Community reinforcement approach** shows a large, long-term effect	Low
	No evidence identified for **motivational interview**	NA
	No evidence identified for **family-oriented approaches**	NA
	No effect shown for **coping skills training**	Very low
	No effect shown for **cue exposure therapy**	Very low
	Abstinence-based strategies are likely to be more effective than moderation based strategies (controlled drinking)	Low
	**Naltrexone combined with psychosocial interventions** show an improved effect as compared with psychosocial interventions alone	Very low
Digital interventions	Insufficient evidence to draw meaningful conclusions	Very low
Pharmacological interventions	**Anticonvulsants** show no evidence of effectiveness, with the exception of topiramate for which there may be an effect and further research is required	Very low
	**Antidepressants** show no evidence of effectiveness, with the exception of SSRI for which the evidence is inconclusive	Very low
	**Antipsychotics** show no evidence of effectiveness, with the exception of quetiapine for which there may be an effect and further research is required	Very low
	The evidence for **disulfiram** is inconclusive	Very low
	The evidence for **baclofen** is inconclusive	Very low
	No evidence identified for **benzodiazepines**	NA
	**Acamprosate** shows good evidence of effectiveness up to 12 months	Low
	**Nalmefene** may be effective in reducing alcohol consumption	Low
	Evidence for **naltrexone** is inconclusive	Very low
	**GHB** may significantly improve abstinence, but further research is warranted	Very low
	No effect has been shown for **atenolol, bromocriptine, buspirone, galantamine, lisuride, lithium, memantine, modafinil**, or **paroxetine**	Very low
	Evidence is inconclusive for **alpha blockers, antiepileptics**, and **varenicline**	Very low
Combinations of pharmacological interventions	No effect has been shown for **naltrexone + SSRI**, **naltrexone + disulfiram**, **naltrexone + escitalopram**, or **GHB + escitalopram**	Very low
	There is evidence of an effect for **naltrexone + acamprosate**, but it is unclear whether the effect is superior to acamprosate alone	Very low
	There is evidence of an effect for **naltrexone + GHB** and **naltrexone + GHB + escitalopram**, but it is unclear whether the effect is superior to GHB alone	Very low
Miscellaneous interventions	**Physical activity** has not shown any significant effect when delivered alone	Very low

*GHB* Gamma-hydroxybutyric acid, *NA* Not applicable, *SSRI* Selective serotonin reuptake inhibitors

For harmful but not moderately or severely dependent drinkers, the NICE CPG recommends cognitive behavioural therapy, behavioural therapies, social network, or environment-based therapies for adults, with couples-based therapy provided when relevant, and cognitive behavioural therapy for children, potentially combined with family-based therapy in the presence of comorbidities or limited social support. CPGs from Australia, Canada, Germany, and the USA equally highlighted the importance of behavioural therapies in harmful drinkers [25, 26, 27, 28, 29]. In line with our findings, the NICE CPG recommends treating alcohol use and comorbidities separately. In our review we identified very limited evidence on the effect of cognitive behavioural therapy and no studies on social network interventions among this population. Furthermore, the only review identified for family-based approaches in children found no evidence of the effectiveness of this intervention, and from our review mentoring showed the greatest promise in addressing alcohol use among this population of adolescents and children. The umbrella review found non-inferiority of digital interventions, but this was not discussed in any of the CPGs.

For moderately or severely dependent individuals, the NICE CPG recommends focussing on a goal of abstinence, which is in line with findings from our review and guidelines from Australia, Canada, Germany, and the USA. The CPGs recommend the provision of either acamprosate or naltrexone in combination with psychosocial interventions, after assisted withdrawal with benzodiazepines and concurrent psychosocial support has taken place. NICE explicitly recommends against the use of GHB or antidepressants, including SSRIs. While the umbrella review showed good effectiveness of acamprosate, we found no outcomes for acamprosate delivered in combination with psychosocial interventions. Moreover, our review found conflicting evidence to support the use of naltrexone and suggested that GHB merits further research and review. No study identified in our review examined withdrawal.

## Discussion

To our knowledge, this study is the first umbrella review to provide an overview of all available interventions to address harmful alcohol use and dependence. This review demonstrates an approach by which to summarise evidence across a clinical area in settings with limited resources for health technology assessment, in order to prioritise interventions for further assessment in UHC policy decisions.

We examined the effectiveness of: (1) interventions for non-dependent individuals either experiencing or at risk of harmful drinking; and (2) interventions for individuals with alcohol use disorder. For our first research question on harmful alcohol use, brief interventions showed a small but significant effect up to 12 months. In general, brief interventions consisting of a single session, however, did not show a significant effect. Further investigation is required on the relationship between efficacy and number/duration of sessions. This finding is consistent with two umbrella reviews of brief interventions, which found a moderate effect on alcohol consumption but noted the need for further research on sub-group differences and the optimum length and frequency of sessions [116, 117]. Although brief interventions have been shown to be effective, it will be important to consider appropriate settings in which to conduct opportunistic screening, as the additional workload for personnel should be balanced against potential health gains [30]. Findings from this review suggested that provision of screening and brief interventions in a digital format could be as effective as face-to-face interventions, which aligned with the conclusions from a review of systematic reviews of computer-based interventions for problematic alcohol use [118]. This finding could provide an opportunity to extend the reach of screening and brief intervention services without contributing to the burnout of personnel. However, operational research would be required to identify strategies to reach groups with traditionally lower use of digital technologies, such as the elderly, people with disabilities, and socio-economically disadvantaged groups [119].

We did not identify any meta-analyses on referral mechanisms to treatment, which may indicate a gap in the literature, but could also be an artefact of our inclusion criteria, which required studies to report outcomes on alcohol use or health outcomes. Among psychosocial interventions, there is evidence to suggest that cognitive behavioural therapy and motivational interviewing could slightly reduce alcohol consumption in the intermediate term (up to 6 months), but further review is required, especially since it has been highlighted that contextual and health systems factors may limit transferability of findings to LMICs [61]. Forming implementation intentions demonstrated a large effect after a 3-month follow-up, but further research is required on the durability of the response. Similarly, there was promising evidence to suggest that mentoring may be able to significantly reduce alcohol use among children and adolescents, with effects lasting beyond one year, but further research is warranted to consider the applicability of findings across settings and to identify best practice for implementation. The evidence identified in this review for contingency management came from two network meta-analyses with wide confidence intervals, making it difficult to draw conclusions on its effectiveness, and we identified no reviews on community reinforcement approaches.

For our second research question on interventions for alcohol use disorder, abstinence-based strategies were found to be more effective than controlled drinking in reducing alcohol consumption. The only non-pharmacological intervention to show a significant effect was social networking, for which there is low certainty evidence of a large, long-term benefit for maintaining abstinence. Among pharmacological interventions, acamprosate showed good evidence of effectiveness over a 12-month timeframe. Although there were many reviews identified for disulfiram, baclofen, and naltrexone, there was considerable inconsistency between meta-analyses and high heterogeneity across the RCTs in each study, making it difficult to draw meaningful conclusions. Further review is required to identify whether this heterogeneity could be arising from sub-group differences or different practice in delivering the intervention. Real-world evidence could provide large datasets of patients with different characteristics to partially address this question. The umbrella review also highlighted a potential benefit of nalmefene, topiramate, quetiapine, and GHB, although further evidence review is required. Overall, the review highlighted the need for more studies of combinations of psychosocial and pharmacological interventions, since combining naltrexone with psychosocial interventions showed an improvement relative to psychosocial interventions alone, but no evidence was found relative to providing naltrexone alone or for other drugs. Early health technology assessment and expected value of perfect information analysis may identify combinations of interventions for which RCTs should be conducted [120]. We did not identify any studies on recovery management, or long-term interventions to maintain abstinence in previously dependent individuals.

Although there was moderate agreement between our results and the NICE CPG (as well as CPGs from other countries), the comparison highlighted that our umbrella review, by limiting the focus to meta-analyses of RCTs, may not have captured the full body of evidence on appropriate pathways or combinations of interventions. However, it should be highlighted that this review did show non-inferiority of digital interventions, which was not mentioned in any of the other clinical practice guidelines, and may be important for implementation of interventions in resource-constrained settings. It is also important to note that beyond results from meta-analyses of clinical benefit, NICE guidelines incorporate inputs from a series of stakeholder consultations, as well as socio-ethical and economic considerations, which may explain some of the differences in findings. In parallel to conducting an umbrella review, it may be beneficial to conduct a review of clinical practice guidelines from internationally recognised institutions to supplement and compare with findings from the review.

There are a number of limitations to this umbrella review. Firstly, since we defined our search terms and inclusion criteria based on two broad research questions (as opposed to defining research questions from the clinical pathway), we inadvertently excluded reviews of referral to treatment and withdrawal, since these interventions are commonly measured by drop-out rates and measures of craving/alcohol withdrawal respectively, which fell outside the scope of our inclusion criteria. Secondly, to facilitate comparison across types of intervention, we restricted the inclusion criteria to meta-analyses of RCTs only; subsequently, we could only extract very limited information on the delivery of interventions, which meant that we were not able to assess whether variation in delivery led to some of the discordance in results. Although methodologically rigorous [15], restricting analysis to RCTs has been criticised for leading to pharmacological bias, particularly for mental health treatments, as psychological interventions are often influenced to a greater extent by relationships, trust, and socio-cultural context, thus harder to control [121–123]. This may explain the reason why we identified very few eligible studies for many types of psychosocial intervention. For example, although a Cochrane Review of mutual support groups exists [124], the results for RCTs were combined with quasi-randomised studies. We suspect this is also the reason that our review did not identify recovery management interventions, which aim to support recovered individuals over the long term. Since we had to balance the feasibility of conducting the review with overly-restricting the scope, basing our analysis on the WHO/UNODC framework was helpful to at least identify gaps in the evidence from our review.

A further two limitations arise from the studies included in this review. The first is the very limited number of RCTs within the meta-analyses that were conducted in LMICs. Appropriate interventions for the prevention and treatment of alcohol use disorder are expected to vary across different health and social care services and socio-cultural contexts. Whilst we do not expect that findings will be more transferable between LMICs than between HICs and LMICs, our findings highlight a gap in the contexts and healthcare settings in which intervention studies are conducted. Similarly, we found limited investigation into the reasons for high heterogeneity across or within RCTs. Beyond conducting trials outside of high income settings, we recommend that RCTs adhere to intervention standards such as the TIDieR checklist [125], and that both RCTs and meta-analyses make greater effort to investigate the reasons behind heterogeneity in results. The second limitation is the low certainty in most of our findings. Meta-analyses are already prone to bias because they pool studies of different quality (internal bias) and relevance (external bias) [126]; certainty in the evidence for most of our conclusions is low because of very low methodological quality of the meta-analyses themselves (brief interventions), limited relevance due to very specific target populations (psychosocial interventions), and the presence of publication bias (pharmacological interventions). This highlights the need for better adherence to reporting guidelines for systematic reviews and RCTs. In particular, we recommend that meta-analyses are more transparent in publishing a protocol before conducting the review and in detailing their search strategy, as well as investigating the potential impact of risk of bias and publication bias on results. As noted above, the considerable heterogeneity across RCTs and meta-analyses suggests that there is a need to shift the focus of research from the interventions that are effective to a better understanding of which interventions work for whom, at which time, and in which context.

Despite these limitations, we believe that this study has been successful in describing the landscape of interventions to prevent and treat alcohol use disorder. The umbrella review methodology allowed us to succinctly summarise a large body of literature using rigorous methods, which can be used as a starting point for benefit package policy discussions.

## Conclusion

For people with harmful drinking or at-risk of harmful drinking, brief interventions show a small effect over a 6–12 month period, but further research is required to identify the appropriate frequency/duration of sessions, appropriate settings in which to conduct screening and brief intervention, and when digital brief interventions may be more appropriate, before identifying the scenarios for which brief interventions are a good use of resources. We also found a significant long-term effect of mentoring in adolescents and children, as well as limited evidence of a small effect of CBT and motivational interviewing in all populations, but further research on transferability to other contexts outside of high-income settings is required. For people with alcohol use disorder, there was consistent evidence of an effect for social network approaches and for acamprosate. Further review of community reinforcement approaches, including social network approaches, is recommended, particularly to identify any differences in effectiveness or contextualisation required for different cultures and health system settings, above all in LMICs. There may potentially be a benefit of GHB, nalmefene, and quetiapine, but further research is required on this topic, as well as for combinations of pharmacological and psychological interventions. The umbrella review methodology provided a practical and rigorous approach by which to summarise the effectiveness of interventions across a whole clinical area, for a research question with a very broad scope, and use of established frameworks and CPGs was important to identify gaps in the literature.

### Registration and protocol

The protocol is registered in PROSPERO (CRD42021275471) and available as a pre-print [14]. The following five amendments were made to the original protocol. Firstly, to facilitate comparison of effect sizes, we restricted our analysis to systematic reviews with meta-analysis, whereas in the protocol it states that narrative systematic reviews would also be included. Secondly, we did not use a citation matrix to exclude systematic reviews with overlapping RCTs, since it was considered unnecessary given that the purpose of the review is to highlight interventions for further investigation in health benefit package decisions. Thirdly, we did not use elements of the TIDieR checklist [131] for data extraction, as during piloting of the data extraction form, it was found that systematic reviews included insufficient information on the methods of included RCTs. On a related note, since we extracted limited information on how the interventions were delivered, we were unable to evaluate the feasibility of delivering interventions in LMICs in our results. Finally, we did not detail methods to assess certainty in the body of evidence in the protocol but applied GRADE in this study.

## Supplementary Information

**Additional file 1.**

**Additional file 2.**

**Additional file 3.**

**Additional file 4.**

**Additional file 5.**

**Additional file 6.**

**Additional file 7.**

## Data Availability

The data extraction form and list of excluded studies are included as supplementary files.
