# Supplementary material for: Which interventions for alcohol use should be included in a universal healthcare benefit package? An umbrella review of targeted interventions to address harmful drinking and dependence
Source: BMC Public Health. 2023 Feb 23;23:382. doi: 10.1186/s12889-023-15152-6 (PMC9948368; doi:10.1186/s12889-023-15152-6)
Supplement: Supplementary file 3 — Additional file 3. [file 12889_2023_15152_MOESM3_ESM.docx]

**Supplement 3.** Search strategy.

**MEDLINE via PubMed**

*Concept 1: harmful alcohol use*

#1 alcohol-related disorders[MeSH Terms]

#2 drinking behavior[MeSH Terms]

#3 "alcohol use"[Title/Abstract]

#4 alcoholic*[Title/Abstract]

#5 alcoholism[Title/Abstract]

#6 alcohol[Title/Abstract] AND (drink*[Title/Abstract] OR intoxicat*[Title/Abstract] OR abus*[Title/Abstract] OR misus*[Title/Abstract] OR addict*[Title/Abstract] OR depend*[Title/Abstract] OR disorder*[Title/Abstract] OR risk*[Title/Abstract] OR consum*[Title/Abstract] OR withdraw*[Title/Abstract] OR detox*[Title/Abstract] OR treat*[Title/Abstract] OR therap*[Title/Abstract] OR excess*[Title/Abstract] OR reduc*[Title/Abstract] OR cessation[Title/Abstract] OR intervention*[Title/Abstract] OR abstain[Title/Abstract] OR abstinence[Title/Abstract] OR sober[Title/Abstract] OR problem*[Title/Abstract])

#7 drink*[Title/Abstract] AND (excess*[Title/Abstract] OR heavy[Title/Abstract] OR heavily[Title/Abstract] OR hazard*[Title/Abstract] OR binge[Title/Abstract] OR harm[Title/Abstract] OR harmful[Title/Abstract] OR problem*[Title/Abstract])

#8 [#1 OR #2 OR #3 OR #4 OR #5 OR #6 OR #7]

*Concept 2: screening, brief intervention, referral*

#9 mass screening[MeSH Terms]

#10 diagnostic screening programs[MeSH Terms]

#11 counseling[MeSH Terms]

#12 interview, psychological[MeSH Terms]

#13 referral and consultation[MeSH Terms]

#14 screening[Title/Abstract]

#15 advice[Title/Abstract]

#16 referral[Title/Abstract]

#17 brief[Title/Abstract] AND (intervention*[Title/Abstract] OR therap*[Title/Abstract] OR interview*[Title/Abstract])

#18 minimal[Title/Abstract] AND (intervention*[Title/Abstract] OR therap*[Title/Abstract] OR interview*[Title/Abstract])

#19 early[Title/Abstract] AND (intervention*[Title/Abstract] OR therap*[Title/Abstract] OR interview*[Title/Abstract])

#20 motivat*[Title/Abstract] AND (intervention*[Title/Abstract] OR therap*[Title/Abstract] OR interview*[Title/Abstract])

#21 [#9 OR #10 OR #11 OR #12 OR #13 OR #14 OR #15 OR #16 OR #17 OR #18 OR #19 OR #20]

*Concept 3: psychosocial interventions*

#22 psychotherapy[MeSH Terms]

#23 motivation[MeSH Terms]

#24 self-help groups[MeSH Terms]

#25 counsel*[Title/Abstract]

#26 "contingency management"[Title/Abstract]

#27 "community reinforcement"[Title/Abstract]

#28 psychotherap*[Title/Abstract] OR psychosocial[Title/Abstract]

#29 behavio*[Title/Abstract] AND (therap*[Title/Abstract] OR intervention*[Title/Abstract])

#30 cognitive therap*[Title/Abstract]

#31 famil* therap*[Title/Abstract]

#32 "mutual help"[Title/Abstract]

#33 alcohol rehab*[Title/Abstract]

#34 alcohol program*[Title/Abstract]

#35 mentor*[Title/Abstract]

#36 [#22 OR #23 OR #24 OR #25 OR #26 OR #27 OR #28 OR #29 OR #30 OR #31 OR #32 OR #33 OR #34 OR #35]

*Concept 4: pharmacological interventions*

#37 pharmacology[MeSH Terms]

#38 psychopharmacology[MeSH Terms]

#39 drug therapy[MeSH Terms]

#40 alcohol deterrents[MeSH Terms]

#41 anticonvulsants[MeSH Terms]

#42 narcotic antagonists[MeSH Terms]

#43 pharmacolog*[Title/Abstract]

#44 pharmacotherap*[Title/Abstract]

#45 "opioid antagonist"[Title/Abstract] OR "opioid antagonists"[Title/Abstract]

#46 anticonvulsant*[Title/Abstract]

#47 [#37 OR #38 OR #39 OR #40 OR #41 OR #42 OR #43 OR #44 OR #45 OR #46]

#48 [#21 OR #36 OR #47]

*Concept 5: systematic review*

#49 meta-analysis as topic[MeSH Terms]

#50 meta-analysis[MeSH Terms]

#51 (meta analy*[Title/Abstract]) OR (metanaly*[Title/Abstract]) OR (metaanaly*[Title/Abstract]) OR (met analy*[Title/Abstract])

#52 (integrative research[Title/Abstract]) OR (integrative review*[Title/Abstract]) OR (integrative overview*[Title/Abstract]) OR (research integration*[Title/Abstract]) OR (research overview*[Title/Abstract]) OR (collaborative review*[Title/Abstract]) OR (collaborative overview*[Title/Abstract])

#53 (systematic review*[Title/Abstract]) OR (systematic overview*[Title/Abstract])

#54 (comparative efficacy[Title/Abstract]) OR (comparative effectiveness[Title/Abstract])

#55 (methodological overview*[Title/Abstract]) OR (methodologic* review*[Title/Abstract]) OR (quantitative review*[Title/Abstract]) OR (quantitative overview*[Title/Abstract]) OR (quantitative synthes*[Title/Abstract]) OR (pooled analy*[Title/Abstract])

#56 Embase*[Title/Abstract] OR Cinahl*[Title/Abstract] OR Cochrane[Title/Abstract] OR Medline[Title/Abstract] OR Pubmed[Title/Abstract]

#57 meta-regression[Title/Abstract] OR metaregression[Title/Abstract]

#58 meta-analysis[Publication Type]

#59 systematic[sb]

#60 (data synthes*[Title/Abstract]) OR (data extraction[Title/Abstract]) OR (data abstraction[Title/Abstract])

#61 [#49 OR #50 OR #51 OR #52 OR #53 OR #54 OR #55 OR #56 OR #57 OR #58 OR #59 OR #60]

*Combining concepts*

#62 [#8 AND #48 AND #61]

**Cochrane Database of Systematic Reviews**

*Concept 1: harmful alcohol use*

#1 alcohol-related disorders[MeSH Terms] explode all trees

#2 drinking behavior[MeSH Terms] explode all trees

#3 ("alcohol use"):ti,ab,kw

#4 (alcoholic*):ti,ab,kw

#5 (alcoholism):ti,ab,kw

#6 (alcohol AND (drink* OR intoxicat* OR abus* OR misus* OR addict* OR depend* OR disorder* OR risk* OR consum* OR withdraw* OR detox* OR treat* OR therap* OR excess* OR reduc* OR cessation OR intervention* OR abstain OR abstinence OR sober OR problem*)):ti,ab,kw

#7 (drink* AND (excess* OR heavy OR heavily OR hazard* OR binge OR harm OR harmful OR problem*)):ti,ab,kw

#8 [#1 OR #2 OR #3 OR #4 OR #5 OR #6 OR #7]

*Concept 2: screening, brief intervention, referral*

#9 mass screening[MeSH Terms] explode all trees

#10 diagnostic screening programs[MeSH Terms] explode all trees

#11 counseling[MeSH Terms] explode all trees

#12 interview, psychological[MeSH Terms] explode all trees

#13 referral and consultation[MeSH Terms] explode all trees

#14 screening:ti,ab,kw

#15 advice:ti,ab,kw

#16 referral:ti,ab,kw

#17 (brief AND (intervention* OR therap* OR interview*)):ti,ab,kw

#18 (minimal AND (intervention* OR therap* OR interview*)):ti,ab,kw

#19 (early AND (intervention* OR therap* OR interview*)):ti,ab,kw

#20 (motivat* AND (intervention* OR therap* OR interview*)):ti,ab,kw

#21 [#9 OR #10 OR #11 OR #12 OR #13 OR #14 OR #15 OR #16 OR #17 OR #18 OR #19 OR #20]

*Concept 3: psychosocial interventions*

#22 psychotherapy[MeSH Terms] explode all trees

#23 motivation[MeSH Terms] explode all trees

#24 self-help groups[MeSH Terms] explode all trees

#25 counsel*:ti,ab,kw

#26 "contingency management":ti,ab,kw

#27 "community reinforcement":ti,ab,kw

#28 (psychotherap* OR psychosocial):ti,ab,kw

#29 (behavio* AND (therap* OR intervention*)):ti,ab,kw

#30 (cognitive therap*):ti,ab,kw

#31 (famil* therap*):ti,ab,kw

#32 "mutual help":ti,ab,kw

#33 (alcohol rehab*):ti,ab,kw

#34 (alcohol program*):ti,ab,kw

#35 mentor*:ti,ab,kw

#36 [#22 OR #23 OR #24 OR #25 OR #26 OR #27 OR #28 OR #29 OR #30 OR #31 OR #32 OR #33 OR #34 OR #35]

*Concept 4: pharmacological interventions*

#37 pharmacology[MeSH Terms] explode all trees

#38 psychopharmacology[MeSH Terms] explode all trees

#39 drug therapy[MeSH Terms] explode all trees

#40 alcohol deterrents[MeSH Terms] explode all trees

#41 anticonvulsants[MeSH Terms] explode all trees

#42 narcotic antagonists[MeSH Terms] explode all trees

#43 pharmacolog*:ti,ab,kw

#44 pharmacotherap*:ti,ab,kw

#45 ("opioid antagonist" OR "opioid antagonists"):ti,ab,kw

#46 anticonvulsant*:ti,ab,kw

#47 [#37 OR #38 OR #39 OR #40 OR #41 OR #42 OR #43 OR #44 OR #45 OR #46]

*Combining concepts & Concept 5: systematic review*

#48 [#21 OR #36 OR #47]

#49 [#8 AND #48]

#50 [systematic review filter applied to #49]

**Embase**

*Concept 1: harmful alcohol use*

1. ‘alcoholism’/exp

2. ‘alcohol abuse’/exp

3. ‘drinking behavior’/exp

4. ‘alcohol withdrawal’/exp

5. (alcohol NEAR/3 (drink$ or intoxicat$ or abus$ or misus$ or addict$ or depend$ or disorder$ or risk$ or consum$ or withdraw$ or detox$ or treat$ or therap$ or excess$ or reduc$ or cessation or intervention$ or abstain or abstinence or sober)):ti,ab,kw

6. (drink$ NEAR/3 (excess$ or heavy or heavily or hazard$ or binge or harm or harmful or problem$)):ti,ab,kw

7. (alcohol NEAR/1 use):ti,ab,kw

8. (alcoholic$ or alcoholism):ti,ab,kw

9. or/1-8

*Concept 2: screening, brief intervention, referral*

10. 'screening’/exp

11. ‘counseling’/exp

12. 'psychologic test'/exp

13. ‘referral and consultation’/exp

14. (screening or advice or referral):ti,ab,kw

15. ((brief or minimal or early or motivat$) NEAR/3 (intervention$ OR therap$ OR interview$)):ti,ab,kw

16. or/10-15

*Concept 3: psychosocial interventions*

17. ‘psychosocial’

18. ‘psychotherapy’/exp

19. 'behavior therapy'

20. 'cognitive therapy'

21. motivation

22. 'self help'

23. (counseling or counselling):ti,ab,kw

24. (contingency NEAR/1 management):ti,ab,kw

25. (community NEAR/1 reinforcement):ti,ab,kw

26. (psychotherap$ or psychosocial):ti,ab,kw

27. (behavio$ NEAR/3 (therap$ or intervention$)):ti,ab,kw

28. (cognitive NEAR/1 therap$):ti,ab,kw

29. (famil$ NEAR/1 therap$):ti,ab,kw

30. (mutual NEAR/1 help):ti,ab,kw

31. (alcohol NEAR/3 rehab$):ti,ab,kw

32. (alcohol NEAR/3 program$):ti,ab,kw

33. (mentor$):ti,ab,kw

34. or/17-33

*Concept 4: pharmacological interventions*

35. ‘alcoholism therapy’

36. ‘anticonvulsive agent’/exp

37. ‘narcotic antagonists’/exp

38. psychopharmacology:ti,ab,kw

39. (pharmacolog$):ti,ab,kw

40. (pharmacotherap$):ti,ab,kw

41. (opioid NEAR/2 antagonist):ti,ab,kw

42. (anticonvulsant$):ti,ab,kw

43. or/35-42

44. 16 or 34 or 43

*Concept 5: systematic review*

45. ('meta-analysis' OR 'systematic review' OR 'meta-analysis as topic' OR 'meta analysis (topic)' OR 'systematic review (topic)') AND ('technology assessment' OR 'biomedical')

46. 'meta analysis'

47. ((systematic* NEAR/3 (review* OR overview*)):ti,ab,kw) OR ((methodologic* NEAR/3 (review* OR overview*)):ti,ab,kw)

48. ((quantitative NEAR/3 (review* OR overview* OR synthes*)):ti,ab,kw) OR ((research NEAR/3 (integrati* OR overview*)):ti,ab,kw)

49. ((integrative NEAR/3 (review* OR overview*)):ti,ab,kw) OR ((collaborative NEAR/3 (review* OR overview*)):ti,ab,kw) OR ((pool* NEAR/3 analy*):ti,ab,kw)

50. 'data synthes*':ti,ab,kw OR 'data extraction*':ti,ab,kw OR 'data abstraction*':ti,ab,kw51. (met analy* or metanaly*):ti,ab,kw,kf

51. 'meta regression*':ti,ab,kw OR 'metaregression*':ti,ab,kw

52. (comparative NEAR/3 (efficacy or effectiveness)):ti,ab,kw

53. 'meta-analy*' OR 'metaanaly*' OR 'systematic review*':ti,ab,kw

54. 'medline':ti,ab OR 'cochrane library':ti,ab OR pubmed:ti,ab OR 'embase':ti,ab OR 'cinahl':ti,ab

55. or/45-54

*Combining concepts*

58. 9 and 44 and 55

**PsycINFO**

*Concept 1: harmful alcohol use*

1. exp alcohol use disorder/

2. exp alcohol drinking patterns/

3. sobriety/

4. (alcohol NEAR/3 (drink$ or intoxicat$ or abus$ or misus$ or addict$ or depend$ or disorder$ or risk$ or consum$ or withdraw$ or detox$ or treat$ or therap$ or excess$ or reduc$ or cessation or intervention$ or abstain or abstinence or sober)).tw

5. (drink$ NEAR/3 (excess$ or heavy or heavily or hazard$ or binge or harm or harmful or problem$)).tw

6. (“alcohol use” or alcoholic$ or alcoholism).tw

7. or/1-6

*Concept 2: screening, brief intervention, referral*

8. exp health screening/

9. psychodiagnosis/

10. exp brief psychotherapy/

11. exp counseling/

12. professional referral/

13. (screening or advice or referral).tw

14. ((brief or minimal or early or motivat$) NEAR/3 (intervention$ OR therap$ OR interview$)).tw

15. or/8-14

*Concept 3: psychosocial interventions*

16. support groups/

17. self-help techniques/

18. group psychotherapy/

19. cognitive behavior therapy/ or behavior therapy/

20. motivational interviewing/

21. (community NEAR/1 reinforcement).tw

22. (psychotherap$ or psychosocial).tw

23. (behavio$ NEAR/3 (therap$ or intervention$)).tw

24. (cognitive NEAR/1 therap$).tw

25. (famil$ NEAR/1 therap$).tw

26. (mutual NEAR/1 help).tw

27. (alcohol NEAR/3 rehab$).tw

28. (alcohol NEAR/3 program$).tw

29. (mentor$).tw

30. (counseling or counselling).tw

31. (contingency NEAR/1 management).tw

32. or/16-31

*Concept 4: pharmacological interventions*

33. exp alcohol treatment/

34. pharmacotherap$.tw

35. psychopharmacolog$.tw

36. (drug NEAR/3 therap$).tw

37. (alcohol NEAR/3 deterrent).tw

38. Opioid?antagonist.tw

40. Anticonvulsant$.tw

41. (narcotic?antagonist or narcotic?agonist or narcotic?drug?).tw

42. pharmacolog$.tw

43. or/33-42

44. 15 or 33 or 43

*Concept 5: systematic review*

45. meta-analysis/ or systematic review/ or meta-analysis as topic/ or "meta analysis (topic)"/ or "systematic review (topic)"/ or exp technology assessment, biomedical/

46. (meta-analysis).pt

47. ((systematic* NEAR/3 (review* or overview*)) or (methodologic* NEAR/3 (review* or overview*))).ti,ab,kw,kf

48. ((quantitative NEAR/3 (review* or overview* or synthes*)) or (research NEAR/3 (integrati* or overview*))).ti,ab,kw,kf

49. ((integrative NEAR/3 (review* or overview*)) or (collaborative NEAR/3 (review* or overview*)) or (pool* NEAR/3 analy*)).ti,ab,kw,kf

50. (data synthes* or data extraction* or data abstraction*).ti,ab,kw,kf

51. (met analy* or metanaly*).ti,ab,kw,kf

52. (meta regression* or metaregression*).ti,ab,kw,kf

53. (comparative NEAR/3 (efficacy or effectiveness)).ti,ab,kw,kf

54. (meta-analy* or metaanaly* or systematic review*).mp

55. (medline or cochrane or pubmed or embase or cinahl).ti,ab,hw

56. (meta-analysis or systematic review).md

57. or/45-56

*Combining concepts*

58. 7 and 44 and 57

**InaHTA database**

((alcohol) OR (alcohol)[mh]) AND ("systematic review" OR "meta-analysis")
