# Supplementary material for: Which interventions for alcohol use should be included in a universal healthcare benefit package? An umbrella review of targeted interventions to address harmful drinking and dependence
Source: BMC Public Health. 2023 Feb 23;23:382. doi: 10.1186/s12889-023-15152-6 (PMC9948368; doi:10.1186/s12889-023-15152-6)
Supplement: Supplementary file 5 — Additional file 5. [file 12889_2023_15152_MOESM5_ESM.docx]

**Supplement 5.** Studies excluded at full text screening.

**Exclusion reason 1:** intervention is not universal or not primarily targeting alcohol use

1. Foxcroft, D.R.; Tsertsvadze, A. Universal family-based prevention programs for alcohol misuse in young people. 2011.
2. Hodder, R.K.; Freund, M.; Wolfenden, L.; Bowman, J.; Nepal, S.; Dray, J.; Kingsland, M.; Yoong, S.L.; Wiggers, J. Systematic review of universal school-based resilience interventions targeting adolescent tobacco, alcohol or illicit substance use: A meta-analysis 2017. DOI: 10.1016/j.ypmed.2017.04.003
3. Gordon CS; Jones SC; Kervin L. Effectiveness of alcohol media literacy programmes: a systematic literature review. 2015. DOI: 10.1093/her/cyv015
4. Foxcroft, D.R.; Tsertsvadze, A. Universal multi-component prevention programs for alcohol misuse in young people. 2011.
5. Cho MK; Cho YH. Do Alcohol Prevention Programs Influence Adolescents' Drinking Behaviors? A Systematic Review and Meta-Analysis. 2021. DOI: 10.3390/ijerph18168524
6. Bo A; Hai AH; Jaccard J. Parent-based interventions on adolescent alcohol use outcomes: A systematic review and meta-analysis. 2018. DOI: 10.1016/j.drugalcdep.2018.05.031
7. Georgie J M; Sean H; Deborah M C; Matthew H; Rona C. Peer-led interventions to prevent tobacco, alcohol and/or drug use among young people aged 11-21 years: a systematic review and meta-analysis. 2016. DOI: 10.1111/add.13224
8. Tancred T; Melendez-Torres GJ; Paparini S; Fletcher A; Stansfield C; Thomas J; Campbell R; Taylor S; Bonell C. 2019. DOI: 10.3310/phr07170
9. Wang N; Sun X; Yin L; Liu H; Ruan Y; Shao Y; Qian HZ; Vermund SH. Meta-Analysis of Interventions for Reducing Number of Sexual Partners and Drug and Alcohol Abuse among People Living with HIV/AIDS. 2013. DOI: 10.4172/2155-6113.1000213
10. Sheeran P; Wright CE; Avishai A; Villegas ME; Lindemans JW; Klein WMP; Rothman AJ; Miles E; Ntoumanis N. Self-determination theory interventions for health behavior change: Meta-analysis and meta-analytic structural equation modeling of randomized controlled trials. 2020. DOI: 10.1037/ccp0000501
11. Steinka-Fry KT; Tanner-Smith EE; Grant S. Effects of 21st birthday brief interventions on college student celebratory drinking: A systematic review and meta-analysis. 2015. DOI: 10.1016/j.addbeh.2015.06.001
12. Jonas, D.E.; Amick, H.R.; Feltner, C.; Wines, R.; Shanahan, E.; Rowe, C.J.; Garbutt, J.C.. Genetic polymorphisms and response to medications for alcohol use disorders: A systematic review and meta-analysis 2014. DOI: 10.2217/pgs.14.121
13. Chiang CY; Choi KC; Ho KM; Yu SF. Effectiveness of nurse-led patient-centered care behavioral risk modification on secondary prevention of coronary heart disease: A systematic review. 2018. DOI: 10.1016/j.ijnurstu.2018.04.012
14. Donaghy, M.E.. Exercise can seriously improve your mental health: Fact of fiction? 2007. DOI: 10.1080/14038190701395838
15. Mansueto G; Martino F; Palmieri S; Scaini S; Ruggiero GM; Sassaroli S; Caselli G. Desire Thinking across addictive behaviours: A systematic review and meta-analysis. 2019. DOI: 10.1016/j.addbeh.2019.06.007
16. Foulds, J.; Newton-Howes, G.; Guy, N.H.; Boden, J.M.; Mulder, R.T.. Dimensional personality traits and alcohol treatment outcome: a systematic review and meta-analysis 2017. DOI: 10.1111/add.13810
17. Champion KE; Parmenter B; McGowan C; Spring B; Wafford QE; Gardner LA; Thornton L; McBride N; Barrett EL; Teesson M; Newton NC. Effectiveness of school-based eHealth interventions to prevent multiple lifestyle risk behaviours among adolescents: a systematic review and meta-analysis. 2019. DOI: 10.1016/S2589-7500(19)30088-3
18. Carter P; Bignardi G; Hollands GJ; Marteau TM. Information-based cues at point of choice to change selection and consumption of food, alcohol and tobacco products: a systematic review. 2018. DOI: 10.1186/s12889-018-5280-5
19. Del Re, A.C.; Finney, J.W.; Maisel, N.C.; Blodgett, J.C. Placebo group improvement in trials of pharmacotherapies for alcohol use disorders: A multivariate meta-analysis examining change over time 2012. DOI: 10.1111/j.1530-0277.2012.01803.x
20. Lyman, D. Russell; Braude, Lisa; George, Preethy; Dougherty, Richard H.; Daniels, Allen S.; Ghose, Sushmita Shoma; Delphin-Rittmon, Miriam E. Consumer and family psychoeducation: Assessing the evidence. 2014. DOI: 10.1176/appi.ps.201300266
21. Salazar de Pablo G; De Micheli A; Solmi M; Oliver D; Catalan A; Verdino V; Di Maggio L; Bonoldi I; Radua J; Baccaredda Boy O; Provenzani U; Ruzzi F; Calorio F; Nosari G; Di Marco B; Famularo I; Montealegre I; Signorini L; Molteni S; Filosi E; Mensi M; Balottin U; Politi P; Shin JI; Correll CU; Arango C; Fusar-Poli P. Universal and Selective Interventions to Prevent Poor Mental Health Outcomes in Young People: Systematic Review and Meta-analysis. 2021. DOI: 10.1097/HRP.0000000000000294
22. McKay FH; Cheng C; Wright A; Shill J; Stephens H; Uccellini M. Evaluating mobile phone applications for health behaviour change: A systematic review. 2018. DOI: 10.1177/1357633X16673538
23. Cooke R; Dahdah M; Norman P; French DP. How well does the theory of planned behaviour predict alcohol consumption? A systematic review and meta-analysis. 2016. DOI: 10.1080/17437199.2014.947547
24. Mehta S; Janzen S; Cotoi A; Rice D; Owens K; Teasell R. Screening questionnaires for substance abuse post brain injury: a review. 2019. DOI: 10.1080/02699052.2019.1567938
25. Hershberger, A.; Um, M.; Cyders, M. The role of the UPPS-p impulsive personality traits in cognitive behavioral therapy based substance use treatment: A meta-analysis 2017. DOI: 10.1111/acer.13391
26. Sancho M; De Gracia M; Rodríguez RC; Mallorquí-Bagué N; Sánchez -González J; Trujols J; Sánchez I; Jimenez-Murcia S; Menchón JM. Mindfulness-Based Interventions for the Treatment of Substance and Behavioral Addictions: A Systematic Review. 2018. DOI: 10.3389/fpsyt.2018.00095
27. Rosansky JA; Rosenberg H. A systematic review of reasons for abstinence from alcohol reported by lifelong abstainers, current abstainers and former problem-drinkers. 2020. DOI: 10.1111/dar.13119
28. Rundall TG; Bruvold WH. A meta-analysis of school-based smoking and alcohol use prevention programs. 1988. DOI: 10.1177/109019818801500306
29. Bruvold WH. A meta-analysis of the California school-based risk reduction program. 1990. DOI: 10.2190/7CRH-5R8T-MHR6-6UD7
30. Vancampfort D; De Hert M; Stubbs B; Soundy A; De Herdt A; Detraux J; Probst M. A systematic review of physical activity correlates in alcohol use disorders. 2015. DOI: 10.1016/j.apnu.2014.08.006
31. Calabria B; Shakeshaft AP; Havard A. A systematic and methodological review of interventions for young people experiencing alcohol-related harm. 2011. DOI: 10.1111/j.1360-0443.2011.03418.x
32. Skara S; Sussman S. A review of 25 long-term adolescent tobacco and other drug use prevention program evaluations. 2003. DOI: 10.1016/s0091-7435(03)00166-x
33. Iovieno N; Tedeschini E; Bentley KH; Evins AE; Papakostas GI. Antidepressants for major depressive disorder and dysthymic disorder in patients with comorbid alcohol use disorders: a meta-analysis of placebo-controlled randomized trials. 2011. DOI: 10.4088/JCP.10m06217
34. Liu J; Wang LN. Baclofen for alcohol withdrawal. 2015. DOI: 10.1002/14651858.CD008502.pub4
35. Lai JY; Kalk N; Roberts E. The effectiveness and tolerability of anti-seizure medication in alcohol withdrawal syndrome: a systematic review, meta-analysis and GRADE of the evidence. 2021. DOI: 10.1111/add.15510
36. Sarai M; Tejani AM; Chan AH; Kuo IF; Li J. Magnesium for alcohol withdrawal. 2013. DOI: 10.1002/14651858.CD008358.pub2
37. Naish KR; Vedelago L; MacKillop J; Amlung M. Effects of neuromodulation on cognitive performance in individuals exhibiting addictive behaviors: A systematic review. 2018. DOI: 10.1016/j.drugalcdep.2018.08.018
38. Berks J; McCormick R. Screening for alcohol misuse in elderly primary care patients: a systematic literature review. 2008. DOI: 10.1017/S1041610208007497
39. Wisdom, J.P.; Manuel, J.I.; Drake, R.E. Substance use disorder among people with first-episode psychosis: A systematic review of course and treatment. 2011. DOI: 10.1176/appi.ps.62.9.1007
40. Stockings E; Bartlem K; Hall A; Hodder R; Gilligan C; Wiggers J; Sherker S; Wolfenden L. Whole-of-community interventions to reduce population-level harms arising from alcohol and other drug use: a systematic review and meta-analysis. 2018. DOI: 10.1111/add.14277
41. Johnson, I.N.S.; Shovestul, B.J.; Niciu, M.J.; Li, F.; Bloch, M.H. 1.60 ANTIDEPRESSANT TREATMENT OF MDD IN PATIENTS WITH COMORBID ALCOHOL USE DISORDER: A META-ANALYSIS OF RANDOMIZED PLACEBO-CONTROLLED TRIALS WITH DISCUSSION OF PEDIATRIC IMPLICATIONS. 2019. DOI: 10.1016/j.jaac.2019.08.082
42. van Ginneken N; Chin WY; Lim YC; Ussif A; Singh R; Shahmalak U; Purgato M; Rojas-GarcÃ­a A; Uphoff E; McMullen S; Foss HS; Thapa Pachya A; Rashidian L; Borghesani A; Henschke N; Chong LY; Lewin S. Primary-level worker interventions for the care of people living with mental disorders and distress in low- and middle-income countries. 2021. DOI: 10.1002/14651858.CD009149.pub3
43. Pullar J; Allen L; Townsend N; Williams J; Foster C; Roberts N; Rayner M; Mikkelsen B; Branca F; Wickramasinghe K. The impact of poverty reduction and development interventions on non-communicable diseases and their behavioural risk factors in low and lower-middle income countries: A systematic review. 2018. DOI: 10.1371/journal.pone.0193378
44. Teesson M; Newton NC; Barrett EL. Australian school-based prevention programs for alcohol and other drugs: a systematic review. 2012. DOI: 10.1111/j.1465-3362.2012.00420.x
45. Amato L; Minozzi S; Davoli M. Efficacy and safety of pharmacological interventions for the treatment of the Alcohol Withdrawal Syndrome. 2011. DOI: 10.1002/14651858.CD008537.pub2
46. Goss CW; Van Bramer LD; Gliner JA; Porter TR; Roberts IG; Diguiseppi C. Increased police patrols for preventing alcohol-impaired driving. 2008. DOI: 10.1002/14651858.CD005242.pub2
47. Vodopivecâ Jamsek, V; de Jongh, T; Gurolâ Urganci, I; Atun, R; Car, J. Mobile phone messaging for preventive health care 2012. DOI: 10.1002/14651858.CD007457.pub2

**Exclusion reason 2:** outcomes not related to alcohol use or alcohol-attributable mortality, morbidity, or accident and injury

1. Boffo M; Zerhouni O; Gronau QF; van Beek RJJ; Nikolaou K; Marsman M; Wiers RW. Cognitive Bias Modification for Behavior Change in Alcohol and Smoking Addiction: Bayesian Meta-Analysis of Individual Participant Data. 2019. DOI: 10.1007/s11065-018-9386-4
2. Mason M; Ola B; Zaharakis N; Zhang J. Text messaging interventions for adolescent and young adult substance use: a meta-analysis. 2015. DOI: 10.1007/s11121-014-0498-7
3. Getty CA; Morande A; Lynskey M; Weaver T; Metrebian N. Mobile telephone-delivered contingency management interventions promoting behaviour change in individuals with substance use disorders: a meta-analysis. 2019. DOI: 10.1111/add.14725
4. Weisel KK; Fuhrmann LM; Berking M; Baumeister H; Cuijpers P; Ebert DD. Standalone smartphone apps for mental health-a systematic review and meta-analysis. 2019. DOI: 10.1038/s41746-019-0188-8
5. Hammond DA; Rowe JM; Wong A; Wiley TL; Lee KC; Kane-Gill SL. Patient Outcomes Associated With Phenobarbital Use With or Without Benzodiazepines for Alcohol Withdrawal Syndrome: A Systematic Review. 2017. DOI: 10.1177/0018578717720310
6. Simioni N; Rolland B; Cottencin O. Interventions for Increasing Alcohol Treatment Utilization Among Patients with Alcohol Use Disorders from Emergency Departments: A Systematic Review. 2015. DOI: 10.1016/j.jsat.2015.06.003
7. Ulrichsen, J.; Ntais, C.; Pakos, E.; Kyzas, P.; Ioannidis, J.P.A. Benzodiazepines for alcohol withdrawal 2006. DOI: 10.1002/14651858.CD005063.pub2
8. Budworth L; Prestwich A; Lawton R; Kotzé A; Kellar I. Preoperative Interventions for Alcohol and Other Recreational Substance Use: A Systematic Review and Meta-Analysis. 2019. DOI: 10.3389/fpsyg.2019.00034
9. Amato L; Minozzi S; Vecchi S; Davoli M. Benzodiazepines for alcohol withdrawal. 2010. DOI: 10.1002/14651858.CD005063.pub3
10. Glass JE; Hamilton AM; Powell BJ; Perron BE; Brown RT; Ilgen MA. Specialty substance use disorder services following brief alcohol intervention: a meta-analysis of randomized controlled trials. 2015. DOI: 10.1111/add.12950
11. Connolly SM; Vanchu-Orosco M; Warner J; Seidi PA; Edwards J; Boath E; Irgens AC. Mental health interventions by lay counsellors: a systematic review and meta-analysis. 2021. DOI: 10.2471/BLT.20.269050
12. Roberts NP; Roberts PA; Jones N; Bisson JI. Psychological therapies for post-traumatic stress disorder and comorbid substance use disorder. 2016. DOI: 10.1002/14651858.CD010204.pub2
13. Nunes EV; Levin FR. Treatment of depression in patients with alcohol or other drug dependence: a meta-analysis. 2004. DOI: 10.1001/jama.291.15.1887
14. Rojo-Mira J; Pineda-Álvarez M; Zapata-Ospina JP. Efficacy and Safety of Anticonvulsants for the Inpatient Treatment of Alcohol Withdrawal Syndrome: A Systematic Review and Meta-analysis. 2021. DOI: 10.1093/alcalc/agab057
15. Zhang JJQ; Fong KNK; Ouyang RG; Siu AMH; Kranz GS. Effects of repetitive transcranial magnetic stimulation (rTMS) on craving and substance consumption in patients with substance dependence: a systematic review and meta-analysis. 2019. DOI: 10.1111/add.14753
16. Ray LA; Meredith LR; Kiluk BD; Walthers J; Carroll KM; Magill M. Combined Pharmacotherapy and Cognitive Behavioral Therapy for Adults With Alcohol or Substance Use Disorders: A Systematic Review and Meta-analysis. 2020. DOI: 10.1001/jamanetworkopen.2020.8279
17. Cristea IA; Kok RN; Cuijpers P. The Effectiveness of Cognitive Bias Modification Interventions for Substance Addictions: A Meta-Analysis. 2016. DOI: 10.1371/journal.pone.0162226
18. Mostafavi SA; Khaleghi A; Mohammadi MR. Noninvasive brain stimulation in alcohol craving: A systematic review and meta-analysis. 2020. DOI: 10.1016/j.pnpbp.2020.109938
19. Cao HJ; Cheng N; Wang RT; Huang XY; Wu JR. Comparison between Xingnaojing Injection () and Naloxone in Treatment of Acute Alcohol Intoxication: An Updated Systematic Review and Meta-Analysis of Randomized Controlled Trials. 2019. DOI: 10.1007/s11655-019-3037-3
20. Mehta K; Hoadley A; Ray LA; Kiluk BD; Carroll KM; Magill M. Cognitive-Behavioral Interventions Targeting Alcohol or Other Drug Use and Co-Occurring Mental Health Disorders: A Meta-Analysis. 2021. DOI: 10.1093/alcalc/agab016
21. Ahmed S; Stanciu CN; Kotapati PV; Ahmed R; Bhivandkar S; Khan AM; Afridi A; Qureshi M; Esang M. Effectiveness of Gabapentin in Reducing Cravings and Withdrawal in Alcohol Use Disorder: A Meta-Analytic Review. 2019. DOI: 10.4088/PCC.19r02465
22. Minozzi S; Amato L; Vecchi S; Davoli M. Anticonvulsants for alcohol withdrawal. 2010. DOI: 10.1002/14651858.CD005064.pub3
23. Liu J; Wang LN. Baclofen for alcohol withdrawal. 2019. DOI: 10.1002/14651858.CD008502.pub6
24. Holbrook AM; Crowther R; Lotter A; Cheng C; King D. Meta-analysis of benzodiazepine use in the treatment of acute alcohol withdrawal. 1999.
25. Wobrock, T.; Soyka, M.. Pharmacotherapy of patients with schizophrenia and substance abuse 2009. DOI: 10.1517/14656560802694655
26. Davey CJ; Landy MS; Pecora A; Quintero D; McShane KE. A realist review of brief interventions for alcohol misuse delivered in emergency departments. 2015. DOI: 10.1186/s13643-015-0024-4
27. Mantzari E; Vogt F; Shemilt I; Wei Y; Higgins JP; Marteau TM. Personal financial incentives for changing habitual health-related behaviors: A systematic review and meta-analysis. 2015. DOI: 10.1016/j.ypmed.2015.03.001
28. Geuijen, P.M.; van den Broek, S.J.M.; Dijkstra, B.A.G.; Kuppens, J.M.; de Haan, H.A.; de Jong, C.A.J.; Schene, A.H.; Atsma, F.; Schellekens, A.F.A. Success rates of monitoring for healthcare professionals with a substance use disorder: A meta-analysis 2021. DOI: 10.3390/jcm10020264
29. Hayes L; McParlin C; Azevedo LB; Jones D; Newham J; Olajide J; McCleman L; Heslehurst N. The Effectiveness of Smoking Cessation, Alcohol Reduction, Diet and Physical Activity Interventions in Improving Maternal and Infant Health Outcomes: A Systematic Review of Meta-Analyses. 2021. DOI: 10.3390/nu13031036
30. Walsh, S.; Haroon, S.; Nirantharakumar, K.; Bhala, N. Approaches to alcohol screening in secondary care: A review and meta-analysis 2017. DOI:
31. De Leon, Elaine; Fuentes, Laura W.; Cohen, Joanna E. Characterizing periodic messaging interventions across health behaviors and media: Systematic review. 2014. DOI: 10.2196/jmir.2837
32. Kuitunen-Paul S; Roerecke M. Alcohol Use Disorders Identification Test (AUDIT) and mortality risk: a systematic review and meta-analysis. 2018. DOI: 10.1136/jech-2017-210078
33. Brooks, J.; Lawlor, S.; Turetzkin, S.; Goodnight, C.W.; Galantino, M.L. Yoga for Substance Use Disorder in Women: A Systematic Review 2020. DOI: 10.17761/2021-D-20-00008
34. Salloum, I.M.; Jones, Y.O. Efficacy of pharmacotherapy for comorbid major depression and substance use disorders: A review 2008. DOI: 10.2174/157340008783743785
35. Barrons R; Roberts N. The role of carbamazepine and oxcarbazepine in alcohol withdrawal syndrome. 2010. DOI: 10.1111/j.1365-2710.2009.01098.x
36. Mueller, G.; Schumacher, P.; Wetzlmair, J.; Pallauf, M.. Screening questionnaires to identify problem drinking in the primary care setting: a systematic review 2016. DOI: 10.1007/s10389-015-0694-3
37. Johann, A.; Baglioni, C.; Hertenstein, E.; Riemann, D.; Spiegelhalder, K. Prevention of mental disorders through cognitive behavioral therapy for insomnia 2015. DOI: 10.1007/s11818-015-0008-6
38. Mdege ND; Watson J. Predictors of study setting (primary care vs. hospital setting) among studies of the effectiveness of brief interventions among heavy alcohol users: a systematic review. 2013. DOI: 10.1111/dar.12036
39. Von Sydow, K.; Schindler, A.; Beher, S.; Schweitzer-Rothers, J.; Retzlaff, R. The efficacy of systemic therapy for substance use disorders in adult and adolescent index patients 2010. DOI: 10.1024/0939-5911/a000009
40. Taggart J; Williams A; Dennis S; Newall A; Shortus T; Zwar N; Denney-Wilson E; Harris MF. A systematic review of interventions in primary care to improve health literacy for chronic disease behavioral risk factors. 2012. DOI: 10.1186/1471-2296-13-49
41. Littlejohn C. Does socio-economic status influence the acceptability of, attendance for, and outcome of, screening and brief interventions for alcohol misuse: a review. 2006. DOI: 10.1093/alcalc/agl053
42. Ariss T; Fairbairn CE. The effect of significant other involvement in treatment for substance use disorders: A meta-analysis. 2020. DOI: 10.1037/ccp0000495
43. Ranasinghe, I.; Norman, I.; Lau-Walker, M. Variables associated with alcohol relapse and psychosocial interventions to prevent alcohol relapse in liver transplant patients for alcoholic liver disease: A systematic review 2015.
44. Rayburn WF; Bogenschutz MP. Pharmacotherapy for pregnant women with addictions. 2004. DOI: 10.1016/j.ajog.2004.06.082
45. Ahmed, S.; Stanciu, C.N.; Kotapati, P.V.; Ahmed, R.; Bhivandkar, S.; Khan, A.M.; Afridi, A.; Qureshi, M.; Esang, M.. A meta-analytic review 2019. DOI: 10.4088/PCC.19r02465
46. Singh T; Roberts K; Cohen T; Cobb N; Wang J; Fujimoto K; Myneni S. Social Media as a Research Tool (SMaaRT) for Risky Behavior Analytics: Methodological Review. 2020. DOI: 10.2196/21660
47. Carrico AW; Zepf R; Meanley S; Batchelder A; Stall R. Critical Review: When the Party is Over: A Systematic Review of Behavioral Interventions for Substance-Using Men Who Have Sex with Men. 2016. DOI: 10.1097/QAI.0000000000001102
48. O'Connell H; Chin AV; Hamilton F; Cunningham C; Walsh JB; Coakley D; Lawlor BA. A systematic review of the utility of self-report alcohol screening instruments in the elderly. 2004. DOI: 10.1002/gps.1214
49. Foulds JA; Adamson SJ; Boden JM; Williman JA; Mulder RT. Depression in patients with alcohol use disorders: Systematic review and meta-analysis of outcomes for independent and substance-induced disorders. 2015. DOI: 10.1016/j.jad.2015.06.024
50. Kamioka H; Okada S; Tsutani K; Park H; Okuizumi H; Handa S; Oshio T; Park SJ; Kitayuguchi J; Abe T; Honda T; Mutoh Y. Effectiveness of animal-assisted therapy: A systematic review of randomized controlled trials. 2014. DOI: 10.1016/j.ctim.2013.12.016
51. Graves DL; Carson DG; Poole N; Patel DT; Bigalky J; Green CR; Cook JL. Directive clinique n° 405 : Dépistage et conseils en matière de consommation d'alcool pendant la grossesse. 2020. DOI: 10.1016/j.jogc.2020.07.005
52. Bauer IE; Soares JC; Nielsen DA. The role of opioidergic genes in the treatment outcome of drug addiction pharmacotherapy: A systematic review. 2015. DOI: 10.1111/ajad.12172
53. De Groot F; Morrens M; Dom G. [Acceptance and commitment therapy (ACT) and addiction: a literature review]. 2014.
54. Ditter SM; Elder RW; Shults RA; Sleet DA; Compton R; Nichols JL. Effectiveness of designated driver programs for reducing alcohol-impaired driving: a systematic review. 2005. DOI: 10.1016/j.amepre.2005.02.013
55. Hawn SE; Cusack SE; Amstadter AB. A Systematic Review of the Self-Medication Hypothesis in the Context of Posttraumatic Stress Disorder and Comorbid Problematic Alcohol Use. 2020. DOI: 10.1002/jts.22521
56. GonÃ§alves JP; Lucchetti G; Menezes PR; Vallada H. Religious and spiritual interventions in mental health care: a systematic review and meta-analysis of randomized controlled clinical trials. 2015. DOI: 10.1017/S0033291715001166
57. Shorter GW; Bray JW; Giles EL; O'Donnell AJ; Berman AH; Holloway A; Heather N; Barbosa C; Stockdale KJ; Scott SJ; Clarke M; Newbury-Birch D. The Variability of Outcomes Used in Efficacy and Effectiveness Trials of Alcohol Brief Interventions: A Systematic Review. 2019.
58. Krebs P; Norcross JC; Nicholson JM; Prochaska JO. Stages of change and psychotherapy outcomes: A review and meta-analysis. 2018. DOI: 10.1002/jclp.22683
59. Berner MM; Kriston L; Bentele M; Härter M. The alcohol use disorders identification test for detecting at-risk drinking: a systematic review and meta-analysis. 2007. DOI: 10.15288/jsad.2007.68.461
60. Bray JW; Cowell AJ; Hinde JM. A systematic review and meta-analysis of health care utilization outcomes in alcohol screening and brief intervention trials. 2011. DOI: 10.1097/MLR.0b013e318203624f
61. Smedslund G; Berg RC; Hammerström KT; Steiro A; Leiknes KA; Dahl HM; Karlsen K. Motivational interviewing for substance abuse. 2011. DOI: 10.1002/14651858.CD008063.pub2
62. Livingston N; Ameral V; Hocking E; Leviyah X; Timko C. Interventions to Improve Post-Detoxification Treatment Engagement and Alcohol Recovery: Systematic Review of Intervention Types and Effectiveness. 2021. DOI: 10.1093/alcalc/agab021
63. Magill M; Ray L; Kiluk B; Hoadley A; Bernstein M; Tonigan JS; Carroll K. A meta-analysis of cognitive-behavioral therapy for alcohol or other drug use disorders: Treatment efficacy by contrast condition. 2019. DOI: 10.1037/ccp0000447
64. Miller, W.R.; Wilbourne, P.L.. Mesa Grande: A methodological analysis of clinical trials of treatments for alcohol use disorders 2002. DOI: 10.1046/j.1360-0443.2002.00019.x
65. Steinkamp, J.; Goldblatt, N.; La Vertu, A.; Borodovsky, J.; Marsch, L.; Schuman-Olivier, Z.. Technology interventions for medication adherence in addiction psychiatry: Systematic scoping review 2019. DOI: 10.1002/ajad.12887
66. Minnich, Amelia; Erford, Bradley T.; Bardhoshi, Gerta; Atalay, ZÃ¼mra. Systematic review of the Michigan Alcoholism Screening Test. 2018. DOI: 10.1002/jcad.12207
67. Mays KL; Clark DL; Gordon AJ. Treating addiction with tunes: a systematic review of music therapy for the treatment of patients with addictions. 2008. DOI: 10.1080/08897070802418485
68. Biernacka, J.; Coombes, B.; Frank, J.; Hodgkinson, C.; Batzler, A.; Colby, C.; Geske, J.; Gueorguieva, R.; Goldman, D.; Rietschel, M.; O'Malley, S.; Mann, K.F.; Anton, R.; Karpyak, V. GENOME-WIDE ANALYSIS OF PHARMACOGENOMIC EFFECTS ON ALCOHOL USE DISORDER TREATMENT OUTCOMES 2019. DOI: 10.1016/j.euroneuro.2019.08.173
69. Witt K; Chitty KM; Wardhani R; Värnik A; de Leo D; Kõlves K. Effect of alcohol interventions on suicidal ideation and behaviour: A systematic review and meta-analysis. 2021. DOI: 10.1016/j.drugalcdep.2021.108885
70. Toner P; Behnke JR; Andersen P; McCambridge J. Alcohol screening and assessment measures for young people: A systematic review and meta-analysis of validation studies. 2019. DOI: 10.1016/j.drugalcdep.2019.01.030
71. Chamorro AJ; Marcos M; MirÃ³n-Canelo JA; Pastor I; González-Sarmiento R; Laso FJ. Association of Âµ-opioid receptor (OPRM1) gene polymorphism with response to naltrexone in alcohol dependence: a systematic review and meta-analysis. 2012. DOI: 10.1111/j.1369-1600.2012.00442.x
72. Martínez-Vispo C; Martínez Ú; López-Durán A; Fernández Del Río E; Becoña E. Effects of behavioural activation on substance use and depression: a systematic review. 2018. DOI: 10.1186/s13011-018-0173-2
73. Lange S; Shield K; Monteiro M; Rehm J. Facilitating Screening and Brief Interventions in Primary Care: A Systematic Review and Meta-Analysis of the AUDIT as an Indicator of Alcohol Use Disorders. 2019. DOI: 10.1111/acer.14171
74. Williams SB; Whitlock EP; Edgerton EA; Smith PR; Beil TL. Counseling about proper use of motor vehicle occupant restraints and avoidance of alcohol use while driving: a systematic evidence review for the U.S. Preventive Services Task Force. 2007. DOI: 10.7326/0003-4819-147-3-200708070-00009
75. Nakimuli-Mpungu E; Musisi S; Smith CM; Von Isenburg M; Akimana B; Shakarishvili A; Nachega JB; Mills EJ; Chibanda D; Ribeiro M; V Williams A; Joska JA. Mental health interventions for persons living with HIV in low- and middle-income countries: a systematic review. 2021. DOI: 10.1002/jia2.25722
76. Scott S; Beyer F; Parkinson K; Muir C; Graye A; Kaner E; Stead M; Power C; Fitzgerald N; Bradley J; Wrieden W; Adamson A. Non-Pharmacological Interventions to Reduce Unhealthy Eating and Risky Drinking in Young Adults Aged 18-25 Years: A Systematic Review and Meta-Analysis. 2018. DOI: 10.3390/nu10101538
77. Pidd K; Roche AM. How effective is drug testing as a workplace safety strategy? A systematic review of the evidence. 2014. DOI: 10.1016/j.aap.2014.05.012
78. Mahoney JJ 3rd; Hanlon CA; Marshalek PJ; Rezai AR; Krinke L. Transcranial magnetic stimulation, deep brain stimulation, and other forms of neuromodulation for substance use disorders: Review of modalities and implications for treatment. 2020. DOI: 10.1016/j.jns.2020.117149
79. Yu CH; Guarna G; Tsao P; Jesuthasan JR; Lau AN; Siddiqi FS; Gilmour JA; Ladha D; Halapy H; Advani A. Incentivizing health care behaviors in emerging adults: a systematic review. 2016. DOI: 10.2147/PPA.S102574
80. Bradley KA; Boyd-Wickizer J; Powell SH; Burman ML. Alcohol screening questionnaires in women: a critical review. 1998. DOI: 10.1001/jama.280.2.166
81. Scott-Sheldon LAJ; DeMartini KS; Carey KB; Carey MP. Alcohol Interventions for College Students Improves Antecedents of Behavioral Change: Results from a Meta-Analysis of 34 Randomized Controlled Trials. 2009. DOI: 10.1521/jscp.2009.28.7.799
82. Trapero-Bertran M; Gil-Doménech D; Vargas-Martínez AM. Economic evaluations of interventions aimed at the prevention, treatment and/or rehabilitation of alcohol-related disorders: a systematic review. 2021. DOI: 10.20882/adicciones.1649
83. Sheridan Rains L; Steare T; Mason O; Johnson S. Improving substance misuse outcomes in contingency management treatment with adjunctive formal psychotherapy: a systematic review and meta-analysis. 2020. DOI: 10.1136/bmjopen-2019-034735
84. Khusid, Marina A.; Vythilingam, Meena. The emerging role of mindfulness meditation as effective self-management strategy, Part 2: Clinical implications for chronic pain, substance misuse, and insomnia. 2016. DOI: 10.7205/MILMED-D-14-00678
85. Awissi DK; Lebrun G; Coursin DB; Riker RR; Skrobik Y. Alcohol withdrawal and delirium tremens in the critically ill: a systematic review and commentary. 2013. DOI: 10.1007/s00134-012-2758-y
86. Magill M; Gaume J; Apodaca TR; Walthers J; Mastroleo NR; Borsari B; Longabaugh R. The technical hypothesis of motivational interviewing: a meta-analysis of MI's key causal model. 2014. DOI: 10.1037/a0036833
87. Vanderplasschen, W.; Wolf, J.; Rapp, R.C.; Broekaert, E. Effectiveness of different models of case management for substance-abusing populations 2007. DOI: 10.1080/02791072.2007.10399867
88. Bully P; Sánchez Á; Zabaleta-del-Olmo E; Pombo H; Grandes G. Evidence from interventions based on theoretical models for lifestyle modification (physical activity, diet, alcohol and tobacco use) in primary care settings: A systematic review. 2015. DOI: 10.1016/j.ypmed.2014.12.020
89. Chiesa, A.; Serretti, A. A systematic review of neurobiological and clinical features of mindfulness meditations 2009. DOI: 10.1017/S0033291709991747
90. Lie HC; Juvet LK; Street RL Jr; Gulbrandsen P; Mellblom AV; Brembo EA; Eide H; Heyn L; Saltveit KH; Strömme H; Sundling V; Turk E; Menichetti J. Effects of Physicians' Information Giving on Patient Outcomes: a Systematic Review. 2021. DOI: 10.1007/s11606-021-07044-5
91. Skeen S; Laurenzi CA; Gordon SL; du Toit S; Tomlinson M; Dua T; Fleischmann A; Kohl K; Ross D; Servili C; Brand AS; Dowdall N; Lund C; van der Westhuizen C; Carvajal-Aguirre L; Eriksson de Carvalho C; Melendez-Torres GJ. Adolescent Mental Health Program Components and Behavior Risk Reduction: A Meta-analysis. 2019. DOI: 10.1542/peds.2018-3488
92. Roberts, N.P.; Roberts, P.A.; Jones, N.; Bisson, J.I. Psychological therapies for post-traumatic stress disorder and comorbid substance use disorder 2016. DOI: 10.1002/14651858.CD010204.pub2
93. Flannigan K; Coons-Harding KD; Anderson T; Wolfson L; Campbell A; Mela M; Pei J. A Systematic Review of Interventions to Improve Mental Health and Substance Use Outcomes for Individuals with Prenatal Alcohol Exposure and Fetal Alcohol Spectrum Disorder. 2020. DOI: 10.1111/acer.14490
94. Willis, C; Lybrand, S; Bellamy, N. Alcohol ignition interlock programmes for reducing drink driving recidivism 2004. DOI: 10.1002/14651858.CD004168.pub2
95. Gillman MA; Lichtigfeld FJ; Young TN. Psychotropic analgesic nitrous oxide for alcoholic withdrawal states. 2007. DOI: 10.1002/14651858.CD005190.pub2

**Exclusion reason 3:** Not a systematic review of RCTs

1. Kiluk BD; Ray LA; Walthers J; Bernstein M; Tonigan JS; Magill M. Technology-Delivered Cognitive-Behavioral Interventions for Alcohol Use: A Meta-Analysis. 2019. DOI: 10.1111/acer.14189
2. Bastola, Mrigendra M.; Locatis, Craig; Maisiak, Richard; Fontelo, Paul. The effectiveness of mobile phone-based text messaging to intervene with problem drinking in youth and younger adult population: A meta-analysis. 2020. DOI: 10.1089/tmj.2018.0307
3. Alexander, C.N.; Robinson, P.; Rainforth, M.. Treating and preventing alcohol, nicotine, and drug abuse through transcendental meditation: A review and statistical meta-analysis 1994. DOI:
4. Subhani M; Knight H; Ryder S; Morling JR. Does Advice Based on Biomarkers of Liver Injury or Non-Invasive Tests of Liver Fibrosis Impact High-Risk Drinking Behaviour: A Systematic Review With Meta-analysis. 2021. DOI: 10.1093/alcalc/agaa143
5. Keurhorst M; van de Glind I; Bitarello do Amaral-Sabadini M; Anderson P; Kaner E; Newbury-Birch D; Braspenning J; Wensing M; Heinen M; Laurant M. Implementation strategies to enhance management of heavy alcohol consumption in primary health care: a meta-analysis. 2015. DOI: 10.1111/add.13088
6. Kelly JF; Humphreys K; Ferri M. Alcoholics Anonymous and other 12-step programs for alcohol use disorder. 2020. DOI: 10.1002/14651858.CD012880.pub2
7. Smit E; Verdurmen J; Monshouwer K; Smit F. Family interventions and their effect on adolescent alcohol use in general populations; a meta-analysis of randomized controlled trials. 2008. DOI: 10.1016/j.drugalcdep.2008.03.032
8. Calverley, H.L.M.; Petrass, L.A.; Blitvich, J.D.. A systematic review of alcohol education programs for young people: do these programs change behavior? 2021. DOI: 10.1093/her/cyaa049
9. Pot AL; Le Faou AL; Airagnes G; Limosin F. [Not Available]. 2020. DOI: 10.3917/spub.204.0315
10. Jensen, C.D.; Cushing, C.C.; Aylward, B.S.; Craig, J.T.; Sorell, D.M.; Steele, R.G.. Effectiveness of motivational interviewing interventions for adolescent substance use behavior change: A meta-analytic review 2011. DOI: 10.1037/a0023992
11. Magill M; Ray LA. Cognitive-behavioral treatment with adult alcohol and illicit drug users: a meta-analysis of randomized controlled trials. 2009. DOI: 10.15288/jsad.2009.70.516
12. Black N; Mullan B; Sharpe L. Computer-delivered interventions for reducing alcohol consumption: meta-analysis and meta-regression using behaviour change techniques and theory. 2016. DOI: 10.1080/17437199.2016.1168268
13. Garnett C; Crane D; Brown J; Kaner E; Beyer F; Muirhead C; Hickman M; Redmore J; de Vocht F; Beard E; Michie S. Reported Theory Use by Digital Interventions for Hazardous and Harmful Alcohol Consumption, and Association With Effectiveness: Meta-Regression. 2018. DOI: 10.2196/jmir.8807
14. Cadigan JM; Haeny AM; Martens MP; Weaver CC; Takamatsu SK; Arterberry BJ. Personalized drinking feedback: A meta-analysis of in-person versus computer-delivered interventions. 2015. DOI: 10.1037/a0038394
15. Dai W; Palmer R; Sunderrajan A; Durantini M; SÃ¡nchez F; Glasman LR; Chen FX; Albarracín D. More behavioral recommendations produce more change: A meta-analysis of efficacy of multibehavior recommendations to reduce nonmedical substance use. 2020. DOI: 10.1037/adb0000586
16. Maisel NC; Blodgett JC; Wilbourne PL; Humphreys K; Finney JW. Meta-analysis of naltrexone and acamprosate for treating alcohol use disorders: when are these medications most helpful? 2013. DOI: 10.1111/j.1360-0443.2012.04054.x
17. Kramer Schmidt L; Bojesen AB; Nielsen AS; Andersen K. Duration of therapy - Does it matter?: A systematic review and meta-regression of the duration of psychosocial treatments for alcohol use disorder. 2018. DOI: 10.1016/j.jsat.2017.11.002
18. Mann K; Torup L; SÃ¸rensen P; Gual A; Swift R; Walker B; van den Brink W. Nalmefene for the management of alcohol dependence: review on its pharmacology, mechanism of action and meta-analysis on its clinical efficacy. 2016. DOI: 10.1016/j.euroneuro.2016.10.008
19. Cuijpers P; Riper H; Lemmers L. The effects on mortality of brief interventions for problem drinking: a meta-analysis. 2004. DOI: 10.1111/j.1360-0443.2004.00778.x
20. Soyka M; Friede M; Schnitker J. Comparing Nalmefene and Naltrexone in Alcohol Dependence: Are there any Differences? Results from an Indirect Meta-Analysis. 2016. DOI: 10.1055/s-0035-1565184
21. Ray LA; Green R; Roche DJO; Magill M; Bujarski S. Naltrexone effects on subjective responses to alcohol in the human laboratory: A systematic review and meta-analysis. 2019. DOI: 10.1111/adb.12747
22. Hendershot CS; Wardell JD; Samokhvalov AV; Rehm J. Effects of naltrexone on alcohol self-administration and craving: meta-analysis of human laboratory studies. 2017. DOI: 10.1111/adb.12425
23. Kelly S; Olanrewaju O; Cowan A; Brayne C; Lafortune L. Interventions to prevent and reduce excessive alcohol consumption in older people: a systematic review and meta-analysis. 2018. DOI: 10.1093/ageing/afx132
24. Muckle W; Muckle J; Welch V; Tugwell P. Managed alcohol as a harm reduction intervention for alcohol addiction in populations at high risk for substance abuse. 2012. DOI: 10.1002/14651858.CD006747.pub2
25. Ahmed R; Kotapati VP; Khan AM; Hussain N; Hussain M; Dar S; Kumar J; Begum GA; Esang M; Brainch N; Ahmed S. Adding Psychotherapy to the Naltrexone Treatment of Alcohol Use Disorder: Meta-analytic Review. 2018. DOI: 10.7759/cureus.3107
26. R:osner S; Leucht S; Lehert P; Soyka M. Acamprosate supports abstinence, naltrexone prevents excessive drinking: evidence from a meta-analysis with unreported outcomes. 2008. DOI: 10.1177/0269881107078308
27. Pribék IK; Kovács I; Kádár BK; Kovács CS; Richman MJ; Janka Z; Andó B; Lázár BA. Evaluation of the course and treatment of Alcohol Withdrawal Syndrome with the Clinical Institute Withdrawal Assessment for Alcohol - Revised: A systematic review-based meta-analysis. 2021. DOI: 10.1016/j.drugalcdep.2021.108536
28. Scott-Sheldon LAJ; Carey KB; Johnson BT; Carey MP. Behavioral Interventions Targeting Alcohol Use Among People Living with HIV/AIDS: A Systematic Review and Meta-Analysis. 2017. DOI: 10.1007/s10461-017-1886-3
29. Ker K; Chinnock P. Interventions in the alcohol server setting for preventing injuries. 2006. DOI: 10.1002/14651858.CD005244.pub2
30. Sileo KM; Miller AP; Wagman JA; Kiene SM. Psychosocial interventions for reducing alcohol consumption in sub-Saharan African settings: a systematic review and meta-analysis. 2021. DOI: 10.1111/add.15227
31. McGovern R; Newham JJ; Addison MT; Hickman M; Kaner EF. Effectiveness of psychosocial interventions for reducing parental substance misuse. 2021. DOI: 10.1002/14651858.CD012823.pub2
32. Srisurapanont M; Jarusuraisin N. Naltrexone for the treatment of alcoholism: a meta-analysis of randomized controlled trials. 2005. DOI: 10.1017/S1461145704004997
33. Latifi S; Messer T. The Efficacy of Tiapride and Carbamazepine Combination Therapy in Reducing Alcohol Withdrawal Symptoms: A Systematic Review and Meta-Analysis. 2019. DOI: 10.1055/a-0795-3689
34. Scott-Sheldon LA; Terry DL; Carey KB; Garey L; Carey MP. Efficacy of expectancy challenge interventions to reduce college student drinking: a meta-analytic review. 2012. DOI: 10.1037/a0027565
35. Steinka-Fry KT; Tanner-Smith EE; Hennessy EA. Effects of Brief Alcohol Interventions on Drinking and Driving among Youth: A Systematic Review and Meta-analysis. 2015. DOI: 10.13188/2330-2178.1000016
36. Dotson KB; Dunn ME; Bowers CA. Stand-Alone Personalized Normative Feedback for College Student Drinkers: A Meta-Analytic Review, 2004 to 2014. 2015. DOI: 10.1371/journal.pone.0139518
37. Tanner-Smith EE; Risser MD. A meta-analysis of brief alcohol interventions for adolescents and young adults: variability in effects across alcohol measures. 2016. DOI: 10.3109/00952990.2015.1136638
38. Magill, M.; Ray, L.A.; Kiluk, B.; Hoadley, A.; Bernstein, M.; Tonigan, J.S.; Carroll, K.. A meta-analysis of cognitive-behavioral therapy for alcohol or other drug use disorders: Treatment efficacy by contrast condition 2019. DOI: 10.1111/acer.14059
39. Tripodi SJ; Bender K; Litschge C; Vaughn MG. Interventions for reducing adolescent alcohol abuse: a meta-analytic review. 2010. DOI: 10.1001/archpediatrics.2009.235
40. Couzigou P; Vergniol J; Kowo M; Terrebonne E; Foucher J; Castera L; Laharie D; De Ledinghen V. [Brief intervention about alcohol use]. 2009. DOI: 10.1016/j.lpm.2008.09.026
41. Ballesteros J; Arino J; González-Pinto A; Querejetad I. [Effectiveness of medical advice for reducing excessive alcohol consumption. Meta-analysis of Spanish studies in primary care]. 2003. DOI: 10.1016/s0213-9111(03)71708-7
42. Hennessy EA; Tanner-Smith EE; Steinka-Fry KT. Do brief alcohol interventions reduce tobacco use among adolescents and young adults? A systematic review and meta-analysis. 2015. DOI: 10.1007/s10865-015-9653-2
43. Riper H; Andersson G; Hunter SB; de Wit J; Berking M; Cuijpers P. Treatment of comorbid alcohol use disorders and depression with cognitive-behavioural therapy and motivational interviewing: a meta-analysis. 2014. DOI: 10.1111/add.12441
44. Carey KB; Scott-Sheldon LA; Garey L; Elliott JC; Carey MP. Alcohol interventions for mandated college students: A meta-analytic review. 2016. DOI: 10.1037/a0040275
45. Blodgett JC; Del Re AC; Maisel NC; Finney JW. A meta-analysis of topiramate's effects for individuals with alcohol use disorders. 2014. DOI: 10.1111/acer.12411
46. Gimeno C; Dorado ML; Roncero C; Szerman N; Vega P; Balanzá-Martínez V; Alvarez FJ. Treatment of Comorbid Alcohol Dependence and Anxiety Disorder: Review of the Scientific Evidence and Recommendations for Treatment. 2017. DOI: 10.3389/fpsyt.2017.00173
47. Burns E; Gray R; Smith LA. Brief screening questionnaires to identify problem drinking during pregnancy: a systematic review. 2010. DOI: 10.1111/j.1360-0443.2009.02842.x
48. Herremans SC; Baeken C. The current perspective of neuromodulation techniques in the treatment of alcohol addiction: a systematic review. 2012. DOI:
49. Awad C; Garceau V; Poisson C. Efficacité et innocuité des traitements pharmacologiques pour le sevrage d’alcool et la prévention des rechutes : Rapport de revues systématiques 2021. DOI:
50. Shin NY; Lim YJ; Yang CH; Kim C. Acupuncture for Alcohol Use Disorder: A Meta-Analysis. 2017. DOI: 10.1155/2017/7823278
51. Whitworth M; Dowswell T. Routine pre-pregnancy health promotion for improving pregnancy outcomes. 2009. DOI: 10.1002/14651858.CD007536.pub2
52. Savic M; Best D; Manning V; Lubman DI. Strategies to facilitate integrated care for people with alcohol and other drug problems: a systematic review. 2017. DOI: 10.1186/s13011-017-0104-7
53. Petry, N.M.. The need for combining pharmacotherapy and psychotherapy in treating substance abuse and gambling disorders 2001. DOI:
54. Sofuoglu, M.; Rosenheck, R.; Petrakis, I.. Pharmacological treatment of comorbid PTSD and substance use disorder: Recent progress 2014. DOI: 10.1016/j.addbeh.2013.08.014
55. Schmidt CS; Schulte B; Seo HN; Kuhn S; O'Donnell A; Kriston L; Verthein U; Reimer J. Meta-analysis on the effectiveness of alcohol screening with brief interventions for patients in emergency care settings. 2016. DOI: 10.1111/add.13263
56. Lum E; Gorman SK; Slavik RS. Valproic acid management of acute alcohol withdrawal. 2006. DOI: 10.1345/aph.1G243
57. Thompson TP; Taylor AH; Wanner A; Husk K; Wei Y; Creanor S; Kandiyali R; Neale J; Sinclair J; Nasser M; Wallace G. Physical activity and the prevention, reduction, and treatment of alcohol and/or substance use across the lifespan (The PHASE review): protocol for a systematic review. 2018. DOI: 10.1186/s13643-018-0674-0
58. GÃ¼nthner, A.. Evidence based psychotherapy of alcohol-dependent patients: Are there integrative approaches which are practicable? 2002. DOI: 10.1024/suc.2002.48.3.158
59. Anderson P. Overview of interventions to enhance primary-care provider management of patients with substance-use disorders. 2009. DOI: 10.1111/j.1465-3362.2009.00113.x
60. Kelly-Weeder S. Binge drinking in college-aged women: framing a gender-specific prevention strategy. 2008. DOI: 10.1111/j.1745-7599.2008.00357.x
61. Nilsen, P.. Brief alcohol intervention to prevent drinking during pregnancy: an overview of research findings. 2009. DOI:
62. Muzyk AJ; Kerns S; Brudney S; Gagliardi JP. Dexmedetomidine for the treatment of alcohol withdrawal syndrome: rationale and current status of research. 2013. DOI: 10.1007/s40263-013-0106-6
63. Ralevski E; Olivera-Figueroa LA; Petrakis I. PTSD and comorbid AUD: a review of pharmacological and alternative treatment options. 2014. DOI: 10.2147/SAR.S37399
64. Mayo-Smith, M.F.. Pharmacological management of alcohol withdrawal: A meta-analysis and evidence-based practice guideline 1997. DOI: 10.1001/jama.278.2.144
65. Winslow, B.T.; Onysko, M.; Hebert, M.. Medications for alcohol use disorder 2016. DOI:
66. Saunders, J.; Kypri, K.. Effectiveness of brief alcohol interventions: Evidence from studies of academic attainment, social problems and morbidity and mortality 2012. DOI: 10.1111/j.1530-0277.2012.01917.x
67. Brathen, G.; Ben-Menachem, E.; Brodtkorb, E.; Hillbom, M.E.; Jesse, S.; Keindl, M.; Ludolph, A.C.; Tanasescu, R.; Leone, M.. Alcohol-withdrawal seizures 2016. DOI: 10.1111/ene.13096
68. Glahn A; Proskynitopoulos PJ; Bleich S; Hillemacher T. Pharmacotherapeutic management of acute alcohol withdrawal syndrome in critically Ill patients. 2020. DOI: 10.1080/14656566.2020.1746271
69. Cooper E; Vernon J. The effectiveness of pharmacological approaches in the treatment of alcohol withdrawal syndrome (AWS): a literature review. 2013. DOI: 10.1111/j.1365-2850.2012.01958.x
70. Nilsen P; Aalto M; Bendtsen P; SeppÃ¤ K. Effectiveness of strategies to implement brief alcohol intervention in primary healthcare. A systematic review. 2006. DOI: 10.1080/02813430500475282
71. Prendergast M; Podus D; Finney J; Greenwell L; Roll J. Contingency management for treatment of substance use disorders: a meta-analysis. 2006. DOI: 10.1111/j.1360-0443.2006.01581.x
72. Beyer F; Lynch E; Kaner E. Brief Interventions in Primary Care: an Evidence Overview of Practitioner and Digital Intervention Programmes. 2018. DOI: 10.1007/s40429-018-0198-7
73. McMurran M. Individual-level interventions for alcohol-related violence: a rapid evidence assessment. 2012. DOI: 10.1002/cbm.821
74. Leone, M.A.; Vigna-Taglianti, F.; Avanzi, G.; Brambilla, R.; Faggiano, F.. Gamma-hydroxybutyrate for treatment of alcohol withdrawal and prevention of relapses: A Cochrane review 2010. DOI: 10.1111/j.1468-1331.2010.03232.x
75. Pereira MO; Anginoni BM; Ferreira Nda C; de Oliveira MA; de Vargas D; Colvero Lde A. [Effectiveness of the brief intervention for the use of abusive alcohol in primary care: systematic review]. 2013. DOI: 10.1590/s0034-71672013000300018
76. van den Brink W. Evidence-based pharmacological treatment of substance use disorders and pathological gambling. 2012. DOI: 10.2174/1874473711205010003
77. Tanner-Smith EE; Steinka-Fry KT; Hennessy EA; Lipsey MW; Winters KC. Can brief alcohol interventions for youth also address concurrent illicit drug use? results from a meta-analysis. 2015. DOI: 10.1007/s10964-015-0252-x
78. Tolliver, B.K.. Dysregulation of glutamate neurotransmission as a potential therapeutic target in bipolar disorder and comorbid alcohol dependence: A review of current evidence 2010. DOI: 10.1111/j.1399-5618.2010.00782.x
79. Segawa, T.; Baudry, T.; Bourla, A.; Blanc, J.-V.; Peretti, C.-S.; Mouchabac, S.; Ferreri, F.. Virtual Reality (VR) in Assessment and Treatment of Addictive Disorders: A Systematic Review 2020. DOI: 10.3389/fnins.2019.01409
80. Modesto-Lowe, V.; Boornazian, A.. Screening and brief intervention in the management of early problem drinkers: Integration into healthcare settings 2000. DOI: 10.2165/00115677-200008030-00002
81. Garcia-Romeu, Albert; Davis, Alan K.; Erowid, Fire; Erowid, Earth; Griffiths, Roland R.; Johnson, Matthew W.. Cessation and reduction in alcohol consumption and misuse after psychedelic use. 2019. DOI: 10.1177/0269881119845793
82. Shinn AK; Greenfield SF. Topiramate in the treatment of substance-related disorders: a critical review of the literature. 2010. DOI: 10.4088/JCP.08r04062gry
83. Tanner-Smith EE; Lipsey MW. Brief alcohol interventions for adolescents and young adults: a systematic review and meta-analysis. 2015. DOI: 10.1016/j.jsat.2014.09.001
84. Vasilaki EI; Hosier SG; Cox WM. The efficacy of motivational interviewing as a brief intervention for excessive drinking: a meta-analytic review. 2006. DOI: 10.1093/alcalc/agl016
85. Hulse, G.K.. Improving clinical outcomes for naltrexone as a management of problem alcohol use 2013. DOI: 10.1111/j.1365-2125.2012.04452.x
86. Roberts NP; Roberts PA; Jones N; Bisson JI. Psychological interventions for post-traumatic stress disorder and comorbid substance use disorder: A systematic review and meta-analysis. 2015. DOI: 10.1016/j.cpr.2015.02.007
87. Samson JE; Tanner-Smith EE. Single-Session Alcohol Interventions for Heavy Drinking College Students: A Systematic Review and Meta-Analysis. 2015. DOI: 10.15288/jsad.2015.76.530
88. Shorter D; Hsieh J; Kosten TR. Pharmacologic management of comorbid post-traumatic stress disorder and addictions. 2015. DOI: 10.1111/ajad.12306
89. Martin GW; Rehm J. The effectiveness of psychosocial modalities in the treatment of alcohol problems in adults: a review of the evidence. 2012. DOI: 10.1177/070674371205700604
90. Olmsted CL; Kockler DR. Topiramate for alcohol dependence. 2008. DOI: 10.1345/aph.1L157
91. Weresch, J.; Kirkwood, J.; Korownyk, C.S.. Gabapentin for alcohol use disorder 2021. DOI: 10.46747/cfp.6704269
92. Murphy, C.M.; MacKillop, J.; Miranda, R.; Meehan, J.; Swift, R.W.; Monti, P.M.. A dose-based meta-analysis of topiramate's effects on alcohol consumption and heavy drinking 2011. DOI: 10.1111/j.1530-0277.2011.01497.x
93. Field, C.. A compartive effectiveness trial of three brief intervention strategies to reduce alcohol use after injury 2016. DOI: 10.1111/acer.13085
94. Mun, E.Y.; Garfinkle, E.. Are there intervention effects on protective behavioral strategies and alcohol-related problems for college students? a multivariate meta analysis 2016. DOI: 10.1111/acer.13084
95. Havard, A.; Shakeshaft, A.; Sanson-Fisher, R.. Systematic review and meta-analyses of strategies targeting alcohol problems in emergency departments: Interventions reduce alcohol-related injuries 2008. DOI: 10.1111/j.1360-0443.2007.02072.x
96. Vuittonet CL; Halse M; Leggio L; Fricchione SB; Brickley M; Haass-Koffler CL; Tavares T; Swift RM; Kenna GA. Pharmacotherapy for alcoholic patients with alcoholic liver disease. 2014. DOI: 10.2146/ajhp140028
97. Tait RJ; Christensen H. Internet-based interventions for young people with problematic substance use: a systematic review. 2010. DOI: 10.5694/j.1326-5377.2010.tb03687.x
98. Thibaut F; Chagraoui A; Buckley L; Gressier F; Labad J; Lamy S; Potenza MN; Rondon M; Riecher-Rössler A; Soyka M; Yonkers K. WFSBP (*) and IAWMH (**) Guidelines for the treatment of alcohol use disorders in pregnant women. 2019. DOI: 10.1080/15622975.2018.1510185
99. Soyka M; Rösner S. Emerging drugs to treat alcoholism. 2010. DOI: 10.1517/14728214.2010.500811
100. Nowzari, S.; Finnell, D.S.; Broyles, L.M.. Nurse provision of alcohol-related interventions in the patient-centered medical home 2013. DOI: 10.1111/acer.12162
101. Oliva, E.M.; Harris, A.H.S.. Systematic review and meta-analysis: If pharmacotherapies for alcohol use disorders are effective, why are they underutilised? 2014. DOI: 10.1136/ebmed-2014-110050
102. Roche AM; Freeman T; Skinner N. From data to evidence, to action: findings from a systematic review of hospital screening studies for high risk alcohol consumption. 2006. DOI: 10.1016/j.drugalcdep.2005.10.011
103. Nöhles, V.B.; Correll, C.U.; Roldan, A.; Galling, B.. Efficacy and safety of adjunctive pharmacologic treatments for alcohol addiction in patients with schizophrenia and bipolar disorder 2019. DOI: 10.1016/j.euroneuro.2018.11.725
104. Andrade, C.. Gabapentin for Alcohol-Related Disorders: Critical Appraisal of the Symptom-Driven Approach 2020. DOI: 10.4088/JCP.20f13775
105. Oetzel S. [Drug therapy supports reduction of alcohol consumption]. 2014. DOI:
106. . Pharmacotherapy for Adults With Alcohol Use Disorder in Outpatient Settings. 2011. DOI:
107. Hansen, William B.; Derzon, James; Dusenbury, Linda; Bishop, Dana; Campbell, Karren; Alford, Aaron. Operating characteristics of prevention programs: Connections to drug use etiology. 2010. DOI:
108. Kaur, J.. Off-label use of medications for alcohol use disorder 2019. DOI: 10.1002/ajad.12887
109. Takano A; Miyamoto Y; Matsumoto T. [A review about new approaches using the Internet and computer technology for people with drug use disorder]. 2015. DOI:
110. Lebiecka Z; Skoneczny T; Tyburski E; Samochowiec J; Kucharska-Mazur J. Is Virtual Reality Cue Exposure a Promising Adjunctive Treatment for Alcohol Use Disorder? 2021. DOI: 10.3390/jcm10132972
111. Ahankari AS; Wray J; Jomeen J; Hayter M. The effectiveness of combined alcohol and sexual risk taking reduction interventions on the sexual behaviour of teenagers and young adults: a systematic review. 2019. DOI: 10.1016/j.puhe.2019.05.023
112. Wong A; Smithburger PL; Kane-Gill SL. Review of adjunctive dexmedetomidine in the management of severe acute alcohol withdrawal syndrome. 2015. DOI: 10.3109/00952990.2015.1058390
113. Gordon, A.J.. Screening the drinking: Identifying problem alcohol consumption in primary care settings 2006. DOI:
114. Ullman, S.E.; Najdowski, C.J.; Adams, E.B.. Women, alcoholics anonymous, and related mutual aid groups: Review and recommendations for research 2012. DOI: 10.1080/07347324.2012.718969
115. Ray LA; Du H; Green R; Roche DJO; Bujarski S. Do behavioral pharmacology findings predict clinical trial outcomes? A proof-of-concept in medication development for alcohol use disorder. 2021. DOI: 10.1038/s41386-020-00913-3
116. Arbaizar B; Diersen-Sotos T; Gómez-Acebo I; Llorca J. Topiramate in the treatment of alcohol dependence: a meta-analysis. 2010. DOI:
117. Soyka M; Kranzler HR; Hesselbrock V; Kasper S; Mutschler J; Müller HJ. Guidelines for biological treatment of substance use and related disorders, part 1: Alcoholism, first revision. 2017. DOI: 10.1080/15622975.2016.1246752
118. Deschner, E.; Walsh, C.; Spithoff, S.; McLeod, S.; Borgundvaag, B.; Bearss, E.; Foote, J.; Gravel, J.. Naltrexone initiation for alcohol use disorder in the emergency department: A systematic review 2019. DOI: 10.1017/cem.2019.222
119. Drummond DC. Alcohol interventions: do the best things come in small packages? 1997. DOI:
120. Reid AE; Carey KB. Interventions to reduce college student drinking: State of the evidence for mechanisms of behavior change. 2015. DOI: 10.1016/j.cpr.2015.06.006
121. Scharer, J.L.; Funderburk, J.S.; De Vita, M.J.; Rother, Y.; Maisto, S.A.. Treatment as usual control groups in brief alcohol intervention trials: A systematic review and meta-analysis 2021. DOI: 10.1111/acer.14628
122. Kim Y; Hack LM; Ahn ES; Kim J. Practical outpatient pharmacotherapy for alcohol use disorder. 2018. DOI: 10.7573/dic.212308
123. Caputo, F.; Bernardi, M.. Sodium oxybate to treat alcohol dependence: 20 years of clinical experience 2013. DOI: 10.1111/adb.12113
124. Buonopane, A.; Petrakis, I.L.. Pharmacotherapy of alcohol use disorders 2005. DOI: 10.1080/10826080500294890
125. Edwards AG; Rollnick S. Outcome studies of brief alcohol intervention in general practice: the problem of lost subjects. 1997. DOI:
126. Soyka M; Kranzler HR; Berglund M; Gorelick D; Hesselbrock V; Johnson BA; MÃ¶ller HJ. World Federation of Societies of Biological Psychiatry (WFSBP) Guidelines for Biological Treatment of Substance Use and Related Disorders, Part 1: Alcoholism. 2008. DOI: 10.1080/15622970801896390
127. Anderson P; O'Donnell A; Kaner E. Managing Alcohol Use Disorder in Primary Health Care. 2017. DOI: 10.1007/s11920-017-0837-z
128. Lupi M; Martinotti G; Santacroce R; Cinosi E; Carlucci M; Marini S; Acciavatti T; di Giannantonio M. Transcranial Direct Current Stimulation in Substance Use Disorders: A Systematic Review of Scientific Literature. 2017. DOI: 10.1097/YCT.0000000000000401
129. Garbutt, J.C.; Greenblatt, A.; West, S.; Morgan, L.; Kampov-Polevoy, A.; Jordan, H.; Bobashev, G.. Potential predictors of response to naltrexone in alcohol dependence: A systematic reviewof theworld literature 2013. DOI: 10.1111/acer.12162
130. Erwin BL; Slaton RM. Varenicline in the treatment of alcohol use disorders. 2014. DOI: 10.1177/1060028014545806
131. Ahmed, S.; Haggerty, G.; Khan, A.M.; Esang, M.; Mekala, H.M.; Qamar, I.. The effectiveness of gabapentin in reducing cravings and withdrawal in alcohol dependence: A meta-analytic review 2018. DOI: 10.1111/ajad.12753
132. Garbutt, J.; Greenblatt, A.; West, S.L.; Morgan, L.; Kampov-Polevoy, A.; Jordan, H.; Bobashev, G.. Potential predictors of naltrexone response in alcohol dependence: A systematic review 2013. DOI: 10.1002/pds.3512
133. Freynhagen R; Backonja M; Schug S; Lyndon G; Parsons B; Watt S; Behar R. Pregabalin for the Treatment of Drug and Alcohol Withdrawal Symptoms: A Comprehensive Review. 2016. DOI: 10.1007/s40263-016-0390-z
134. Solberg LI; Maciosek MV; Edwards NM. Primary care intervention to reduce alcohol misuse ranking its health impact and cost effectiveness. 2008. DOI: 10.1016/j.amepre.2007.09.035
135. Muzyk AJ; Fowler JA; Norwood DK; Chilipko A. Role of Î±2-agonists in the treatment of acute alcohol withdrawal. 2011. DOI: 10.1345/aph.1P575
136. Ballesteros J; González-Pinto A; Querejeta I; Ariño J. Brief interventions for hazardous drinkers delivered in primary care are equally effective in men and women. 2004. DOI: 10.1111/j.1360-0443.2004.00499.x
137. Luty, J.. What works in alcohol use disorders? 2006. DOI: 10.1192/apt.12.1.13
138. Wong S; Ordean A; Kahan M. Substance use in pregnancy. 2011. DOI: 10.1016/S1701-2163(16)34855-1
139. Wilde MI; Wagstaff AJ. Acamprosate. A review of its pharmacology and clinical potential in the management of alcohol dependence after detoxification. 1997. DOI: 10.2165/00003495-199753060-00008
140. Plosker GL. Acamprosate: A Review of Its Use in Alcohol Dependence. 2015. DOI: 10.1007/s40265-015-0423-9
141. Kennedy WK; Leloux M; Kutscher EC; Price PL; Morstad AE; Carnahan RM. Acamprosate. 2010. DOI: 10.1517/17425251003641975
142. Thatcher, D.L.; Clark, D.B.. Adolescent alcohol abuse and dependence: Development, diagnosis, treatment and outcomes 2006. DOI: 10.2174/157340006775101544
143. Glynn, T.R.; Van Den Berg, J.J.. A Systematic Review of Interventions to Reduce Problematic Substance Use among Transgender Individuals: A Call to Action 2017. DOI: 10.1089/trgh.2016.0037
144. Marin Mayor, M.; Lopez Alvarez, J.; Riaza Perez, M.D.; Quintana Perez, A.; Rubio Valladolid, G.. Use of anticonvulsant agents in the management of alcohol dependence 2011. DOI: 10.1016/S0924-9338(11)71789-8
145. Jordan CO; Slater M; Kottke TE. Preventing chronic disease risk factors: rationale and feasibility. 2008. DOI:
146. Jiloha RC. Prevention, early intervention, and harm reduction of substance use in adolescents. 2017. DOI: 10.4103/0019-5545.204444
147. Mayo-Smith MF. Pharmacological management of alcohol withdrawal. A meta-analysis and evidence-based practice guideline. American Society of Addiction Medicine Working Group on Pharmacological Management of Alcohol Withdrawal. 1997. DOI: 10.1001/jama.278.2.144
148. Graves L; Carson G; Poole N; Patel T; Bigalky J; Green CR; Cook JL. Guideline No. 405: Screening and Counselling for Alcohol Consumption During Pregnancy. 2020. DOI: 10.1016/j.jogc.2020.03.002
149. Imel ZE; Wampold BE; Miller SD; Fleming RR. Distinctions without a difference: direct comparisons of psychotherapies for alcohol use disorders. 2008. DOI: 10.1037/a0013171
150. Batel P. The treatment of alcoholism in France. 1995. DOI: 10.1016/0376-8716(95)01169-y
151. Agabio, R.; Preti, A.; Gessa, G.L.. Efficacy and tolerability of baclofen in substance use disorders: A systematic review 2013. DOI: 10.1159/000347055
152. Addolorato, G.; Mirijello, A.; Leggio, L.; Ferrulli, A.; Landolfi, R.. Management of alcohol dependence in patients with liver disease 2013. DOI: 10.1007/s40263-013-0043-4
153. Aboujaoude E; Salame WO. Naltrexone: A Pan-Addiction Treatment? 2016. DOI: 10.1007/s40263-016-0373-0
154. Fonagy, P.; Roth, A.; Higgitt, A.. Psychodynamic psychotherapies: Evidence-based practice and clinical wisdom 2005. DOI: 10.1521/bumc.69.1.1.62267
155. Koopmann A; Schuster R; Kiefer F. The impact of the appetite-regulating, orexigenic peptide ghrelin on alcohol use disorders: A systematic review of preclinical and clinical data. 2018. DOI: 10.1016/j.biopsycho.2016.12.012
156. Mason, B.J.; Ownby, R.L.. Acamprosate for the treatment of alcohol dependence: A review of double- blind, placebo-controlled trials 2000. DOI: 10.1017/S1092852900012827
157. Overman GP; Teter CJ; Guthrie SK. Acamprosate for the adjunctive treatment of alcohol dependence. 2003. DOI: 10.1345/aph.1C351
158. Anderson P; Scafato E; Galluzzo L. Alcohol and older people from a public health perspective. 2012. DOI: 10.4415/ANN_12_03_04
159. Mutschler J; Soyka M. [Pharmacological prophylactic treatment for relapse of alcohol dependence : Results of current meta-analyses]. 2017. DOI: 10.1007/s00115-016-0133-x
160. Miller MB; Leffingwell T; Claborn K; Meier E; Walters S; Neighbors C. Personalized feedback interventions for college alcohol misuse: an update of Walters & Neighbors (2005). 2013. DOI: 10.1037/a0031174
161. Mun, E.Y.; White, H.E.; De La Torre, J.; Atkins, D.C.; Larimer, M.E.; Jiao, Y.; Huo, Y.; Garberson, L. Overview of integrative analysis of brief alcohol interventions for college students 2011. DOI: 10.1111/j.1530-0277.2011.01497.x
162. Hattingh HL; Tait RJ. Pharmacy-based alcohol-misuse services: current perspectives. 2017. DOI: 10.2147/IPRP.S140431
163. Clark DB; Gordon AJ; Ettaro LR; Owens JM; Moss HB. Screening and brief intervention for underage drinkers. 2010. DOI: 10.4065/mcp.2008.0638
164. Swift R; Oslin DW; Alexander M; Forman R. Adherence monitoring in naltrexone pharmacotherapy trials: a systematic review. 2011. DOI: 10.15288/jsad.2011.72.1012
165. Keleti, D.; Golinkoff, M.; Weaver, K.; Michael, K.E.; Gelzer, A.D.. Metareview of findings in existing literature reviews covering behavioral health-physical health integration studies 2015. DOI:
166. Irvin JE; Bowers CA; Dunn ME; Wang MC. Efficacy of relapse prevention: a meta-analytic review. 1999. DOI: 10.1037//0022-006x.67.4.563
167. Allen, J.P.; Litten, R.Z.. Alcoholics with collateral psychopathology: Issues and research findings 1998. DOI:
168. Klemperer, E.M.; Hughes, J.. Does the efficacy of medications for substance abuse treatment decrease over time? 2015. DOI: 10.1016/j.drugalcdep.2014.09.353
169. Allen, M.L.; Garcia-Huidobro, D.; Curran, D.. Parent training interventions to prevent adolescent substance use: A systematic review 2015. DOI: 10.1016/j.jadohealth.2014.10.240
170. Anderson P; Laurant M; Kaner E; Wensing M; Grol R. Engaging general practitioners in the management of hazardous and harmful alcohol consumption: results of a meta-analysis. 2004. DOI: 10.15288/jsa.2004.65.191
171. Garbutt JC. Efficacy and tolerability of naltrexone in the management of alcohol dependence. 2010. DOI: 10.2174/138161210791516459
172. Berner M; GÃ¼nzler C; Frick K; Kriston L; Loessl B; Bräck R; Gann H; Batra A; Mann K. Finding the ideal place for a psychotherapeutic intervention in a stepped care approach--a brief overview of the literature and preliminary results from the Project PREDICT. 2008. DOI: 10.1002/mpr.250
173. Evren, C.; Bozkurt, M.. Pharmacological treatment options for alcohol use disorder 2015. DOI: 10.5350/DAJPN20152804001
174. Batel, P.. The treatment of alcoholism in France 1995. DOI: 10.1016/0376-8716(95)01169-Y
175. Kelleher DC; Renaud EJ; Ehrlich PF; Burd RS. Guidelines for alcohol screening in adolescent trauma patients: a report from the Pediatric Trauma Society Guidelines Committee. 2013. DOI: 10.1097/TA.0b013e31827d5f80
176. McCambridge, J.; Kypri, K.. Does simply asking questions change behavior? A systematic review of brief alcohol intervention trial data 2009. DOI: 10.1111/j.1530-0277.2009.00948.x
177. Wright, T.M.; Myrick, H.. Acamprosate: A new tool in the battle against alcohol dependence 2006. DOI: 10.2147/nedt.2006.2.4.445
178. Hunt GE; Siegfried N; Morley K; Sitharthan T; Cleary M. Psychosocial interventions for people with both severe mental illness and substance misuse. 2013. DOI: 10.1002/14651858.CD001088.pub3
179. Montag A; Clapp JD; Calac D; Gorman J; Chambers C. A review of evidence-based approaches for reduction of alcohol consumption in Native women who are pregnant or of reproductive age. 2012. DOI: 10.3109/00952990.2012.694521
180. Shamblen SR; Derzon JH. A preliminary study of the population-adjusted effectiveness of substance abuse prevention programming: towards making IOM program types comparable. 2009. DOI: 10.1007/s10935-009-0168-x
181. Taylor M; Petrakis I; Ralevski E. Treatment of alcohol use disorder and co-occurring PTSD. 2017. DOI: 10.1080/00952990.2016.1263641
182. Melendez-Torres GJ; Tancred T; Fletcher A; Thomas J; Campbell R; Bonell C. Does integrated academic and health education prevent substance use? Systematic review and meta-analyses. 2018. DOI: 10.1111/cch.12558
183. Nadkarni A; Costa S; Gupta D; Fernandes D; Catalano A; Velleman R; Sambari S; Pednekar S; Hussain F; D'Souza E; Houde A; Chaudhuri N; Heath A. The systematic development of a mobile phone-delivered brief intervention for hazardous drinking in India. 2021. DOI: 10.1016/j.jsat.2021.108331
184. Luty, J.. Drug and alcohol addiction: Do psychosocial treatments work? 2015. DOI: 10.1192/apt.bp.114.013177
185. Secades-Álvarez A; Fernández-Rodríguez C. Review of the efficacy of treatments for bipolar disorder and substance abuse. 2017. DOI: 10.1016/j.rpsm.2015.10.003
186. Liddle, H.A.. Family-based therapies for adolescent alcohol and drug use: Research contributions and future research needs 2004. DOI: 10.1111/j.1360-0443.2004.00856.x
187. Boekeloo, B.O.; Griffin, M.A.. Review of clinical trials testing the effectiveness of physician approaches to improving alcohol education and counseling in adolescent outpatients 2007. DOI: 10.2174/157339607779941679
188. Kingsland M; Wiggers J; Wolfenden L. Interventions in sports settings to reduce alcohol consumption and alcohol-related harm: a systematic review protocol. 2012. DOI: 10.1136/bmjopen-2011-000645
189. Blodgett, J.C.; Maisel, N.C.; Fuh, I.L.; Wilbourne, P.L.; Finney, J.W.. How effective is continuing care for substance use disorders? A metaanalytic review 2012. DOI: 10.1111/j.1530-0277.2012.01803.x
190. Tobin, J.; Delaney, W.; Doyle, H.. The controlled drinking controversy 1993. DOI:
191. Rombouts SA; Conigrave J; Louie E; Haber P; Morley KC. Evidence-based models of care for the treatment of alcohol use disorder in primary health care settings: protocol for systematic review. 2019. DOI: 10.1186/s13643-019-1157-7
192. Berglund M. A better widget? Three lessons for improving addiction treatment from a meta-analytical study. 2005. DOI: 10.1111/j.1360-0443.2005.01106.x
193. Mun EY; Li X; Lineberry S; Tan Z; Huh D; Walters ST; Zhou Z; Larimer ME. Do Brief Alcohol Interventions Reduce Driving After Drinking Among College Students? A Two-step Meta-analysis of Individual Participant Data. 2021. DOI: 10.1093/alcalc/agaa146
194. Fiore M; Torretta G; Passavanti MB; Sansone P; Pace MC; Alfieri A; Aurilio C; Simeon V; Chiodini P; Pota V. Dexmedetomidine as adjunctive therapy for the treatment of alcohol withdrawal syndrome: a systematic review protocol. 2019. DOI: 10.11124/JBISRIR-2017-003949
195. Hale DR; Fitzgerald-Yau N; Viner RM. A systematic review of effective interventions for reducing multiple health risk behaviors in adolescence. 2014. DOI: 10.2105/AJPH.2014.301874
196. Karyadi, K.A.; Vanderveen, D.; Cyders, M.A.. A meta-analysis of the relationship between mindfulness and substance use behaviors 2014. DOI:
197. Adelman-Mullally T; Kerber C; Reitz OE; Kim M. Alcohol Abstinence Self-Efficacy and Recovery Using Alcoholics Anonymous(Â®) An Integrative Review of the Literature. 2021. DOI: 10.3928/02793695-20210324-05
198. Schmidt, F.L.; Viswesvaran, V.; Ones, D.S.. Validity of integrity tests for predicting drug and alcohol abuse: a meta-analysis. 1997. DOI:
199. Arranz B; Garriga M; García-Rizo C; San L. Clozapine use in patients with schizophrenia and a comorbid substance use disorder: A systematic review. 2018. DOI: 10.1016/j.euroneuro.2017.12.006
200. Dale E; Kelly PJ; Lee KSK; Conigrave JH; Ivers R; Clapham K. Systematic review of addiction recovery mutual support groups and Indigenous people of Australia, New Zealand, Canada, the United States of America and Hawaii. 2019. DOI: 10.1016/j.addbeh.2019.106038
201. Bernstein JA; Bernstein E; Heeren TC. Mechanisms of change in control group drinking in clinical trials of brief alcohol intervention: implications for bias toward the null. 2010. DOI: 10.1111/j.1465-3362.2010.00174.x
202. Timko C; Kong C; Vittorio L; Cucciare MA. Screening and brief intervention for unhealthy substance use in patients with chronic medical conditions: a systematic review. 2016. DOI: 10.1111/jocn.13244
203. Bujarski, S.; Green, R.; Roche, D.J.O.; Ray, L.A.. Naltrexone and subjective responses to alcohol: A systematic reviewand meta-analysis 2017. DOI: 10.1111/acer.13391
204. Protogerou C; McHugh RK; Johnson BT. How best to reduce unhealthy risk-taking behaviours? A meta-review of evidence syntheses of interventions using self-regulation principles. 2020. DOI: 10.1080/17437199.2019.1707104
205. Benedetti, A.; Saxton, M.; Schyma, B.. Strategies targeting alcohol use in trauma patients injured while under the influence: A systematic review 2020. DOI: 10.1177/1460408620948658
206. Beresford, T.P.. Medications for alcohol use disorders 2014. DOI: 10.1001/jama.2014.10161
207. Lipsey, M.W.. Variability across treatments and outcomes: Meta-analysis of the effects ofadolescent substance abuse treatment 2012. DOI: 10.1111/j.1530-0277.2012.01804.x
208. Rosato V; Abenavoli L; Federico A; Masarone M; Persico M. Pharmacotherapy of alcoholic liver disease in clinical practice. 2016. DOI: 10.1111/ijcp.12764
209. Wilson, David B.. Meta-analyses in alcohol and other drug abuse treatment research. 2000. DOI: 10.1080/09652140020004313
210. Maisel, N.C.; Blodgett, J.C.; Wilbourne, P.L.; Humphreys, K.; Finney, J.W.. Meta-analysis of naltrexone and acamprosate for treating alcohol dependence: When are these medications most helpful? 2012. DOI: 10.1111/j.1530-0277.2012.01803.x
211. Tong HL; Quiroz JC; Kocaballi AB; Fat SCM; Dao KP; Gehringer H; Chow CK; Laranjo L. Personalized mobile technologies for lifestyle behavior change: A systematic review, meta-analysis, and meta-regression. 2021. DOI: 10.1016/j.ypmed.2021.106532
212. Schmidt, L.; Nielsen, A.S.; Bojesen, A.B.; Andersen, K.. Research assessments more important than duration of treatment? A systematic review and meta-analysis of the duration of psychosocial treatments for alcohol use disorders 2016. DOI: 10.1016/j.eurpsy.2016.01.133
213. Tonigan JS; Toscova R; Miller WR. Meta-analysis of the literature on Alcoholics Anonymous: sample and study characteristics moderate findings. 1996. DOI: 10.15288/jsa.1996.57.65
214. Muzyk AJ; Leung JG; Nelson S; Embury ER; Jones SR. The role of diazepam loading for the treatment of alcohol withdrawal syndrome in hospitalized patients. 2013. DOI: 10.1111/j.1521-0391.2013.00307.x
215. Stockings E; Hall WD; Lynskey M; Morley KI; Reavley N; Strang J; Patton G; Degenhardt L. Prevention, early intervention, harm reduction, and treatment of substance use in young people. 2016. DOI: 10.1016/S2215-0366(16)00002-X
216. Ungur LA; Neuner B; John S; Wernecke K; Spies C. Prevention and therapy of alcohol withdrawal on intensive care units: systematic review of controlled trials. 2013. DOI: 10.1111/acer.12002
217. Litten RZ; Castle IJ; Falk D; Ryan M; Fertig J; Chen CM; Yi HY. The placebo effect in clinical trials for alcohol dependence: an exploratory analysis of 51 naltrexone and acamprosate studies. 2013. DOI: 10.1111/acer.12197
218. Rowe, J.; Hammond, D.; Wiley, T.; Lee, K.; Kane-Gill, S.. Patient outcomes for phenobarbital use with or without benzodiazepines in alcohol withdrawal syndrome: Systematic review 2016. DOI: 10.1002/phar.1877
219. Vipond J; Mennenga HA. Screening, Brief Intervention, and Referral to Treatment by Emergency Nurses: A Review of the Literature. 2019. DOI: 10.1016/j.jen.2018.10.004
220. Werch, C.E.; Owen, D.M.. Iatrogenic effects of alcohol and drug prevention programs 2002. DOI: 10.15288/jsa.2002.63.581
221. Wright NM; Tompkins CN. How can health services effectively meet the health needs of homeless people? 2006. DOI:
222. Scott-Sheldon LA; Carey KB; Elliott JC; Garey L; Carey MP. Efficacy of alcohol interventions for first-year college students: a meta-analytic review of randomized controlled trials. 2014. DOI: 10.1037/a0035192
223. Poikolainen K. Effectiveness of brief interventions to reduce alcohol intake in primary health care populations: a meta-analysis. 1999. DOI: 10.1006/pmed.1999.0467
224. Luty, J.. Drug and alcohol addiction: New pharmacotherapies 2015. DOI: 10.1192/apt.bp.114.013367
225. Yuma-Guerrero PJ; Lawson KA; Velasquez MM; von Sternberg K; Maxson T; Garcia N. Screening, brief intervention, and referral for alcohol use in adolescents: a systematic review. 2012. DOI: 10.1542/peds.2011-1589
226. Roerecke M; Sorensen P; Laramée P; Rahhali N; Rehm J. Clinical relevance of nalmefene versus placebo in alcohol treatment: reduction in mortality risk. 2015. DOI: 10.1177/0269881115602487
227. Mason BJ; Quello S; Shadan F. Gabapentin for the treatment of alcohol use disorder. 2018. DOI: 10.1080/13543784.2018.1417383
228. Spijker, A.T.; Vanzaane, J.; Koenders, M.A.; Hoekstra, R.; Kupka, R.W.. Bipolar disorder and alcohol use disorder: Practical recommendations for treatment, based on a literature review 2018. DOI:
229. Tracy K; Wallace SP. Benefits of peer support groups in the treatment of addiction. 2016. DOI: 10.2147/SAR.S81535
230. Dos Santos RG; Osório FL; Crippa JA; Riba J; Zuardi AW; Hallak JE. Antidepressive, anxiolytic, and antiaddictive effects of ayahuasca, psilocybin and lysergic acid diethylamide (LSD): a systematic review of clinical trials published in the last 25 years. 2016. DOI: 10.1177/2045125316638008
231. García-Torres, F.; Alós, F.J.; Castillo-Mayén, R.. Alcohol consumption in cancer survivors: State of the question and proposals for the development of psychological interventions 2018. DOI: 10.5209/PSIC.59175
232. Walton-Moss B; Ray EM; Woodruff K. Relationship of spirituality or religion to recovery from substance abuse: a systematic review. 2013. DOI: 10.1097/JAN.0000000000000001
233. Thoele K; Moffat L; Konicek S; Lam-Chi M; Newkirk E; Fulton J; Newhouse R. Strategies to promote the implementation of Screening, Brief Intervention, and Referral to Treatment (SBIRT) in healthcare settings: a scoping review. 2021. DOI: 10.1186/s13011-021-00380-z
234. Segawa T; Baudry T; Bourla A; Blanc JV; Peretti CS; Mouchabac S; Ferreri F. Virtual Reality (VR) in Assessment and Treatment of Addictive Disorders: A Systematic Review. 2019. DOI: 10.3389/fnins.2019.01409
235. Maatoug R; Bihan K; Duriez P; Podevin P; Silveira-Reis-Brito L; Benyamina A; Valero-Cabré A; Millet B. Non-invasive and invasive brain stimulation in alcohol use disorders: A critical review of selected human evidence and methodological considerations to guide future research. 2021. DOI: 10.1016/j.comppsych.2021.152257
236. Kownacki RJ; Shadish WR. Does Alcoholics Anonymous work? The results from a meta-analysis of controlled experiments. 1999. DOI: 10.3109/10826089909039431
237. Guglielmo R; Martinotti G; Quatrale M; Ioime L; Kadilli I; Di Nicola M; Janiri L. Topiramate in Alcohol Use Disorders: Review and Update. 2015. DOI: 10.1007/s40263-015-0244-0
238. D'Onofrio G; Degutis LC. Preventive care in the emergency department: screening and brief intervention for alcohol problems in the emergency department: a systematic review. 2002. DOI: 10.1111/j.1553-2712.2002.tb02304.x
239. Stautz K; Zupan Z; Field M; Marteau TM. Does self-control modify the impact of interventions to change alcohol, tobacco, and food consumption? A systematic review. 2018. DOI: 10.1080/17437199.2017.1421477
240. Tebb KP; Erenrich RK; Jasik CB; Berna MS; Lester JC; Ozer EM. Use of theory in computer-based interventions to reduce alcohol use among adolescents and young adults: a systematic review. 2016. DOI: 10.1186/s12889-016-3183-x
241. Andersen KAA; Carhart-Harris R; Nutt DJ; Erritzoe D. Therapeutic effects of classic serotonergic psychedelics: A systematic review of modern-era clinical studies. 2021. DOI: 10.1111/acps.13249
242. O'Connor E; Thomas R; Senger CA; Perdue L; Robalino S; Patnode C. Interventions to Prevent Illicit and Nonmedical Drug Use in Children, Adolescents, and Young Adults: Updated Evidence Report and Systematic Review for the US Preventive Services Task Force. 2020. DOI: 10.1001/jama.2020.1432
243. Jonas DE; Amick HR; Feltner C; Bobashev G; Thomas K; Wines R; Kim MM; Shanahan E; Gass CE; Rowe CJ; Garbutt JC. Pharmacotherapy for adults with alcohol use disorders in outpatient settings: a systematic review and meta-analysis. 2014. DOI: 10.1001/jama.2014.3628
244. Sawicka M; Tracy DK. Naltrexone efficacy in treating alcohol-use disorder in individuals with comorbid psychosis: a systematic review. 2017. DOI: 10.1177/2045125317709975
245. Hartung DM; McCarty D; Fu R; Wiest K; Chalk M; Gastfriend DR. Extended-release naltrexone for alcohol and opioid dependence: a meta-analysis of healthcare utilization studies. 2014. DOI: 10.1016/j.jsat.2014.03.007
246. Lock, C.A.. Screening and brief alcohol interventions: What, why, who, where and when? A review of the literature 2004. DOI: 10.1080/14659890410001665096
247. Hennessy EA; Tanner-Smith EE. Effectiveness of brief school-based interventions for adolescents: a meta-analysis of alcohol use prevention programs. 2015. DOI: 10.1007/s11121-014-0512-0
248. Magwood O; Salvalaggio G; Beder M; Kendall C; Kpade V; Daghmach W; Habonimana G; Marshall Z; Snyder E; O'Shea T; Lennox R; Hsu H; Tugwell P; Pottie K. The effectiveness of substance use interventions for homeless and vulnerably housed persons:  A systematic review of systematic reviews on supervised consumption facilities, managed alcohol programs, and pharmacological agents for opioid use disorder. 2020. DOI: 10.1371/journal.pone.0227298
249. Corrigan JD; Bogner J; Hungerford DW; Schomer K. Screening and brief intervention for substance misuse among patients with traumatic brain injury. 2010. DOI: 10.1097/TA.0b013e3181e904cc
250. Mujoomdar M; Spry C. Naltrexone for the treatment of alcohol dependence: a review of the clinical and cost-effectiveness 2009. DOI:
251. Del Re AC; Maisel N; Blodgett J; Finney J. The declining efficacy of naltrexone pharmacotherapy for alcohol use disorders over time: a multivariate meta-analysis. 2013. DOI: 10.1111/acer.12067
252. Song S; Zilverstand A; Gui W; Li HJ; Zhou X. Effects of single-session versus multi-session non-invasive brain stimulation on craving and consumption in individuals with drug addiction, eating disorders or obesity: A meta-analysis. 2019. DOI: 10.1016/j.brs.2018.12.975
253. Rösner, S. Review: Acamprosate increases abstinence in patients with alcohol dependence 2011. DOI: 10.7326/0003-4819-154-2-201101180-02010
254. Yoo, E.R.; Cholankeril, G.; Ahmed, A.. Treating Alcohol Use Disorder in Chronic Liver Disease 2020. DOI: 10.1002/cld.881
255. Dalton K; Bishop L; Darcy S. Investigating interventions that lead to the highest treatment retention for emerging adults with substance use disorder: A systematic review. 2021. DOI: 10.1016/j.addbeh.2021.107005
256. Healey C; Rahman A; Faizal M; Kinderman P. Underage drinking in the UK: changing trends, impact and interventions. A rapid evidence synthesis. 2014. DOI: 10.1016/j.drugpo.2013.07.008
257. Agosti V; Nunes EV; O'Shea D. Do manualized psychosocial interventions help reduce relapse among alcohol-dependent adults treated with naltrexone or placebo? A meta-analysis. 2012. DOI: 10.1111/j.1521-0391.2012.00270.x
258. Luchsinger W; Zimbrean P. Systematic Review: Treatment for Addictive Disorder in Transplant Patients. 2020. DOI: 10.1111/ajad.13054
259. Leung, J.G.; Hall-Flavin, D.; Nelson, S.; Schmidt, K.A.; Schak, K.M.. Role of gabapentin in the management of alcohol withdrawal and dependence 2015. DOI: 10.1177/1060028015585849
260. . Comparing pharmacological interventions for alcohol-use disorders 2014. DOI: 10.1136/dtb.2014.8.0269
261. McMurran, M.. What works in substance misuse treatments for offenders? 2007. DOI: 10.1002/cbm.662
262. Burke BL; Arkowitz H; Menchola M. The efficacy of motivational interviewing: a meta-analysis of controlled clinical trials. 2003. DOI: 10.1037/0022-006X.71.5.843
263. Verheul R; Lehert P; Geerlings PJ; Koeter MW; van den Brink W. Predictors of acamprosate efficacy: results from a pooled analysis of seven European trials including 1485 alcohol-dependent patients. 2005. DOI: 10.1007/s00213-004-1991-7
264. Hoes, M.J.A.J.M.. Relapse prevention in alcoholics. A review of acamprosate versus naltrexone 1999. DOI: 10.2165/00044011-199917030-00005
265. Schulte B; O'Donnell AJ; Kastner S; Schmidt CS; Schäfer I; Reimer J. Alcohol screening and brief intervention in workplace settings and social services: a comparison of literature. 2014. DOI: 10.3389/fpsyt.2014.00131
266. Saitz R. Alcohol screening and brief intervention in primary care: Absence of evidence for efficacy in people with dependence or very heavy drinking. 2010. DOI: 10.1111/j.1465-3362.2010.00217.x
267. Patton R; Deluca P; Kaner E; Newbury-Birch D; Phillips T; Drummond C. Alcohol screening and brief intervention for adolescents: the how, what and where of reducing alcohol consumption and related harm among young people. 2014. DOI: 10.1093/alcalc/agt165
268. Simons-Morton, D.G.; Mullen, P.D.; Mains, D.A.; Tabak, E.R.; Green, L.W.. Characteristics of controlled studies of patient education and counseling for preventive health behaviors 1992. DOI: 10.1016/0738-3991(92)90196-P
269. Saitz, R.. What we know and don't know about the effectiveness of screening and brief interventions 2012. DOI: 10.1111/j.1530-0277.2012.01917.x
270. Naudet, F.. Pharmacologically controlled drinking in the treatment of alcohol use disorders (AUDs). A network meta-analysis 2018. DOI: 10.1111/fcp.12370
271. Hyman Z. Brief interventions for high-risk drinkers. 2006. DOI: 10.1111/j.1365-2702.2006.01458.x
272. Vale, A.. The management of alcohol withdrawal 2006. DOI: 10.1053/j.mpmed.2006.07.001
273. Hingson R; White A. New research findings since the 2007 Surgeon General's Call to Action to Prevent and Reduce Underage Drinking: a review. 2014. DOI: 10.15288/jsad.2014.75.158
274. Springer SA; Azar MM; Altice FL. HIV, alcohol dependence, and the criminal justice system: a review and call for evidence-based treatment for released prisoners. 2011. DOI: 10.3109/00952990.2010.540280
275. Powers MB; Vedel E; Emmelkamp PM. Behavioral couples therapy (BCT) for alcohol and drug use disorders: a meta-analysis. 2008. DOI: 10.1016/j.cpr.2008.02.002
276. Hendershot, C.S.; Wardell, J.D.; Samokhvalov, A.V.; Rehm, J.. Effects of naltrexone on alcohol self-administration and craving: Meta-analysis of human laboratory studies 2016. DOI: 10.1111/acer.13084
277. Pruckner, N.; Baumgartner, J.; Hinterbuchinger, B.; Vyssoki, B.. Medical guidelines for the use of thiamine in alcohol use disorder: Nolack of clinical efficacy, but lack of recommendations? 2018. DOI: 10.1111/acer.13833
278. Magill, M.; Kiluk, B.; Ray, L.A.; Walthers, J.; Bernstein, M.; Tonigan, J.S.; Carroll, K.. Technology-delivered cognitive-behavioral therapy for alcohol or other drug use disorders: Preliminary results from meta-analysis 2019. DOI: 10.1111/acer.14059
279. GaldurÃ³z JCF; Bezerra AG; Pires GN; Pauluci R; Noto AR. OMEGA-3 Interventions in Alcohol Dependence and Related Outcomes: A Systematic Review and Propositions. 2020. DOI: 10.2174/1570159X18666200128120729
280. Carey KB; Scott-Sheldon LA; Carey MP; DeMartini KS. Individual-level interventions to reduce college student drinking: a meta-analytic review. 2007. DOI: 10.1016/j.addbeh.2007.05.004
281. Wiers RW; Boffo M; Field M. What's in a Trial? On the Importance of Distinguishing Between Experimental Lab Studies and Randomized Controlled Trials: The Case of Cognitive Bias Modification and Alcohol Use Disorders. 2018. DOI:
282. Lehert, P.; VandenBrink, W.. Does acamprosate improve control of drinking as well as aiding abstinence? An individual patient data meta-analysis of 16 studies 2011. DOI: 10.1093/alcalc/agr123
283. Fuller, R.K.; Gordis, E.. Naltrexone treatment for alcohol dependence 2001. DOI: 10.1056/NEJM200112133452411
284. Coupet, E.; Dodington, J.; Brackett, A.; Vaca, F.E.. Emergency department screening, brief intervention, and referral to treatment of substance use in victims of community violence: A systematic review 2020. DOI: 10.1111/acem.13961
285. Carroll, Kathleen M.; Onken, Lisa S.. Behavioral Therapies for Drug Abuse. 2005. DOI: 10.1176/appi.ajp.162.8.1452
286. Moyer A; Finney JW; Swearingen CE; Vergun P. Brief interventions for alcohol problems: a meta-analytic review of controlled investigations in treatment-seeking and non-treatment-seeking populations. 2002. DOI: 10.1046/j.1360-0443.2002.00018.x
287. Gebara CF; Bhona FM; Ronzani TM; Lourenço LM; Noto AR. Brief intervention and decrease of alcohol consumption among women: a systematic review. 2013. DOI: 10.1186/1747-597X-8-31
288. Mun EY; de la Torre J; Atkins DC; White HR; Ray AE; Kim SY; Jiao Y; Clarke N; Huo Y; Larimer ME; Huh D. Project INTEGRATE: An integrative study of brief alcohol interventions for college students. 2015. DOI: 10.1037/adb0000047
289. Segura L; Anderson P; Gual A. Optimizing the delivery of interventions for harmful alcohol use in primary healthcare: an update. 2018. DOI: 10.1097/YCO.0000000000000435
290. Linn DD; Loeser KC. Dexmedetomidine for Alcohol Withdrawal Syndrome. 2015. DOI: 10.1177/1060028015607038
291. McGinnes, R.; Hutton, J.; Weiland, T.; Fatovich, D.; Egerton, D.. Emergency department based preventative health interventions targeting alcohol problems: A Systematic Review 2016. DOI: 10.1111/1742-6723.12614
292. French DP; Cameron E; Benton JS; Deaton C; Harvie M. Can Communicating Personalised Disease Risk Promote Healthy Behaviour Change? A Systematic Review of Systematic Reviews. 2017. DOI: 10.1007/s12160-017-9895-z
293. Fiellin DA; Reid MC; O'Connor PG. Screening for alcohol problems in primary care: a systematic review. 2000. DOI: 10.1001/archinte.160.13.1977
294. Walters, G.D.. Behavioral self-control training for problem drinkers: A meta-analysis of randomized control studies 2000. DOI: 10.1016/S0005-7894(00)80008-8
295. Garnett CV; Crane D; Brown J; Kaner EFS; Beyer FR; Muirhead CR; Hickman M; Beard E; Redmore J; de Vocht F; Michie S. Behavior Change Techniques Used in Digital Behavior Change Interventions to Reduce Excessive Alcohol Consumption: A Meta-regression. 2018. DOI: 10.1093/abm/kax029
296. Helton, S.G.; Lohoff, F.W.. Pharmacogenetics of alcohol use disorders and comorbid psychiatric disorders 2015. DOI: 10.1016/j.psychres.2015.09.019
297. Gold N; Yau A; Rigby B; Dyke C; Remfry EA; Chadborn T. Effectiveness of Digital Interventions for Reducing Behavioral Risks of Cardiovascular Disease in Nonclinical Adult Populations: Systematic Review of Reviews. 2021. DOI: 10.2196/19688
298. Field CA; Klimas J; Barry J; Bury G; Keenan E; Lyons S; Smyth BP; Cullen W. Alcohol screening and brief intervention among drug users in primary care: a discussion paper. 2012. DOI: 10.1007/s11845-011-0748-7
299. Reinholdz HK; Bendtsen P; Spak F. Different methods of early identification of risky drinking: a review of clinical signs. 2011. DOI: 10.1093/alcalc/agr021
300. Foxcroft DR; Lister-Sharp D; Lowe G. Alcohol misuse prevention for young people: a systematic review reveals methodological concerns and lack of reliable evidence of effectiveness. 1997. DOI:
301. Kranzler HR; Van Kirk J. Efficacy of naltrexone and acamprosate for alcoholism treatment: a meta-analysis. 2001. DOI:
302. Powers JM; Zvolensky MJ; Ditre JW. An integrative review of personalized feedback interventions for pain and alcohol. 2019. DOI: 10.1016/j.copsyc.2019.01.013
303. MacCormac A. Alcohol Dependence in Palliative Care: A Review of the Current Literature. 2017. DOI: 10.1177/0825859717738445
304. Batra A; MÃ¼ller CA; Mann K; Heinz A. Alcohol Dependence and Harmful Use of Alcohol. 2016. DOI: 10.3238/arztebl.2016.0301
305. Soyka M; Chick J. Use of acamprosate and opioid antagonists in the treatment of alcohol dependence: a European perspective. 2003. DOI: 10.1111/j.1521-0391.2003.tb00497.x
306. DeSantis SM; Zhu H. A Bayesian mixed-treatment comparison meta-analysis of treatments for alcohol dependence and implications for planning future trials. 2014. DOI: 10.1177/0272989X14537558
307. Kelly TM; Daley DC; Douaihy AB. Treatment of substance abusing patients with comorbid psychiatric disorders. 2012. DOI: 10.1016/j.addbeh.2011.09.010
308. Zisserson RN; Palfai T; Saitz R. 'No-contact' interventions for unhealthy college drinking:efficacy of alternatives to person-delivered intervention approaches. 2007. DOI: 10.1300/J465v28n04_04
309. Charlet K; Heinz A. Harm reduction-a systematic review on effects of alcohol reduction on physical and mental symptoms. 2017. DOI: 10.1111/adb.12414
310. Prochaska, J.J.; Burtner, J.L.; Delucchi, K.; Hall, S.M.. Tobacco treatment effects on long-term abstinence from alcohol and illicit drugs: A meta-analysis 2012. DOI: 10.1111/j.1530-0277.2012.01803.x
311. Lynch, M.-J.; George, T.P.. Therapeutic mechanisms underlying the effects of alcoholics anonymous: Results of a systematic literature review 2013. DOI: 10.1111/j.1521-0391.2013.12069.x
312. McCallum, S.; Mikocka-Walus, A.; Turnbull, D.; Gaughwin, M.; Andrews, J.. The role of continuity of care (COC) in the treatment of comorbid mental health and substance use disorders (SUDs): A systematic review of the literature 2013. DOI: 10.1016/j.jpsychores.2013.03.062
313. LÃ³pez-Pelayo H; Zuluaga P; Caballeria E; Van den Brink W; Mann K; Gual A. Safety of nalmefene for the treatment of alcohol use disorder: an update. 2020. DOI: 10.1080/14740338.2020.1707802
314. Labbe, A.K.; Maisto, S.A.. Alcohol expectancy challenges for college students: A narrative review 2011. DOI: 10.1016/j.cpr.2011.02.007
315. Kufahl, P.R.; Watterson, L.R.; Olive, M.F.. The development of acamprosate as a treatment against alcohol relapse 2014. DOI: 10.1517/17460441.2014.960840
316. Foxcroft DR; Tsertsvadze A. Universal alcohol misuse prevention programmes for children and adolescents: Cochrane systematic reviews. 2012. DOI: 10.1177/1757913912443487
317. Chick J. The efficacy of treatments in reducing alcohol consumption: a meta-analysis. 1996. DOI: 10.3109/10826089609063965
318. Hughes JC; Cook CC. The efficacy of disulfiram: a review of outcome studies. 1997.
319. Shorter GW; Heather N; Bray JW; Berman AH; Giles EL; O'Donnell AJ; Barbosa C; Clarke M; Holloway A; Newbury-Birch D. Prioritization of Outcomes in Efficacy and Effectiveness of Alcohol Brief Intervention Trials: International Multi-Stakeholder e-Delphi Consensus Study to Inform a Core Outcome Set. 2019.
320. Walczak, J.; Jarosz, J.; Miernik, K.; Wchal, M.; Krumpl, G. Naltrexone significantly reduces percentage of drinking days, number of drinks per drinking days and alcohol craving-results of the meta-analysis 2013. DOI: 10.1093/alcalc/agt114
321. Guirguis, E.; Richardson, J.; Kuhn, T.; Fahmy, A. Treatment of Severe Alcohol Withdrawal: A Focus on Adjunctive Agents 2017. DOI: 10.1177/8755122517714491
322. Feinn R; Kranzler HR. Does effect size in naltrexone trials for alcohol dependence differ for single-site vs. multi-center studies? 2005. DOI: 10.1097/01.alc.0000171061.03686.bc
323. Magill, M.; Gaume, J.; Apodaca, T.R.; Walthers, J.; Mastroleo, N.R.; Borsari, B.; Longabaugh, R.. The technical hypothesis of motivational interviewing: A meta-analysis of mi's key causal model 2013. DOI: 10.1111/acer.12162
324. Malec TS; Malec EA; Dongier M. Efficacy of buspirone in alcohol dependence: a review. 1996. DOI: 10.1111/j.1530-0277.1996.tb05263.x
325. Weinrieb, R.M.; O'Brien, C.P. Naltrexone in the treatment of alcoholism 1997. DOI: 10.1146/annurev.med.48.1.477
326. Frost H; Campbell P; Maxwell M; O'Carroll RE; Dombrowski SU; Williams B; Cheyne H; Coles E; Pollock A. Effectiveness of Motivational Interviewing on adult behaviour change in health and social care settings: A systematic review of reviews. 2018. DOI: 10.1371/journal.pone.0204890
327. Williams D; McBride AJ. The drug treatment of alcohol withdrawal symptoms: a systematic review. 1998. DOI: 10.1093/oxfordjournals.alcalc.a008365
328. Hillemacher T; Frieling H. Pharmacotherapeutic options for co-morbid depression and alcohol dependence. 2019. DOI: 10.1080/14656566.2018.1561870
329. Kerr S; Lawrence M; Darbyshire C; Middleton AR; Fitzsimmons L. Tobacco and alcohol-related interventions for people with mild/moderate intellectual disabilities: a systematic review of the literature. 2013. DOI: 10.1111/j.1365-2788.2012.01543.x
330. Altinbas, K.; Evren, C.. Pharmacotherapy options in comorbid bipolar disorder and alcohol-substance use disorders 2013. DOI: 10.5455/bcp.20131122111840
331. Martin K; Katz A. The Role of Barbiturates for Alcohol Withdrawal Syndrome. 2016. DOI: 10.1016/j.psym.2016.02.011
332. Lundahl B; Burke BL. The effectiveness and applicability of motivational interviewing: a practice-friendly review of four meta-analyses. 2009. DOI: 10.1002/jclp.20638
333. Grodin EN; Ray LA. The Use of Functional Magnetic Resonance Imaging to Test Pharmacotherapies for Alcohol Use Disorder: A Systematic Review. 2019. DOI: 10.1111/acer.14167
334. Henderson, C.E.; Weinstock, J.. Integrative data analysis of exercise-based interventions for alcohol use 2016. DOI: 10.1111/acer.13085
335. Ray, L.; Du, H.; Green, R.; Roche, D.; Bujarski, S.. Do behavioral pharmacology findings predict clinical trials outcomes? A proof-of-concept in medication development for alcohol use disorder 2020. DOI: 10.1038/s41386-020-00890-7
336. Tampi, R.; Chhatlani, A.; Ahmad, H.; Balaram, K.; Dey, J.; Escobar, R.; Lingamchetty, T.. PHARMACOTHERAPY FOR SUBSTANCE USE DISORDERS AMONG OLDER ADULTS: A SYSTEMATIC REVIEW OF RANDOMIZED CONTROLLED TRIALS 2019. DOI: 10.1016/j.jagp.2019.01.072
337. Garzola, G.C.Q.. The use of gabapentin for the treatment of alcohol and tobacco use disorders: A review 2020. DOI: 10.2147/OAJCT.S257556
338. Kirchoff, R.W.; Mohammed, N.M.; McHugh, J.; Markota, M.; Kingsley, T.; Leung, J.; Burton, M.C.; Chaudhary, R.. Naltrexone Initiation in the Inpatient Setting for Alcohol Use Disorder: A Systematic Review of Clinical Outcomes 2021. DOI: 10.1016/j.mayocpiqo.2021.01.013
339. Jones LA. Systematic review of alcohol screening tools for use in the emergency department. 2011. DOI: 10.1136/emj.2009.085324
340. Brown ES. Management of comorbid bipolar disorder and substance abuse. 2006. DOI: 10.4088/jcp.0806e05
341. Berner, M.; Gänzler, C.; Frick, K.; Kriston, L.; Loessl, B.; Bräck, R.; Gann, H.; Batra, A.; Mann, K. Finding the ideal place for a psychotherapeutic intervention in a stepped care approach - A brief overview of the literature and preliminary results from the Project PREDICT 2008. DOI: 10.1002/mpr.250
342. Rivest J; Jutras-Aswad D; Shapiro PA. Treating the "unhealthy alcohol user" on medical wards: beyond withdrawal. 2013. DOI: 10.1097/01.pra.0000430505.52391.48
343. Cropsey, K.L.; Villalobos, G.C.; St. Clair, C.L.. Pharmacotherapy treatment in substance-dependent correctional populations: A review 2005. DOI: 10.1080/10826080500294866
344. Alharbi, F.; El-Guebaly, N.. Disulfiram: The survivor medication 2012. DOI: 10.1097/ADT.0b013e318246f4af
345. Nigg CR; Long CR. A systematic review of single health behavior change interventions vs. multiple health behavior change interventions among older adults. 2012. DOI: 10.1007/s13142-012-0130-y
346. Martens MP; Dams-O'Connor K; Beck NC. A systematic review of college student-athlete drinking: Prevalence rates, sport-related factors, and interventions. 2006. DOI: 10.1016/j.jsat.2006.05.004
347. Addolorato, G.; Vassallo, G.A.; Mirijello, A.; Gasbarrini, A.. Diagnosis and Management of Alcohol Use Disorder in Patients with Liver Disease: Lights and Shadows 2020. DOI: 10.1007/s13311-019-00802-8
348. Hesse M; Vanderplasschen W; Rapp RC; Broekaert E; Fridell M. Case management for persons with substance use disorders. 2007. DOI: 10.1002/14651858.CD006265.pub2
349. NHS Centre for Reviews and Dissemination. Brief interventions and alcohol use 1993. DOI:
350. Doyle, J.S.; Hunt, D.; Aspinall, E.J.; Hutchinson, S.J.; Goldberg, D.J.; Nguyen, T.; Falck-Ytter, Y.; Morgan, R.L.; Smith, B.; Stoove, M.; Lutchers, S.; Thompson, A.J.; Wiktor, S.Z.; Hellard, M.E.. A systematic review of interventions to reduce alcohol consumption among individuals with chronic HCV infection 2014. DOI: 10.1016/S0168-8278(14)60895-3
351. Coles AS; Kozak K; George TP. A review of brain stimulation methods to treat substance use disorders. 2018. DOI: 10.1111/ajad.12674
352. Hartwell, E.E.; Feinn, R.; Morris, P.; Kranzler, H.R.. A meta-analysis of the efficacy of gabapentin for treating alcohol use disorder 2019. DOI: 10.1111/acer.14059
353. Berglund M; Andreasson S; Franck J; Fridell M; Hakanson I; Johansson B-A et al. Treatment of alcohol and drug abuse - an evidence-based review 2001. DOI:
354. Dos Santos RG; Bouso JC; Alcázar-Córcoles MÁ; Hallak JEC. Efficacy, tolerability, and safety of serotonergic psychedelics for the management of mood, anxiety, and substance-use disorders: a systematic review of systematic reviews. 2018. DOI: 10.1080/17512433.2018.1511424
355. Chiesa, A.; Serretti, A.. A systematic review of neurobiological and clinical features of mindfulness meditations 2010. DOI: 10.1016/S0924-9338(10)71034-8
356. Ickes, M.J.; Haider, T.; Sharma, M.. Alcohol abuse prevention programs in college students 2015. DOI: 10.3109/14659891.2013.856480
357. Rongione, Danielle; Erford, Bradley T.; Broglie, Caren. Alcohol and other drug abuse counseling outcomes for school-aged youth: A meta-analysis of studies from 1990 to 2009. 2011. DOI: 10.1177/2150137811400595
358. Hanstock, T.; Speirs, B.; Kay-Lambkin, F.. A large systematic review of evidence for lifestyle interventions targeting smoking, sleep, alcohol/other drug use, physic al activity and healthy diet in people with bipolar disorder 2021. DOI: 10.1111/bdi.13087
359. Kenna GA; Lomastro TL; Schiesl A; Leggio L; Swift RM. Review of topiramate: an antiepileptic for the treatment of alcohol dependence. 2009. DOI: 10.2174/1874473710902020135
360. Murphy JA; Curran BM; Gibbons WA 3rd; Harnica HM. Adjunctive Phenobarbital for Alcohol Withdrawal Syndrome: A Focused Literature Review. 2021. DOI: 10.1177/1060028021999821
361. Rosenthal, R.N.; Brady, K.T.; Levounis, P.; Willenbring, M.L.. Advances in the treatment of alcohol dependence 2007. DOI: 10.4088/jcp.v68n0721
362. Manuel JK; Satre DD; Tsoh J; Moreno-John G; Ramos JS; McCance-Katz EF; Satterfield JM. Adapting Screening, Brief Intervention, and Referral to Treatment for Alcohol and Drugs to Culturally Diverse Clinical Populations. 2015. DOI: 10.1097/ADM.0000000000000150
363. Whitlock EP; Green CA; Polen MR; Berg A; Klein J; Siu A; Orleans CT. 2004. DOI:
364. Jonas DE; Garbutt JC; Brown JM; Amick HR; Brownley KA; Council CL; Viera AJ; Wilkins TM; Schwartz CJ; Richmond EM; Yeatts J; Swinson Evans T; Wood SD; Harris RP. 2012. DOI:
365. Chun TH; Linakis JG. Interventions for adolescent alcohol use. 2012. DOI: 10.1097/MOP.0b013e32834faa83
366. Graham, R.; Wodak, A.D.; Whelan, G.. New pharmacotherapies for alcohol dependence 2002. DOI: 10.5694/j.1326-5377.2002.tb04683.x
367. Kelly, S.; Olanrewaju, O.; Cowan, A.; Brayne, C.; Lafortune, L.. Alcohol in older people: Systematic reviews of interventions and context 2016. DOI:
368. Jones A; Di Lemma LC; Robinson E; Christiansen P; Nolan S; Tudur-Smith C; Field M. Inhibitory control training for appetitive behaviour change: A meta-analytic investigation of mechanisms of action and moderators of effectiveness. 2016. DOI: 10.1016/j.appet.2015.11.013
369. Muller M. Commentary: use of meta-regression in the review on brief alcohol interventions in primary care populations. 2007. DOI:
370. Jones, L.; Lucas, B.; Plampin, P.; Wingerson, C.; Cash, J.; Shealy, K.. Adjuvant therapies in severe benzodiazepine-refractory alcohol withdrawal syndrome: A systematic review 2020. DOI: 10.1002/jac5.1351
371. Cashman CM; Ruotsalainen JH; Greiner BA; Beirne PV; Verbeek JH. Alcohol and drug screening of occupational drivers for preventing injury. 2009. DOI: 10.1002/14651858.CD006566.pub2
372. Poikolainen K. A nice try that fails: the Swedish Council on Technology Assessment in Health Care (SBU) evaluation of the effect of treatment of alcohol and drug problems: the epidemiologist's view. 2002. DOI: 10.1093/alcalc/37.5.416
373. Salwan, J.; Katz, C.L.. A review of substance use disorder treatment in developing world communities 2014. DOI: 10.1016/j.aogh.2014.04.010
374. Poikolainen, K.. A nice try that fails: The Swedish council on technology assessment in health care (SBU) evaluation of the effect of treatment of alcohol and drugs problems: The epidemiologist's view 2002. DOI:
375. Ritter, A.; Cameron, J.. A review of the efficacy and effectiveness of harm reduction strategies for alcohol, tobacco and illicit drugs 2006. DOI: 10.1080/09595230600944529
376. Álvarez-Bueno C; Rodríguez-Martín B; García-Ortiz L; Gómez-Marcos MÃ; Martínez-Vizcaíno V. Effectiveness of brief interventions in primary health care settings to decrease alcohol consumption by adult non-dependent drinkers: a systematic review of systematic reviews. 2015. DOI: 10.1016/j.ypmed.2014.12.010
377. Lui S; Terplan M; Smith EJ. Psychosocial interventions for women enrolled in alcohol treatment during pregnancy. 2008. DOI: 10.1002/14651858.CD006753.pub2

**Exclusion reason 4:** No meta-analysis (or only one RCT)

1. Smith EJ; Lui S; Terplan M. Pharmacologic interventions for pregnant women enrolled in alcohol treatment. 2009. DOI: 10.1002/14651858.CD007361.pub2
2. Acin MT; Rueda JR; Saiz LC; Parent Mathias V; Alzueta N; Sola; Garjón J; Erviti J Alcohol intake reduction for controlling hypertension.2020. DOI: 10.1002/14651858.CD010022.pub2
3. Ferri M; Amato L; Davoli M Alcoholics Anonymous and other 12-step programmes for alcohol dependence.2006. DOI: 10.1002/14651858.CD005032.pub2
4. Stade BC; Bailey C; Dzendoletas D; Sgro M; Dowswell T; Bennett D Psychological and/or educational interventions for reducing alcohol consumption in pregnant women and women planning pregnancy.2009. DOI: 10.1002/14651858.CD004228.pub2
5. Martinotti G; Orsolini L; Fornaro M; Vecchiotti R; De Berardis D; Iasevoli F; Torrens M; Di Giannantonio M Aripiprazole for relapse prevention and craving in alcohol use disorder: current evidence and future perspectives.2016. DOI: 10.1080/13543784.2016.1175431
6. Sims OT; Maynard QR; Melton PA Behavioral Interventions to Reduce Alcohol Use Among Patients with Hepatitis C: A Systematic Review.2016. DOI: 10.1080/19371918.2016.1160346
7. Mo Y; Thomas MC; Karras GE Jr Barbiturates for the treatment of alcohol withdrawal syndrome: A systematic review of clinical trials.2016. DOI: 10.1016/j.jcrc.2015.11.022
8. Cubillos, L.; Bartels, S.M.; Torrey, W.C.; Naslund, J.; Uribe-Restrepo, J.M.; Gaviola, C.; DÃ­az, S.C.; John, D.T.; Williams, M.J.; Cepeda, M.; GÃ³mez-Restrepo, C.; Marsch, L.A. The effectiveness and cost-effectiveness of integrating mental health services in primary care in low-and middle-income countries: Systematic review2021. DOI: 10.1192/bjb.2020.35
9. Petrie J; Bunn F; Byrne G Parenting programmes for preventing tobacco, alcohol or drugs misuse in children <18: a systematic review.2007. DOI: 10.1093/her/cyl061
10. Colbert S; Thornton L; Richmond R Smartphone apps for managing alcohol consumption: a literature review.2020. DOI: 10.1186/s13722-020-00190-x
11. Doyle MF; Shakeshaft A; Guthrie J; Snijder M; Butler T A systematic review of evaluations of prison-based alcohol and other drug use behavioural treatment for men.2019. DOI: 10.1111/1753-6405.12884
12. Fiestas F; Ponce J [Efficacy of the therapeutic community model in the treatment of drug use-related problems: a systematic review].2012. DOI: 10.1590/s1726-46342012000100003
13. Vogelgesang M [DGRW update: alcohol addiction].2011. DOI: 10.1055/s-0031-1285892
14. Phillips EA; Gordeev VS; Schreyögg J Effectiveness of occupational e-mental health interventions: a systematic review and meta-analysis of randomized controlled trials.2019. DOI: 10.5271/sjweh.3839
15. Körkel, J. Controlled drinking as a treatment goal for at-risk drinking and alcohol use disorders: A systematic review2015. DOI: 10.1024/0939-5911.a000367
16. Plotnikoff RC; Costigan SA; Kennedy SG; Robards SL; Germov J; Wild C Efficacy of interventions targeting alcohol, drug and smoking behaviors in university and college students: A review of randomized controlled trials.2019. DOI: 10.1080/07448481.2018.1462821
17. Kruse CS; Lee K; Watson JB; Lobo LG; Stoppelmoor AG; Oyibo SE Measures of Effectiveness, Efficiency, and Quality of Telemedicine in the Management of Alcohol Abuse, Addiction, and Rehabilitation: Systematic Review.2020. DOI: 10.2196/13252
18. Bouza Alvarez C; Magro de la Plaza M A; Romero Martinez J J; Amate Blanco J M Assessment of therapeutic strategies for alcohol dependence: opioid antagonists and acamprosate. IPE-02/35 (Public report)2002. DOI:
19. Petrakis IL; Simpson TL Posttraumatic Stress Disorder and Alcohol Use Disorder: AÂ Critical Review of Pharmacologic Treatments.2017. DOI: 10.1111/acer.13297
20. Appiah-Brempong E; Okyere P; Owusu-Addo E; Cross R Motivational interviewing interventions and alcohol abuse among college students: a systematic review.2014. DOI: 10.4278/ajhp.130502-LIT-222
21. Magill M; Tonigan JS; Kiluk B; Ray L; Walthers J; Carroll K The search for mechanisms of cognitive behavioral therapy for alcohol or other drug use disorders: A systematic review.2020. DOI: 10.1016/j.brat.2020.103648
22. Milne-Ives M; Lam C; De Cock C; Van Velthoven MH; Meinert E Mobile Apps for Health Behavior Change in Physical Activity, Diet, Drug and Alcohol Use, and Mental Health: Systematic Review.2020. DOI: 10.2196/17046
23. Simpson TL; Lehavot K; Petrakis IL No Wrong Doors: Findings from a Critical Review of Behavioral Randomized Clinical Trials for Individuals with Co-Occurring Alcohol/Drug Problems and Posttraumatic Stress Disorder.2017. DOI: 10.1111/acer.13325
24. Lefio LÁ; Villarroel SR; Rebolledo C; Zamorano P; Rivas K [Effective interventions in the problematic use of alcohol and other drugs].2013. DOI:
25. Maher CA; Lewis LK; Ferrar K; Marshall S; De Bourdeaudhuij I; Vandelanotte C Are health behavior change interventions that use online social networks effective? A systematic review.2014. DOI: 10.2196/jmir.2952
26. Roozen HG; de Waart R; van der Kroft P Community reinforcement and family training: an effective option to engage treatment-resistant substance-abusing individuals in treatment.2010. DOI: 10.1111/j.1360-0443.2010.03016.x
27. Watson JM; Fayter D; Mdege N; Stirk L; Sowden AJ; Godfrey C Interventions for alcohol and drug problems in outpatient settings: a systematic review.2013. DOI: 10.1111/dar.12037
28. Foxcroft DR; Ireland D; Lister-Sharp DJ; Lowe G; Breen R Longer-term primary prevention for alcohol misuse in young people: a systematic review.2003. DOI: 10.1046/j.1360-0443.2003.00355.x
29. Hou SI; Charlery SA; Roberson K Systematic literature review of Internet interventions across health behaviors.2014. DOI: 10.1080/21642850.2014.895368
30. Morton K; Beauchamp M; Prothero A; Joyce L; Saunders L; Spencer-Bowdage S; Dancy B; Pedlar C The effectiveness of motivational interviewing for health behaviour change in primary care settings: a systematic review.2015. DOI: 10.1080/17437199.2014.882006
31. Humphreys G; Evans R; Makin H; Cooke R; Jones A Identification of Behavior Change Techniques From Successful Web-Based Interventions Targeting Alcohol Consumption, Binge Eating, and Gambling: Systematic Review.2021. DOI: 10.2196/22694
32. Chiesa A; Serretti A Are mindfulness-based interventions effective for substance use disorders? A systematic review of the evidence.2014. DOI: 10.3109/10826084.2013.770027
33. Coles AS; Sasiadek J; George TP Pharmacotherapies for co-occurring substance use and bipolar disorders: A systematic review.2019. DOI: 10.1111/bdi.12794
34. Azorin JM; Bowden CL; Garay RP; Perugi G; Vieta E; Young AH Possible new ways in the pharmacological treatment of bipolar disorder and comorbid alcoholism.2010. DOI: 10.2147/ndt.s6741
35. Lobmaier PP; KunÃ¸e N; Gossop M; Waal H Naltrexone depot formulations for opioid and alcohol dependence: a systematic review.2011. DOI: 10.1111/j.1755-5949.2010.00194.x
36. Hafford-Letchfield T; McQuarrie T; Clancy C; Thom B; Jain B Community Based Interventions for Problematic Substance Use in Later Life: A Systematic Review of Evaluated Studies and Their Outcomes.2020. DOI: 10.3390/ijerph17217994
37. Byrne SP; Haber P; Baillie A; Costa DSJ; Fogliati V; Morley K Systematic Reviews of Mindfulness and Acceptance and Commitment Therapy for Alcohol Use Disorder: Should we be using Third Wave Therapies?2019. DOI: 10.1093/alcalc/agy089
38. Maiti R; Mishra BR; Hota D Effect of High-Frequency Transcranial Magnetic Stimulation on Craving in Substance Use Disorder: A Meta-Analysis.2017. DOI: 10.1176/appi.neuropsych.16040065
39. Thibault A; Brissette S; Jutras-Aswad D Systematic review of the pharmacological treatment of alcohol use disorders in individuals infected with hepatitis C.2015. DOI: 10.1186/s13722-015-0029-2
40. Kedzior KK; Gerkensmeier I; Schuchinsky M Can deep transcranial magnetic stimulation (DTMS) be used to treat substance use disorders (SUD)? A systematic review.2018. DOI: 10.1186/s12888-018-1704-0
41. Garcia-Huidobro D; Doty JL; Davis L; Borowsky IW; Allen ML For Whom Do Parenting Interventions to Prevent Adolescent Substance Use Work?2018. DOI: 10.1007/s11121-017-0853-6
42. Giroux I; Goulet A; Mercier J; Jacques C; Bouchard S Online and Mobile Interventions for Problem Gambling, Alcohol, and Drugs: A Systematic Review.2017. DOI: 10.3389/fpsyg.2017.00954
43. Champion, K.E.; Newton, N.C.; Barrett, E.L.; Teesson, M. A systematic review of school-based alcohol and other drug prevention programs facilitated by computers or the Internet2013. DOI: 10.1111/j.1465-3362.2012.00517.x
44. Canidate SS; Carnaby GD; Cook CL; Cook RL A Systematic Review of Naltrexone for Attenuating Alcohol Consumption in Women with Alcohol Use Disorders.2017. DOI: 10.1111/acer.13313
45. Hunt GE; Siegfried N; Morley K; Brooke-Sumner C; Cleary M Psychosocial interventions for people with both severe mental illness and substance misuse.2019. DOI: 10.1002/14651858.CD001088.pub4
46. Carreiro S; Newcomb M; Leach R; Ostrowski S; Boudreaux ED; Amante D Current reporting of usability and impact of mHealth interventions for substance use disorder: A systematic review.2020. DOI: 10.1016/j.drugalcdep.2020.108201
47. Danielsson AK; Eriksson AK; Allebeck P Technology-based support via telephone or web: a systematic review of the effects on smoking, alcohol use and gambling.2014. DOI: 10.1016/j.addbeh.2014.06.007
48. Taggart IH; Ranney ML; Howland J; Mello MJ A systematic review of emergency department interventions for college drinkers.2013. DOI: 10.1016/j.jemermed.2013.05.065
49. Bonnet U; Schäfer M; Richter C; Milkereit J; Wiltfang J; Scherbaum N; Lieb B [Anticonvulsants in the treatment of alcoholism].2009. DOI: 10.1055/s-0028-1109214
50. Koeter MW; van den Brink W; Lehert P Effect of early and late compliance on the effectiveness of acamprosate in the treatment of alcohol dependence.2010. DOI: 10.1016/j.jsat.2010.06.002
51. Wang, K.; Varma, D.S.; Prosperi, M. A systematic review of the effectiveness of mobile apps for monitoring and management of mental health symptoms or disorders2018. DOI: 10.1016/j.jpsychires.2018.10.006
52. Barata IA; Shandro JR; Montgomery M; Polansky R; Sachs CJ; Duber HC; Weaver LM; Heins A; Owen HS; Josephson EB; Macias-Konstantopoulos W Effectiveness of SBIRT for Alcohol Use Disorders in the Emergency Department: A Systematic Review.2017. DOI: 10.5811/westjem.2017.7.34373
53. Laging, M. Web-based interventions for addressing college drinking2012. DOI: 10.1024/0939-5911.a000168
54. Bharadwaj B; Selvakumar N; Kuppili PP Pharmacotherapy for relapse prevention of alcohol use disorder in the Indian setting: A systematic review.2018. DOI: 10.4103/ipj.ipj_79_17
55. Boothby LA; Doering PL Acamprosate for the treatment of alcohol dependence.2005. DOI: 10.1016/j.clinthera.2005.06.015
56. Penzenstadler, L.; Soares, C.; Anci, E.; Molodynski, A.; Khazaal, Y. Effect of Assertive Community Treatment for Patients with Substance Use Disorder: A Systematic Review2019. DOI: 10.1159/000496742
57. Durl, J.; Dietrich, T.; Pang, B.; Potter, L.-E.; Carter, L. Utilising virtual reality in alcohol studies: A systematic review2018. DOI: 10.1177/0017896917743534
58. Ahmed S; Bachu R; Kotapati P; Adnan M; Ahmed R; Farooq U; Saeed H; Khan AM; Zubair A; Qamar I; Begum G Use of Gabapentin in the Treatment of Substance Use and Psychiatric Disorders: A Systematic Review.2019. DOI: 10.3389/fpsyt.2019.00228
59. McCarty D; Braude L; Lyman DR; Dougherty RH; Daniels AS; Ghose SS; Delphin-Rittmon ME Substance abuse intensive outpatient programs: assessing the evidence.2014. DOI: 10.1176/appi.ps.201300249
60. Klimas J; Field CA; Cullen W; O'Gorman CS; Glynn LG; Keenan E; Saunders J; Bury G; Dunne C Psychosocial interventions to reduce alcohol consumption in concurrent problem alcohol and illicit drug users: Cochrane Review.2013. DOI: 10.1186/2046-4053-2-3
61. Blaga OM; Vasilescu L; Chereches RM Use and effectiveness of behavioural economics in interventions for lifestyle risk factors of non-communicable diseases: a systematic review with policy implications.2018. DOI: 10.1177/1757913917720233
62. Nadkarni A; Endsley P; Bhatia U; Fuhr DC; Noorani A; Naik A; Murthy P; Velleman R Community detoxification for alcohol dependence: A systematic review.2017. DOI: 10.1111/dar.12440
63. Bywood PT; Lunnay B; Roche AM Strategies for facilitating change in alcohol and other drugs (AOD) professional practice: a systematic review of the effectiveness of reminders and feedback.2008. DOI: 10.1080/09595230802245535
64. Jones JL; Mateus CF; Malcolm RJ; Brady KT; Back SE Efficacy of Ketamine in the Treatment of Substance Use Disorders: A Systematic Review.2018. DOI: 10.3389/fpsyt.2018.00277
65. Dranitsaris G; Selby P; Negrete JC Meta-analyses of placebo-controlled trials of acamprosate for the treatment of alcohol dependence: impact of the combined pharmacotherapies and behavior interventions study.2009. DOI: 10.1097/ADM.0b013e318182d890
66. Ghita A; Gutiérrez-Maldonado J Applications of virtual reality in individuals with alcohol misuse: A systematic review.2018. DOI: 10.1016/j.addbeh.2018.01.036
67. Mcmurran, M. Individual-level interventions for alcohol-related violence: A rapid evidence assessment2011. DOI: 10.1002/cbm.821
68. Dzidowska M; Lee KSK; Wylie C; Bailie J; Percival N; Conigrave JH; Hayman N; Conigrave KM A systematic review of approaches to improve practice, detection and treatment of unhealthy alcohol use in primary health care: a role for continuous quality improvement.2020. DOI: 10.1186/s12875-020-1101-x
69. Nilsen P; Baird J; Mello MJ; Nirenberg T; Woolard R; Bendtsen P; Longabaugh R A systematic review of emergency care brief alcohol interventions for injury patients.2008. DOI: 10.1016/j.jsat.2007.09.008
70. Newton, N.C.; Champion, K.E.; Slade, T.; Chapman, C.; Stapinski, L.; Koning, I.; Tonks, Z.; Teesson, M. A systematic review of combined student- and parent-based programs to prevent alcohol and other drug use among adolescents2017. DOI: 10.1111/dar.12497
71. Champassak, S.L.; Miller, M.; Goggin, K. Motivational Interviewing for Adolescents in the Emergency Department2015. DOI: 10.1016/j.cpem.2015.04.004
72. Kaner, E.F.S.; Brown, N.; Jackson, K. A systematic review of the impact of brief interventions on substance use and co-morbid physical and mental health conditions2011. DOI: 10.1080/17523281.2011.533449
73. Drake, R.E.; O'Neal, E.L.; Wallach, M.A. A systematic review of psychosocial research on psychosocial interventions for people with co-occurring severe mental and substance use disorders2008. DOI: 10.1016/j.jsat.2007.01.011
74. Engle, B.; MacGowan, M.J. A critical review of adolescent substance abuse group treatments2009. DOI: 10.1080/15433710802686971
75. Bhatia, U.; Nadkarni, A.; Murthy, P.; Rao, R.; Crome, I. Recent advances in treatment for older people with substance use problems: An updated systematic and narrative review2015. DOI: 10.1016/j.eurger.2015.07.001
76. Turna J; Syan SK; Frey BN; Rush B; Costello MJ; Weiss M; MacKillop J Cannabidiol as a Novel Candidate Alcohol Use Disorder Pharmacotherapy: A Systematic Review.2019. DOI: 10.1111/acer.13964
77. Bhochhibhoya A; Hayes L; Branscum P; Taylor L The Use of the Internet for Prevention of Binge Drinking Among the College Population: A Systematic Review of Evidence.2015. DOI: 10.1093/alcalc/agv047
78. Nourredine M; Jurek L; Angerville B; Longuet Y; de Ternay J; Derveaux A; Rolland B Use of Topiramate in the Spectrum of Addictive and Eating Disorders: A Systematic Review Comparing Treatment Schemes, Efficacy, and Safety Features.2021. DOI: 10.1007/s40263-020-00780-y
79. Purcell-Khodr GC; Lee KSK; Conigrave JH; Webster E; Conigrave KM What can primary care services do to help First Nations people with unhealthy alcohol use? A systematic review: Australia, New Zealand, USA and Canada.2020. DOI: 10.1186/s13722-020-00204-8
80. Neven, A.; Dumont, G. The efficacy of baclofen in alcohol dependence2019. DOI:
81. Moy, I.; Crome, P.; Crome, I.; Fisher, M. Systematic and narrative review of treatment for older people with substance problems2011. DOI: 10.1016/j.eurger.2011.06.004
82. McGinnes RA; Hutton JE; Weiland TJ; Fatovich DM; Egerton-Warburton D Review article: Effectiveness of ultra-brief interventions in the emergency department to reduce alcohol consumption: A systematic review.2016. DOI: 10.1111/1742-6723.12624
83. Hurzeler T; Giannopoulos V; Uribe G; Louie E; Haber P; Morley KC Psychosocial Interventions for Reducing Suicidal Behaviour and Alcohol Consumption in Patients With Alcohol Problems: A Systematic Review of Randomized Controlled Trials.2021. DOI: 10.1093/alcalc/agaa094
84. Miller PM; Book SW; Stewart SH Medical treatment of alcohol dependence: a systematic review.2011. DOI: 10.2190/PM.42.3.b
85. Kingsland M; Wiggers JH; Vashum KP; Hodder RK; Wolfenden L Interventions in sports settings to reduce risky alcohol consumption and alcohol-related harm: a systematic review.2016. DOI: 10.1186/s13643-016-0183-y
86. Valdez LA; Flores M; Ruiz J; Oren E; Carvajal S; Garcia DO Gender and Cultural Adaptations for Diversity: A Systematic Review of Alcohol and Substance Abuse Interventions for Latino Males.2018. DOI: 10.1080/10826084.2017.1417999
87. Oliveira C; Pereira A; Vagos P; Nóbrega C; Gonçalves J; Afonso B Effectiveness of Mobile App-Based Psychological Interventions for College Students: A Systematic Review of the Literature.2021. DOI: 10.3389/fpsyg.2021.647606
88. Platt L; Melendez-Torres GJ; O'Donnell A; Bradley J; Newbury-Birch D; Kaner E; Ashton C How effective are brief interventions in reducing alcohol consumption: do the setting, practitioner group and content matter? Findings from a systematic review and metaregression analysis.2016. DOI: 10.1136/bmjopen-2016-011473
89. Hobden B; Bryant J; Carey M; Baker AL; Farrell M; Oldmeadow C; Mattick RP; Shakeshaft A; Sanson-Fisher R Finding the optimal treatment model: A systematic review of treatment for co-occurring alcohol misuse and depression.2018. DOI: 10.1177/0004867418758922
90. Khadjesari Z; Murray E; Hewitt C; Hartley S; Godfrey C Can stand-alone computer-based interventions reduce alcohol consumption? A systematic review.2011. DOI: 10.1111/j.1360-0443.2010.03214.x
91. Merz V; Baptista J; Haller DM Brief interventions to prevent recurrence and alcohol-related problems in young adults admitted to the emergency ward following an alcohol-related event: a systematic review.2015. DOI: 10.1136/jech-2014-204824
92. Lenaerts E; Matheï C; Matthys F; Zeeuws D; Pas L; Anderson P; Aertgeerts B Continuing care for patients with alcohol use disorders: a systematic review.2014. DOI: 10.1016/j.drugalcdep.2013.10.030
93. Korczak D; Steinhauser G; Dietl M Prevention of alcohol misuse among children, youths and young adults.2011. DOI: 10.3205/hta000095
94. Hesse, M. Integrated psychological treatment for substance use and co-morbid anxiety or depression vs. treatment for substance use alone. A systematic review of the published literature2009. DOI: 10.1186/1471-244X-9-6
95. Korecki JR; Schwebel FJ; Votaw VR; Witkiewitz K Mindfulness-based programs for substance use disorders: a systematic review of manualized treatments.2020. DOI: 10.1186/s13011-020-00293-3
96. Mujcic A; Blankers M; Bommelé J; Boon B; Berman AH; Verdonck-de Leeuw IM; van Laar M; Engels R [Not Available].2020. DOI: 10.1002/pon.5261
97. Whitlock EP; Polen MR; Green CA; Orleans T; Klein J Behavioral counseling interventions in primary care to reduce risky/harmful alcohol use by adults: a summary of the evidence for the U.S. Preventive Services Task Force.2004. DOI: 10.7326/0003-4819-140-7-200404060-00017
98. Joseph, J.; Basu, D. Efficacy of Brief Interventions in Reducing Hazardous or Harmful Alcohol Use in Middle-Income Countries: Systematic Review of Randomized Controlled Trials2016. DOI: 10.1093/alcalc/agw054
99. Chebli JL; Blaszczynski A; Gainsbury SM Internet-Based Interventions for Addictive Behaviours: A Systematic Review.2016. DOI: 10.1007/s10899-016-9599-5
100. Garbutt JC; Greenblatt AM; West SL; Morgan LC; Kampov-Polevoy A; Jordan HS; Bobashev GV Clinical and biological moderators of response to naltrexone in alcohol dependence: a systematic review of the evidence.2014. DOI: 10.1111/add.12557
101. Neven A; de Jong J; Pieterse BH [The pharmacological treatment of PTSD and alcohol use disorder: a systematic literature review].2019. DOI:
102. Wray TB; Grin B; Dorfman L; Glynn TR; Kahler CW; Marshall BD; van den Berg JJ; Zaller ND; Bryant KJ; Operario D Systematic review of interventions to reduce problematic alcohol use in men who have sex with men.2016. DOI: 10.1111/dar.12271
103. Rombouts SA; Conigrave JH; Saitz R; Louie E; Haber P; Morley KC Evidence based models of care for the treatment of alcohol use disorder in primary health care settings: a systematic review.2020. DOI: 10.1186/s12875-020-01288-6
104. Newton AS; Dong K; Mabood N; Ata N; Ali S; Gokiert R; Vandermeer B; Tjosvold L; Hartling L; Wild TC Brief emergency department interventions for youth who use alcohol and other drugs: a systematic review.2013. DOI: 10.1097/PEC.0b013e31828ed325
105. Shingler E; Robles LA; Perry R; Penfold C; Ness AR; Thomas S; Lane JA; Martin RM Systematic review evaluating randomized controlled trials of smoking and alcohol cessation interventions in people with head and neck cancer and oral dysplasia.2018. DOI: 10.1002/hed.25138
106. Srivastava A; Kahan M; Ross S The effect of methadone maintenance treatment on alcohol consumption: a systematic review.2008. DOI: 10.1016/j.jsat.2007.04.001
107. Hyde J; Hankins M; Deale A; Marteau TM Interventions to increase self-efficacy in the context of addiction behaviours: a systematic literature review.2008. DOI: 10.1177/1359105308090933
108. Leichsenring, F.; Klein, S. Evidence for psychodynamic psychotherapy in specific mental disorders: A systematic review2014. DOI: 10.1080/02668734.2013.865428
109. Greutman, M.D.; Gales, M.A.; Gales, B.J. Gabapentin in alcohol dependence2015. DOI: 10.1177/8755122515575543
110. Farhadian N; Moradi S; Zamanian MH; Farnia V; Rezaeian S; Farhadian M; Shahlaei M Effectiveness of naltrexone treatment for alcohol use disorders in HIV: a systematic review.2020. DOI: 10.1186/s13011-020-00266-6
111. Muzyk AJ; Rivelli SK; Gagliardi JP Defining the role of baclofen for the treatment of alcohol dependence: a systematic review of the evidence.2012. DOI: 10.2165/11597320-000000000-00000
112. Sileo KM; Miller AP; Huynh TA; Kiene SM A systematic review of interventions for reducing heavy episodic drinking in sub-Saharan African settings.2020. DOI: 10.1371/journal.pone.0242678
113. Erng MN; Smirnov A; Reid N Prevention of Alcohol-Exposed Pregnancies and Fetal Alcohol Spectrum Disorder Among Pregnant and Postpartum Women: A Systematic Review.2020. DOI: 10.1111/acer.14489
114. Emmen MJ; Schippers GM; Bleijenberg G; Wollersheim H Effectiveness of opportunistic brief interventions for problem drinking in a general hospital setting: systematic review.2004. DOI: 10.1136/bmj.37956.562130.EE
115. Diestelkamp S; Drechsel M; Baldus C; Wartberg L; Arnaud N; Thomasius R Brief in Person Interventions for Adolescents and Young Adults Following Alcohol-Related Events in Emergency Care: A Systematic Review and European Evidence Synthesis.2016. DOI: 10.1159/000435877
116. Naglich AC; Lin A; Wakhlu S; Adinoff BH Systematic Review of Combined Pharmacotherapy for the Treatment of Alcohol Use Disorder in Patients Without Comorbid Conditions.2018. DOI: 10.1007/s40263-017-0484-2
117. Garbutt JC; West SL; Carey TS; Lohr KN; Crews FT Pharmacological treatment of alcohol dependence: a review of the evidence.1999. DOI: 10.1001/jama.281.14.1318
118. Louie E; Barrett EL; Baillie A; Haber P; Morley KC A systematic review of evidence-based practice implementation in drug and alcohol settings: applying the consolidated framework for implementation research framework.2021. DOI: 10.1186/s13012-021-01090-7
119. Hadjistavropoulos HD; Mehta S; Wilhelms A; Keough MT; Sundström C A systematic review of internet-delivered cognitive behavior therapy for alcohol misuse: study characteristics, program content and outcomes.2020. DOI: 10.1080/16506073.2019.1663258
120. Joseph J; Basu D; Dandapani M; Krishnan N Are nurse-conducted brief interventions (NCBIs) efficacious for hazardous or harmful alcohol use? A systematic review.2014. DOI: 10.1111/inr.12096
121. Fuentes, J.J.; Fonseca, F.; Elices, M.; FarrÃ©, M.; Torrens, M. Therapeutic Use of LSD in Psychiatry: A Systematic Review of Randomized-Controlled Clinical Trials2020. DOI: 10.3389/fpsyt.2019.00943
122. Klimas J; Field CA; Cullen W; O'Gorman CS; Glynn LG; Keenan E; Saunders J; Bury G; Dunne C Psychosocial interventions to reduce alcohol consumption in concurrent problem alcohol and illicit drug users.2012. DOI: 10.1002/14651858.CD009269.pub2
123. Jaehne A; Loessl B; Frick K; Berner M; Hulse G; Balmford J The efficacy of stepped care models involving psychosocial treatment of alcohol use disorders and nicotine dependence: a systematic review of the literature.2012. DOI: 10.2174/1874473711205010041
124. Burucker J; Kropp S [Levels of evidence in drug therapy for alcohol use disorders and illicit drug use].2012. DOI: 10.1055/s-0032-1325452
125. Wobrock T; D'Amelio R; Falkai P [Pharmacotherapy of schizophrenia and comorbid substance use disorder. A systematic review].2008. DOI: 10.1007/s00115-007-2310-4
126. Hutton A; Prichard I; Whitehead D; Thomas S; Rubin M; Sloand E; Powell TW; Frisch K; Newman P; Goodwin Veenema T mHealth Interventions to Reduce Alcohol Use in Young People: A Systematic Review of the Literature.2020. DOI: 10.1080/24694193.2019.1616008
127. Lin LA; Casteel D; Shigekawa E; Weyrich MS; Roby DH; McMenamin SB Telemedicine-delivered treatment interventions for substance use disorders: A systematic review.2019. DOI: 10.1016/j.jsat.2019.03.007
128. Ghosh A; Mahintamani T; Choudhury S; Sharma N; Das S The Effectiveness of Non-Benzodiazepine, Non-Barbiturate Medications for Alcohol Withdrawal Syndrome: A Rapid Systematic Review.2021. DOI: 10.1093/alcalc/agaa125
129. White A; Kavanagh D; Stallman H; Klein B; Kay-Lambkin F; Proudfoot J; Drennan J; Connor J; Baker A; Hines E; Young R Online alcohol interventions: a systematic review.2010. DOI: 10.2196/jmir.1479
130. Samawi L; Williams PP; Myers B; Fuhr DC Effectiveness of psychological interventions to reduce alcohol consumption among pregnant and postpartum women: a systematic review.2021. DOI: 10.1007/s00737-020-01100-5
131. Tofighi B; Nicholson JM; McNeely J; Muench F; Lee JD Mobile phone messaging for illicit drug and alcohol dependence: A systematic review of the literature.2017. DOI: 10.1111/dar.12535
132. Jackson C; Geddes R; Haw S; Frank J Interventions to prevent substance use and risky sexual behaviour in young people: a systematic review.2012. DOI: 10.1111/j.1360-0443.2011.03751.x
133. Tansil KA; Esser MB; Sandhu P; Reynolds JA; Elder RW; Williamson RS; Chattopadhyay SK; Bohm MK; Brewer RD; McKnight-Eily LR; Hungerford DW; Toomey TL; Hingson RW; Fielding JE Alcohol Electronic Screening and Brief Intervention: A Community Guide Systematic Review.2016. DOI: 10.1016/j.amepre.2016.04.013
134. Young CL; Trapani K; Dawson S; O'Neil A; Kay-Lambkin F; Berk M; Jacka FN Efficacy of online lifestyle interventions targeting lifestyle behaviour change in depressed populations: A systematic review.2018. DOI: 10.1177/0004867418788659
135. Perez-Macia V; Martinez-Cortes M; Mesones J; Segura-Trepichio M; Garcia-Fernandez L Monitoring and Improving Naltrexone Adherence in Patients with Substance Use Disorder.2021. DOI: 10.2147/PPA.S277861
136. Holleck JL; Merchant N; Gunderson CG Symptom-Triggered Therapy for Alcohol Withdrawal Syndrome: a Systematic Review and Meta-analysis of Randomized Controlled Trials.2019. DOI: 10.1007/s11606-019-04899-7
137. Romero Rodríguez EM; Pérula de Torres LÁ; Linares Ruiz A; Fernández García JÁ; Parras Rejano JM; Roldán Villalobos A [Effectiveness of training programs on alcohol consumption in the Primary Care setting: Systematic review].2019. DOI: 10.1016/j.aprim.2018.07.004
138. Kaner EF; Dickinson HO; Beyer F; Pienaar E; Schlesinger C; Campbell F; Saunders JB; Burnand B; Heather N The effectiveness of brief alcohol interventions in primary care settings: a systematic review.2009. DOI: 10.1111/j.1465-3362.2009.00071.x
139. Batschelet HM; Stein M; Tschuemperlin RM; Soravia LM; Moggi F Alcohol-Specific Computerized Interventions to Alter Cognitive Biases: A Systematic Review of Effects on Experimental Tasks, Drinking Behavior, and Neuronal Activation.2019. DOI: 10.3389/fpsyt.2019.00871
140. Barrio P; Gual A Patient-centered care interventions for the management of alcohol use disorders: a systematic review of randomized controlled trials.2016. DOI: 10.2147/PPA.S109641
141. Ashford, Robert D.; Bergman, Brandon G.; Kelly, John F.; Curtis, Brenda Systematic review: Digital recovery support services used to support substance use disorder recovery.2020. DOI: 10.1002/hbe2.148
142. Carrasco MA; Esser MB; Sparks A; Kaufman MR HIV-Alcohol Risk Reduction Interventions in Sub-Saharan Africa: A Systematic Review of the Literature and Recommendations for a Way Forward.2016. DOI: 10.1007/s10461-015-1233-5
143. Allen ML; Garcia-Huidobro D; Porta C; Curran D; Patel R; Miller J; Borowsky I Effective Parenting Interventions to Reduce Youth Substance Use: A Systematic Review.2016. DOI: 10.1542/peds.2015-4425
144. Rose-Clarke K; Bentley A; Marston C; Prost A Peer-facilitated community-based interventions for adolescent health in low- and middle-income countries: A systematic review.2019. DOI: 10.1371/journal.pone.0210468
145. Heitmann J; Bennik EC; van Hemel-Ruiter ME; de Jong PJ The effectiveness of attentional bias modification for substance use disorder symptoms in adults: a systematic review.2018. DOI: 10.1186/s13643-018-0822-6
146. Khan A; Tansel A; White DL; Kayani WT; Bano S; Lindsay J; El-Serag HB; Kanwal F Efficacy of Psychosocial Interventions in Inducing and Maintaining Alcohol Abstinence in Patients With Chronic Liver Disease: A Systematic Review.2016. DOI: 10.1016/j.cgh.2015.07.047
147. Mdege ND; Fayter D; Watson JM; Stirk L; Sowden A; Godfrey C Interventions for reducing alcohol consumption among general hospital inpatient heavy alcohol users: a systematic review.2013. DOI: 10.1016/j.drugalcdep.2013.01.023
148. Fergie L; Campbell KA; Coleman-Haynes T; Ussher M; Cooper S; Coleman T Identifying Effective Behavior Change Techniques for Alcohol and Illicit Substance Use During Pregnancy: A Systematic Review.2019. DOI: 10.1093/abm/kay085
149. Brambilla, R.; Vigna-Taglianti, F.; Avanzi, G.; Faggiano, F.; Leone, M. Gamma-hydroxybutyrate (GHB) for mid/long term treatment of alcohol dependence: A systematic review2012. DOI:
150. Rosário F; Santos MI; Angus K; Pas L; Ribeiro C; Fitzgerald N Factors influencing the implementation of screening and brief interventions for alcohol use in primary care practices: a systematic review using the COM-B system and Theoretical Domains Framework.2021. DOI: 10.1186/s13012-020-01073-0
151. Simioni N; Cottencin O; Rolland B Interventions for Increasing Subsequent Alcohol Treatment Utilisation Among Patients with Alcohol Use Disorders from Somatic Inpatient Settings: A Systematic Review.2015. DOI: 10.1093/alcalc/agv017
152. Brambilla, Romeo; Vigna-Taglianti, Federica; Avanzi, Giancarlo; Faggiano, Fabrizio; Leone, Maurizio II gamma-idrossibutirrato (GHB) nel trattamento a medio/lungo termine della dipendenza da alcol: Una revisione sistematica. [Gamma-hydroxybutyrate (GHB) for mid/long term treatment, of alcohol dependence: A systematic review.]2012. DOI:
153. Boniface S; Malet-Lambert I; Coleman R; Deluca P; Donoghue K; Drummond C; Khadjesari Z The Effect of Brief Interventions for Alcohol Among People with Comorbid Mental Health Conditions: A Systematic Review of Randomized Trials and Narrative Synthesis.2018. DOI: 10.1093/alcalc/agx111
154. Bewick BM; Trusler K; Barkham M; Hill AJ; Cahill J; Mulhern B The effectiveness of web-based interventions designed to decrease alcohol consumption--a systematic review.2008. DOI: 10.1016/j.ypmed.2008.01.005
155. Gupta S; Jhanjee S; Dhawan A Effectiveness of Interventions Based on Yogic Breathing Practices (IB-YBP) on Substance Use Disorders-A Systematic Review of the Randomized Control Trials and Quasi-Experimental Trials.2021. DOI: 10.1080/10826084.2021.1942056
156. Li, Q.; Babor, T.F.; Zeigler, D.; Xuan, Z.; Morisky, D.; Hovell, M.F.; Nelson, T.F.; Shen, W.; Li, B. Health promotion interventions and policies addressing excessive alcohol use: a systematic review of national and global evidence as a guide to health-care reform in China2015. DOI: 10.1111/add.12784
157. Preusse M; Neuner F; Ertl V Effectiveness of Psychosocial Interventions Targeting Hazardous and Harmful Alcohol Use and Alcohol-Related Symptoms in Low- and Middle-Income Countries: A Systematic Review.2020. DOI: 10.3389/fpsyt.2020.00768
158. Kahan M; Wilson L; Becker L Effectiveness of physician-based interventions with problem drinkers: a review.1995. DOI:
159. Bahadoor R; Alexandre JM; Fournet L; Gellé T; Serre F; Auriacombe M Inventory and Analysis of Controlled Trials of Mobile Phone Applications Targeting Substance Use Disorders: A Systematic Review.2021. DOI: 10.3389/fpsyt.2021.622394
160. Dunn, C.; Deroo, L.; Rivara, F.P. The use of brief interventions adapted from motivational interviewing across behavioral domains: A systematic review2001. DOI: 10.1046/j.1360-0443.2001.961217253.x
161. Leske S; Harris MG; Charlson FJ; Ferrari AJ; Baxter AJ; Logan JM; Toombs M; Whiteford H Systematic review of interventions for Indigenous adults with mental and substance use disorders in Australia, Canada, New Zealand and the United States.2016. DOI: 10.1177/0004867416662150
162. Song T; Qian S; Yu P Mobile Health Interventions for Self-Control of Unhealthy Alcohol Use: Systematic Review.2019. DOI: 10.2196/10899
163. Bassuk EL; Hanson J; Greene RN; Richard M; Laudet A Peer-Delivered Recovery Support Services for Addictions in the United States: A Systematic Review.2016. DOI: 10.1016/j.jsat.2016.01.003
164. Berlin, R.K.; Butler, P.M.; Perloff, M.D. Gabapentin therapy in psychiatric disorders: A systematic review2015. DOI: 10.4088/PCC.15r01821
165. Cohen J; Dervaux A; Laqueille X [Topiramate in substance-related and addictive disorders].2014. DOI: 10.1016/j.lpm.2014.02.030
166. Bottlender M; Köhler J; Soyka M [The effectiveness of psychosocial treatment approaches for alcohol dependence--a review].2006. DOI: 10.1055/s-2004-830282
167. Li Q; Babor TF; Zeigler D; Xuan Z; Morisky D; Hovell MF; Nelson TF; Shen W; Li B Health promotion interventions and policies addressing excessive alcohol use: a systematic review of national and global evidence as a guide to health-care reform in China.2015. DOI: 10.1111/add.12784
168. McGovern, R.; Newham, J.J.; Addison, M.T.; Hickman, M.; Kaner, E.F.S. Effectiveness of psychosocial interventions for reducing parental substance misuse2021. DOI: 10.1002/14651858.CD012823.pub2
169. Chiesa A Vipassana meditation: systematic review of current evidence.2010. DOI: 10.1089/acm.2009.0362
170. Tait RJ; Hulse GK A systematic review of the effectiveness of brief interventions with substance using adolescents by type of drug.2003. DOI: 10.1080/0959523031000154481
171. James E; Freund M; Booth A; Duncan MJ; Johnson N; Short CE; Wolfenden L; Stacey FG; Kay-Lambkin F; Vandelanotte C Comparative efficacy of simultaneous versus sequential multiple health behavior change interventions among adults: A systematic review of randomised trials.2016. DOI: 10.1016/j.ypmed.2016.06.012
172. Baker AL; Thornton LK; Hiles S; Hides L; Lubman DI Psychological interventions for alcohol misuse among people with co-occurring depression or anxiety disorders: a systematic review.2012. DOI: 10.1016/j.jad.2011.08.004
173. Dimova ED; Elliott L; Frankis J; Drabble L; Wiencierz S; Emslie C Alcohol interventions for LGBTQ+ adults: A systematic review.2021. DOI: 10.1111/dar.13358
174. Kazemi DM; Li S; Levine MJ; Auten B; Granson M Systematic Review of Smartphone Apps as a mHealth Intervention to Address Substance Abuse in Adolescents and Adults.2021. DOI: 10.1097/JAN.0000000000000416
175. Cho SH; Whang WW Acupuncture for alcohol dependence: a systematic review.2009. DOI: 10.1111/j.1530-0277.2009.00959.x
176. Minozzi S; Saulle R; Rösner S Baclofen for alcohol use disorder.2018. DOI: 10.1002/14651858.CD012557.pub2
177. Southern C; Lloyd C; Liu J; Wang C; Zhang T; Bland M; MacPherson H Acupuncture as an intervention to reduce alcohol dependency: a systematic review and meta-analysis.2016. DOI: 10.1186/s13020-016-0119-4
178. Newbury-Birch D; Ferguson J; Landale S; Giles EL; McGeechan GJ; Gill C; Stockdale KJ; Holloway A A Systematic Review of the Efficacy of Alcohol Interventions for Incarcerated People.2018. DOI: 10.1093/alcalc/agy032
179. Webb G; Shakeshaft A; Sanson-Fisher R; Havard A A systematic review of work-place interventions for alcohol-related problems.2009. DOI: 10.1111/j.1360-0443.2008.02472.x
180. Giusto A; Puffer E A systematic review of interventions targeting men's alcohol use and family relationships in low- and middle-income countries.2018. DOI: 10.1017/gmh.2017.32
181. Landy MS; Davey CJ; Quintero D; Pecora A; McShane KE A Systematic Review on the Effectiveness of Brief Interventions for Alcohol Misuse among Adults in Emergency Departments.2016. DOI: 10.1016/j.jsat.2015.08.004
182. Baker AL; Hiles SA; Thornton LK; Hides L; Lubman DI A systematic review of psychological interventions for excessive alcohol consumption among people with psychotic disorders.2012. DOI: 10.1111/j.1600-0447.2012.01885.x
183. Byaruhanga J; Atorkey P; McLaughlin M; Brown A; Byrnes E; Paul C; Wiggers J; Tzelepis F Effectiveness of Individual Real-Time Video Counseling on Smoking, Nutrition, Alcohol, Physical Activity, and Obesity Health Risks: Systematic Review.2020. DOI: 10.2196/18621
184. Barnett NP; Read JP Mandatory alcohol intervention for alcohol-abusing college students: a systematic review.2005. DOI: 10.1016/j.jsat.2005.05.007
185. Kuntsche S; Kuntsche E Parent-based interventions for preventing or reducing adolescent substance use - A systematic literature review.2016. DOI: 10.1016/j.cpr.2016.02.004
186. Fowler LA; Holt SL; Joshi D Mobile technology-based interventions for adult users of alcohol: A systematic review of the literature.2016. DOI: 10.1016/j.addbeh.2016.06.008
187. Giesen ES; Deimel H; Bloch W Clinical exercise interventions in alcohol use disorders: a systematic review.2015. DOI: 10.1016/j.jsat.2014.12.001
188. Meads C; Ting S; Dretzke J; Bayliss S A systematic review of the clinical and cost-effectiveness of psychological therapy involving family and friends in alcohol misuse or dependence2007. DOI:
189. O Rourke L; Humphris G; Baldacchino A Electronic communication based interventions for hazardous young drinkers: A systematic review.2016. DOI: 10.1016/j.neubiorev.2016.07.021
190. Buoli M; Grassi S; Ciappolino V; Serati M; Altamura AC The Use of Zonisamide for the Treatment of Psychiatric Disorders: A Systematic Review.2017. DOI: 10.1097/WNF.0000000000000208
191. Kodadek LM; Freeman JJ; Tiwary D; Drake MD; Schroeder ME; Dultz L; White C; Abdel Aziz H; Crandall M; Como JJ; Rattan R Alcohol-related trauma reinjury prevention with hospital-based screening in adult populations: An Eastern Association for the Surgery of Trauma evidence-based systematic review.2020. DOI: 10.1097/TA.0000000000002501
192. Klimas J; Tobin H; Field CA; O'Gorman CS; Glynn LG; Keenan E; Saunders J; Bury G; Dunne C; Cullen W Psychosocial interventions to reduce alcohol consumption in concurrent problem alcohol and illicit drug users.2014. DOI: 10.1002/14651858.CD009269.pub3
193. Ashton, L.M.; Morgan, P.J.; Hutchesson, M.J.; Rollo, M.E.; Young, M.D.; Collins, C.E. A systematic review of SNAPO (Smoking, Nutrition, Alcohol, Physical activity and Obesity) randomized controlled trials in young adult men2015. DOI: 10.1016/j.ypmed.2015.09.005
194. Roozen HG; Boulogne JJ; van Tulder MW; van den Brink W; De Jong CA; Kerkhof AJ A systematic review of the effectiveness of the community reinforcement approach in alcohol, cocaine and opioid addiction.2004. DOI: 10.1016/j.drugalcdep.2003.12.006
195. Ashenden R; Silagy C; Weller D A systematic review of the effectiveness of promoting lifestyle change in general practice.1997. DOI: 10.1093/fampra/14.2.160
196. Fernandez AC; Claborn KR; Borsari B A systematic review of behavioural interventions to reduce preoperative alcohol use.2015. DOI: 10.1111/dar.12285
197. Riemsma, R.P.; Pattenden, J.; Bridle, C.; Sowden, A.J.; Mather, L.; Watt, I.S.; Walker, A. A systematic review of the effectiveness of interventions based on a stages-of-change approach to promote individual behaviour change2002. DOI: 10.3310/hta6240
198. Castrén, S.; Mäkelä, N.; Alho, H. Selecting an appropriate alcohol pharmacotherapy: Review of recent findings2019. DOI: 10.1097/YCO.0000000000000512
199. Oâ€™Connor EA; Perdue LA; Senger CA; Rushkin M; Patnode CD; Bean SI; Jonas DE 2018. DOI:
200. Lee, N.K.; Roche, A.M.; Duraisingam, V.; Fischer, J.; Cameron, J.; Pidd, K. A systematic review of alcohol interventions among workers in male-dominated industries2014. DOI: 10.1089/jomh.2014.0008
201. Oosterveen E; Tzelepis F; Ashton L; Hutchesson MJ A systematic review of eHealth behavioral interventions targeting smoking, nutrition, alcohol, physical activity and/or obesity for young adults.2017. DOI: 10.1016/j.ypmed.2017.01.009
202. Wigham S; Bauer A; Robalino S; Ferguson J; Burke A; Newbury-Birch D A systematic review of the effectiveness of alcohol brief interventions for the UK military personnel moving back to civilian life.2017. DOI: 10.1136/jramc-2016-000712
203. Jiang S; Wu L; Gao X Beyond face-to-face individual counseling: A systematic review on alternative modes of motivational interviewing in substance abuse treatment and prevention.2017. DOI: 10.1016/j.addbeh.2017.05.023
204. Batschelet, H.M.; Stein, M.; Tschuemperlin, R.M.; Soravia, L.M.; Moggi, F. Alcohol-Specific Computerized Interventions to Alter Cognitive Biases: A Systematic Review of Effects on Experimental Tasks, Drinking Behavior, and Neuronal Activation2020. DOI: 10.3389/fpsyt.2019.00871
205. Brown TJ; Todd A; O’Malley CL; Moore HJ; Husband AK; Bambra C; Kasim A; Sniehotta FF; Steed L; Summerbell CD 2016. DOI: 10.3310/phr04020
206. Bouza C; Angeles M; Munoz A; Amate JM Efficacy and safety of naltrexone and acamprosate in the treatment of alcohol dependence: a systematic review.2004. DOI: 10.1111/j.1360-0443.2004.00763.x
207. Bollen, Z.; Dormal, V.; Maurage, P. How Should Transcranial Direct Current Stimulation be Used in Populations With Severe Alcohol Use Disorder? A Clinically Oriented Systematic Review2021. DOI: 10.1177/15500594211001212
208. Andersen LAK; Munk S; Nielsen AS; Bilberg R What is known about treatment aimed at indigenous people suffering from alcohol use disorder?2019. DOI: 10.1080/15332640.2019.1679317
209. Reangsing C; Wimolphan P; Wongsuraprakit S; Oerther S Effects of Mindfulness-Based Interventions on Depressive Symptoms and Alcohol Craving in Individuals With Comorbid Alcohol Use Disorder and Depression: A Systematic Review.2021. DOI: 10.3928/02793695-20210819-04
210. Roozen HG; de Waart R; van der Windt DA; van den Brink W; de Jong CA; Kerkhof AJ A systematic review of the effectiveness of naltrexone in the maintenance treatment of opioid and alcohol dependence.2006. DOI: 10.1016/j.euroneuro.2005.11.001
211. Huibers MJ; Beurskens AJ; Bleijenberg G; van Schayck CP Psychosocial interventions by general practitioners.2007. DOI: 10.1002/14651858.CD003494.pub2
212. Aubin HJ; Daeppen JB Emerging pharmacotherapies for alcohol dependence: a systematic review focusing on reduction in consumption.2013. DOI: 10.1016/j.drugalcdep.2013.04.025
213. Madhombiro M; Musekiwa A; January J; Chingono A; Abas M; Seedat S Psychological interventions for alcohol use disorders in people living with HIV/AIDS: a systematic review.2019. DOI: 10.1186/s13643-019-1176-4
214. Di Miceli M; Gronier B Pharmacology, Systematic Review and Recent Clinical Trials of Metadoxine.2018. DOI: 10.2174/1574887113666180227100217
215. Posadzki, P.; Choi, J.; Lee, M.S.; Ernst, E. Yoga for addictions: A systematic review of randomised clinical trials2014. DOI: 10.1111/fct.12080
216. Nair NK; Newton NC; Shakeshaft A; Wallace P; Teesson M A Systematic Review of Digital and Computer-Based Alcohol Intervention Programs in Primary Care.2015. DOI: 10.2174/1874473708666150916113538

**Exclusion reason 5:** meta-analysis has been updated (most recent version only included)

1. McQueen J; Howe TE; Allan L; Mains D. Brief interventions for heavy alcohol users admitted to general hospital wards. 2009. DOI: 10.1002/14651858.CD005191.pub2
2. Liu J; Wang LN. Baclofen for alcohol withdrawal. 2013. DOI: 10.1002/14651858.CD008502.pub3
3. Polycarpou A; Papanikolaou P; Ioannidis JP; Contopoulos-Ioannidis DG. Anticonvulsants for alcohol withdrawal. 2005. DOI: 10.1002/14651858.CD005064.pub2
4. Foxcroft DR; Coombes L; Wood S; Allen D; Almeida Santimano NM. Motivational interviewing for alcohol misuse in young adults. 2014. DOI: 10.1002/14651858.CD007025.pub2
5. Liu, J.; Wang, L.-N.. Baclofen for alcohol withdrawal 2017. DOI: 10.1002/14651858.CD008502.pub5
6. Ntais C; Pakos E; Kyzas P; Ioannidis JP. Benzodiazepines for alcohol withdrawal. 2005. DOI: 10.1002/14651858.CD005063.pub2
7. Cleary M; Hunt G; Matheson S; Siegfried N; Walter G. Psychosocial interventions for people with both severe mental illness and substance misuse. 2008. DOI: 10.1002/14651858.CD001088.pub2
8. Kaner EF; Beyer F; Dickinson HO; Pienaar E; Campbell F; Schlesinger C; Heather N; Saunders J; Burnand B. Effectiveness of brief alcohol interventions in primary care populations. 2007. DOI: 10.1002/14651858.CD004148.pub3
9. Liu J; Wang L. Baclofen for alcohol withdrawal. 2011. DOI: 10.1002/14651858.CD008502.pub2
